# Dendrimer end-terminal motif-dependent evasion of human complement and complement activation through IgM hitchhiking

**DOI:** 10.1038/s41467-021-24960-6

**Published:** 2021-08-11

**Authors:** Lin-Ping Wu, Mario Ficker, Jørn B. Christensen, Dmitri Simberg, Panagiotis N. Trohopoulos, Seyed M. Moghimi

**Affiliations:** 1 Centre for Pharmaceutical Nanotechnology and Nanotoxicology, Department of Pharmacy, University of Copenhagen, Copenhagen Ø, Denmark; 2 Department of Chemistry, University of Copenhagen, Frederiksberg C, Denmark; 3 Translational Bio-Nanosciences Laboratory, The Skaggs School of Pharmacy and Pharmaceutical Sciences, Department of Pharmaceutical Sciences, University of Colorado Anschutz Medical Campus, Aurora, CO USA; 4 Colorado Center for Nanomedicine and Nanosafety, University of Colorado Anschutz Medical Campus, Aurora, CO USA; 5 CosmoPHOS Ltd, Thessaloniki, Greece; 6 Present Address: Guangzhou Institute of Biomedicine and Health, Chinese Academy of Sciences, Guangzhou, People’s Republic of China; 7 Present Address: School of Pharmacy, King George VI Building, Newcastle University, Newcastle upon Tyne, UK; 8 Present Address: Colorado Center for Nanomedicine and Nanosafety, University of Colorado Anschutz Medical Campus, Aurora, CO USA; 9 Present Address: Translational and Clinical Research Institute, Faculty of Health and Medical Sciences, Framlington Place, Newcastle University, Newcastle upon Tyne, UK

**Keywords:** Biomedical materials, Complement cascade, Immunotoxicity

## Abstract

Complement is an enzymatic humoral pattern-recognition defence system of the body. Non-specific deposition of blood biomolecules on nanomedicines triggers complement activation through the alternative pathway, but complement-triggering mechanisms of nanomaterials with dimensions comparable to or smaller than many globular blood proteins are unknown. Here we study this using a library of <6 nm poly(amido amine) dendrimers bearing different end-terminal functional groups. Dendrimers are not sensed by C1q and mannan-binding lectin, and hence do not trigger complement activation through these pattern-recognition molecules. While, pyrrolidone- and carboxylic acid-terminated dendrimers fully evade complement response, and independent of factor H modulation, binding of amine-terminated dendrimers to a subset of natural IgM glycoforms triggers complement activation through lectin pathway-IgM axis. These findings contribute to mechanistic understanding of complement surveillance of dendrimeric materials, and provide opportunities for dendrimer-driven engineering of complement-safe nanomedicines and medical devices.

## Abstract (web summary)

Understanding nanomaterials interactions with complement is important for a number of applications. Here, the authors study the interaction of sub 6 nm dendrimers with complement and show the small dendrimers escape complement activation but do interact with IgM to trigger lectin-pathway complement activation.

## Introduction

The complement system is an evolutionary ancient complex pattern-recognition surveillance network of the innate immunity, which also plays multifaceted roles in stability, performance and safety of intravenously injected nanomedicines^1^. Nanomaterials, depending on their physicochemical characteristics trigger complement activation differently through any of the three known complement pathways (classical, lectin and alternative)^1^. For instance, the pristine surfaces of some nanoparticles in the blood are directly sensed by complement pattern-recognition molecules such as C1q and mannan-binding lectin (MBL), leading to complement activation through classical and lectin pathways, respectively^2–5^. In addition to this, non-specific deposition of some blood proteins on nanoparticles (the “biomolecule corona”) could result in exposure of antigenic epitopes through protein conformational changes. These antigenic epitopes are recognised by circulating antibodies, which subsequently serve as target for nascent C3b attack leading to alternative pathway activation by antibodies^6^.

While the interaction of many preclinical and clinical nanomedicines in 20–500 nm size ranges with the human complement system has been extensively studied^1,2^, not much is known on complement activation mechanisms of ultra-small architectures such as dendrimers^7^. Dendrimers have dimensions comparable to or smaller than many globular blood proteins (≤6 nm), and hence are unlikely to acquire a complex corona of blood proteins^8^ to potentiate complement activation^6^. Considering that dendrimers are gaining increasing interest in medicine^9–12^, a clear understanding of dendrimer—human complement system interaction could provide better opportunities for engineering of immunologically-safe dendrimer-based nanomedicines.

Dendrimers are highly ordered nanostructures that begin with a core and grow in a stepwise fashion into concentric layers^9–11^. This regularity produces a geometrically progressive growth in size and an exponential increase in tightly spaced terminal end-groups at the periphery^13^. Thus, unlike many nanoparticles with stochastic number and distribution of surface functional groups^1^, dendrimers display precise numbers of spatially defined surface functional motifs^9^. Morphologically, early generation (G) dendrimers such as G2 and G3 poly(amido amine) (PAMAM) dendrimers are open ellipsoids (“star-like” architectures), whereas later G dendrimers resemble “hard-spheres”^14,15^. Dendrimeric end-terminal motifs are typically spaced <1 nm from each other^16^. We propose that Angstrom-scale spacing arrangement of dendrimer end-terminal motifs might inherently endow dendrimers to escape sensing by complement pattern-recognition molecules thereby avoiding complement activation through classical and lectin pathways, since the globular target recognition heads in C1q and collectins such as MBL bind to target motifs spaced at 2–15 nm apart^17–20^. However, considering that dendrimers are non-rigid polyvalent entities, binding of dendrimers to other complement and blood proteins (e.g., via salt-bridges, hydrogen bonding, and electrostatic and hydrophobic forces) could trigger complement activation. For instance, non-specific interaction of dendrimers with the third complement protein (C3) might trigger the alternative pathway. A proteomic attempt suggested high G amine-terminated PAMAM dendrimers bind C3 in human plasma^7^; however, complement activation was not studied. On the other hand, dendrimer binding to some blood proteins might induce conformational changes in protein structure and expose regions [e.g., oligomannose glycans on immunoglobulin (Ig) M)^21^] that might trigger complement activation through any of the three established pathways.

In this work, we investigate the interaction between the human complement system and a library of increasing G (G2–G5) of PAMAM dendrimers bearing amine, carboxylic acid-hydroxyl (carboxy-Tris) and pyrrolidone end-group functionalities. The results show that dendrimers are not sensed by complement pattern-recognition molecules C1q and MBL, and do not trigger complement activation through them. Pyrrolidone- and carboxy-Tris-terminated dendrimers fully evade complement response and the results exclude a contributing role for the complement regulator protein factor H (fH). While these dendrimers are not complement inhibitors and show no modulatory effect on complement functionality, matched generations of amine-terminated dendrimers hitchhike on natural glycosylated subtypes of IgM that binds to MBL. This triggers lectin pathway activation by IgM. We discuss these results in relation to the application of dendrimers in drug delivery, including the delivery of complement inhibitors, and suggest dendrimer-driven initiatives in the development of wider complement-safe nanomedicines and medical devices. We further expand our discussion beyond drug delivery and propose an additional complement escape mechanism in relation to virulent pathogens.

## Results

### Dendrimer synthesis and characterisation

The majority of biomedical applications of dendrimers is restricted to G3–G5 dendrimers^10,11^. Therefore, this study focuses on dendrimers up to G5. We chose dendrimers with amine, carboxylic acid-Tris and pyrrolidone end-terminal functionalities due to their water solubility and diverse post-modification flexibility applicable to nanomedicine^9–11^. We avoided all hydroxyl functionality, since with G4 and higher dendrimers, the structures significantly collapse as a result of extensive hydrogen bonding among terminal hydroxyl groups as well as between hydroxyl and interior amidoamine groups^22^. Thus, carboxy-Tris-terminated dendrimers were used as a compromise.

**Fig. 1:**
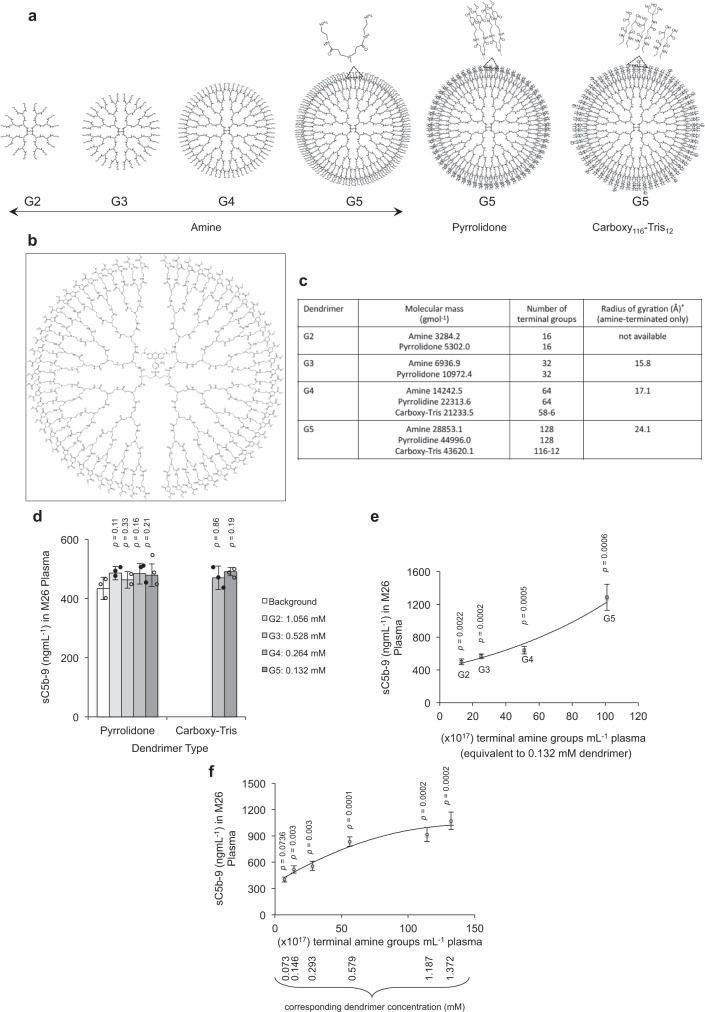
Dendrimer characteristics and the role of dendrimer end-terminal functionality in complement activation. **a** Structural representation of G2–G5 dendrimers with magnified views of the highlighted end-terminal region (dashed triangles). At physiological pH the end-terminal primary amines and carboxylic acids are predominantly protonated and deprotonated, respectively. **b** Typical structure of a G4 PAMAM dendrimer with a precisely core positioned sulforhodamine B. **c** Selected properties of G2–G5 dendrimers. *The values for radius of gyration were adopted from a previous small-angle X-ray scattering study^26^ **d** Pyrrolidone- and carboxy-Tris-terminated dendrimers do not trigger complement activation in human plasma (plasma code, M26; a healthy individual Caucasian, male, 26 years old) as determined through measurements of sC5b-9. Complement activation is compared at an equivalent number of dendrimer terminal groups (101 × 10^17^ terminal groups per mL of plasma). **e** The effect of different generations (G2–G5) of amine-terminated dendrimers on generation of fluid-phase sC5b-9 in M26 plasma. The best coefficient of correlation (*R*^2^ = 0.965) is computationally defined by the equation *y* = 422.15e^0.0106*x*^. **f** The effect of G2 dendrimer concentration on sC5b-9 formation in M26 plasma. The best coefficient of correlation (*R*^2^ = 0.955) is computationally defined by a quadratic polynomial fit (*y* = −0.0319*x*^2^ + 0.2006*x* + 366.92). In **e** and **f**, mean background sC5b-9 levels were 367 ± 7.2 µgmL^−1^ and 361 ± 7.3 µgmL^−1^, respectively. In panel **d,** bars represent mean ± s.d. of three separate experiments and each dot indicates the mean of three technical replicates. In **e** and **f**, each point represents the mean ± s.d. of three separate experiments, and each experiment was done in triplicate samples. In **d**, **e** and **f**, *p* values (unpaired, two-sided) are compared with the respective background (control) incubation. Source data are available in Source data file.

Amine-terminated G0–G5 PAMAM dendrimers were synthesised by an established divergent methodology from a 1,4-diaminobutane core with iterative reaction cycles between methyl acrylate and ethylene diamine (Supplementary Methods)^23^. Pyrrolidone- and carboxy-Tris-terminated dendrimers were synthesised from the corresponding amine-terminated PAMAM dendrimers (Supplementary Methods)^24,25^. When necessary, G4 PAMAM dendrimers with a precisely core positioned single sulforhodamine B was used from a previously characterised batch^25^. All half and full generation dendrimers were characterised by ^1^H-NMR and ^13^C-NMR (Supplementary Figs. 1–20; Supplementary Table 1), Fourier transform infrared spectroscopy (Supplementary Figs. 21–23; Supplementary Table 1), UV/Vis and fluorescence spectroscopy (Supplementary Fig. 24; Supplementary Table 1), size exclusion chromatography-multi angular light scattering (Supplementary Figs. 25–27; Supplementary Table 1), and mass spectrometry (Supplementary Methods; Supplementary Table 1). Dendrimer yields are reported in Supplementary Table 1. The structure and relevant characteristics of dendrimers are summarised in Fig. 1a–c.

### Dendrimer end-terminal groups modulate complement sensing

By considering G differences in geometry and spatial organisation of end-terminal functional groups^13–15^, complement activation by G2–G5 dendrimers was compared either at concentrations representing an equivalent total number of end-terminal functional groups or at an equivalent dendrimer molar concentration. The results in Fig. 1d show that at an equivalent number of end-terminal functional group (101 × 10^17^ end-terminal groups per mL of plasma) neither pyrrolidone- nor carboxy-Tris-terminated dendrimers trigger complement activation as determined through measurements of the soluble membrane-attack complex sC5b-9 (an established fluid-phase marker of whole complement activation pathway)^4,5^. Thus, within the G2–G5 family of dendrimers, complement evasion is independent of dendrimer size, geometry and end-terminal functionality (pyrrolidone and carboxy-Tris).

In contrast to pyrrolidone- and carboxy-Tris-terminated dendrimers, amine-terminated (presumed to have a net cationic charge at neutral pH) dendrimers trigger complement activation. The results in Fig. 1e show exponential elevation of sC5b-9 above background in the order of G2 (least) to G5 (highest) dendrimers at an equivalent molar concentration. The extent of sC5b-9 elevation strongly correlates (*R*^2^ = 0.965) with the total number of primary amines (Fig. 1e), which is increased by a factor of two with each dendrimer generation (Fig. 1c). In line with this notion, increasing of G2 dendrimer concentration (and hence the number of terminal amines) also correlates (*R*^2^ = 0.955) with the concentration of fluid-phase sC5b-9 in the same plasma (Fig. 1f). Thus, the concentration of fluid-phase sC5b-9 at 1.372 mM G2 dendrimer closely approaches that of 0.132 mM G5 dendrimer. This translates to ~30% more G2-derived end-terminal amines in replicating the G5 dendrimer superiority in complement activation. Furthermore, among spherical dendrimers, the G5 species are three times more effective than that of G4 in activating complement (Fig. 1e). However, with both G4 and G5 a hypothetical surface area of ~0.57 nm^2^ is available per end-terminal amine occupancy (based on small-angle X-ray scattering-derived radius of gyration^26^ Fig. 1c). Thus the higher efficacy of G5 dendrimers in complement activation is a reflection of their superior polyvalency over G4 (and lower G) dendrimers^13^, and presumably augmented by its lower curvature.

### Complement evasion and inactivation

Here we examine likely events that could explain why pyrrolidone- and carboxy-Tris terminated dendrimers do not activate the human complement system. First, we show that the lack of complement activation by pyrrolidone- and carboxy-Tris-terminated dendrimers is not due to dendrimer-mediated inactivation/inhibition of the whole complement system, since the extent of whole complement activation (as a measure of fluid-phase sC5b-9 concentration) by two established complement activators (mannan-coated wells and zymosan) in dendrimer pretreated plasma remains comparable to that of untreated plasma (Supplementary Fig. 28).

Second, we tested whether dendrimers can modulate the function of C1q and MBL-MASPs, and affect the function of classical and lectin pathways. In both lectin and classical pathways, activation of the fourth complement protein (C4) is necessary to cleave the second complement protein (C2) and forming convertases that cleave C3^1,27^. C3 cleavage by classical and lectin pathway-derived C3 convertases, in turn, triggers formation of a new set of convertases that specifically cleave the fifth complement protein (C5). This eventually leads to an enzymatic assembly of the membrane-attack complex (C5b-9)^1,27^. In lectin pathway, activated MBL-associated serine proteases (MASPs) cleave C4, whereas in classical pathway this task is performed by activated C1s^27^. C4 cleavage liberates C4a and C4b fragments. In neat plasma, factor I through cofactor activity of C4-binding protein (C4bp) cleaves C4b yielding inactivated C4b (iC4b), which in turn, through further degradation by factor I (and with cooperation of C4bp) liberate C4c and C4d fragments (the final physiological degradation products of C4b)^27^. Thus, we pre-incubated C1q and MBL-MASPs with dendrimers prior to their addition to the C1q-depleted human serum and a human plasma genetically deficient in MBL, respectively, and monitored C4 cleavage in the presence of aggregated IgG (a classical pathway substrate)^4^ and mannan (a lectin pathway substrate)^4^. C4 cleavage was monitored with MicroVue C4d Fragment ELISA kit, which uses an anti-C4d specific monoclonal antibody that exclusively measures the amount of the C4d-containing activation fragments of C4 (C4b, iC4b and C4d)^4^. We show no manipulation of classical and lectin pathways of human complement on C1q and MBL-MASPs pretreatment with either pyrrolidone- or carboxy-Tris-terminated dendrimers (Supplementary Fig. 29). These observations are also in accord with the findings in Supplementary Fig. 28.

Next, we studied whether dendrimers can bind to complement pattern-recognition molecules, and chose C1q as an example. This was studied with a PAMAM dendrimer bearing a precisely core positioned single sulforhodamine B with 1:1 fluorophore to dendrimer stoichiometry^25^ (Fig. 1b). These dendrimers exhibit high fluorescent quantum yields, with fluorophore stability at both room temperature and 37 °C as well as across a range of pH values of physiological relevance^25^. We used fluorescent-labelled G4 pyrrolidone- and carboxylic acid-terminated dendrimers to estimate dendrimer binding to C1q. The use of G4 (instead of G5) dendrimers allows separation of unbound dendrimers from C1q by 30 kD molecular weight cut-off protein concentrators. Both dendrimer types poorly interact with C1q (0.16 ± 0.08 and 0.27 ± 0.06, mean ± s.d., for pyrrolidone- and carboxylic acid-terminated dendrimers per C1q molecule, respectively, at dendrimer:C1q mole ratio of 3:1; *n* = 3 replicates). These observations are in line with the results in Supplementary Figs. 28 and 29.

The complement control protein fH and fH-like protein 1 (fHL-1) are among the key fluid-phase regulators of the alternative pathway and the amplification loop, and function as cofactors for cleavage of C3b by factor I^27,28^. Accordingly, binding of pyrrolidone- and carboxylic acid-terminated dendrimers to fH/fHL-1 might contribute to complement evasion by these dendrimers. Therefore, we investigated the binding of sulforhodamine B-labelled G4 PAMAM dendrimers to human fH. The results in Supplementary Fig. 30a show neither pyrrolidone- nor carboxylic acid-terminated dendrimer favourably interact with fH. However, it is still possible that fH could bind to dendrimer-protein complexes. Albumin is the most abundant plasma protein, and previous studies have reported interaction between dendrimers and albumin^29,30^. Thus, we coated the wells of microtiter plates with human albumin and then incubated albumin-coated wells with fluorescent dendrimers. We estimated binding of 0.8 ± 0.4 (mean ± s.d.; *n* = 3 independent replicates and each run in triplicate samples) carboxylic acid-terminated dendrimer per albumin molecule, but with pyrrolidone-terminated dendrimers the binding was negligible (<0.15 ± 0.1, mean ± s.d.; *n* = 3 independent replicates and triplicate samples). Next, we studied fH binding to both native albumin and dendrimer (carboxylic acid-terminated)-bound albumin. The data in Supplementary Fig. 30b show comparable results in fH deposition on albumin and dendrimer-bound albumin. We therefore suggest that dendrimer binding to fH is unlikely to account for complement inactivation.

Finally, the neutrality of pyrrolidone-dendrimers towards complement is in agreement with the excellent immune safety record of polyvinylpyrrolidone, which was given as a plasma expander to over half-million human recipients over the years^31,32^.

### Complement activation reproducibility in plasma

**Fig. 2:**
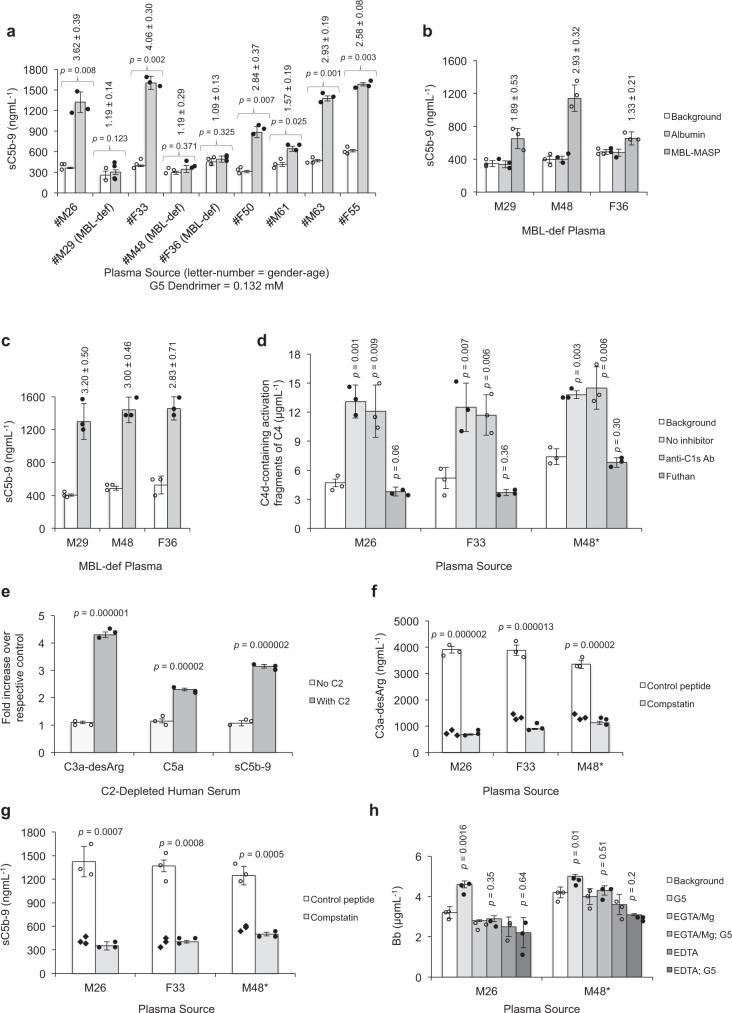
Amine-terminated PAMAM dendrimers trigger lectin pathway and amplification loop of the alternative pathway. **a** sC5b-9 formation in plasma of nine human subjects on exposure to G5 amine-terminated PAMAM dendrimers. Plasma source (letter–number) = (gender–age). Three individuals were genetically deficient in MBL (MBL-def) (Supplementary Table 2). Open columns = background levels of sC5b-9, and grey columns = sC5b-9 levels on dendrimer incubation. **b** Incubation of G5 amine-terminated PAMAM dendrimers with MBL-deficient plasma reconstituted with MBL-MASPs (equivalent to 3 µg MBL mL^−1^) elevates sC5b-9 levels. Human serum albumin was used as a negative control protein. **c** Mannan-coated wells induce fluid-phase sC5b-9 rises in MBL-def plasma on reconstitution with MBL-MASPs (equivalent to 3 µg MBL mL^−1^). Open columns = controls (MBL-def plasma with added albumin), and grey columns = reconstituted MBL-def plasma with MBL-MASPs. In **a**–**c**, vertical numbers denote folds increases over their respective background. **d** The effect of anti-C1s monoclonal antibody (100 µgmL^−1^) and futhan (150 µgmL^−1^) on G5 amine-terminated dendrimer-mediated complement activation in plasma of two healthy individuals (M26 and F33) and a plasma from a subject with MBL deficiency (M48) on reconstitution with MBL-MASPs (denoted as M48*) (equivalent to 3 µg MBL mL^−1^). **e** G5 amine-terminated PAMAM dendrimer-mediated complement activation in a commercially obtained C2-depleted human serum is restored on C2 (30 µgmL^−1^) addition. **f** C3-dependent complement activation by G5 amine-terminated PAMAM dendrimers as a measure of C3a-desArg. **g** C3-dependent complement activation by G5 amine-terminated PAMAM dendrimers as a measure of sC5b-9. In **f** and **g**, compstatin concentration was 40 µM. **h** G5 amine-terminated PAMAM dendrimers promote alternative pathway turnover (as a measure of Bb rises over background) through the amplification loop. In all panels, the dendrimer concentration is 0.132 mM. In all panels, bars show mean values ± s.d. of three separate experiments, and each dot indicates the mean of three technical replicates. In **f** and **g**, each black rhombus dots in open columns represent the mean of three technical replicates of plasma background levels of C3a-desArg and sC5b-9, respectively. All *p* values (unpaired, two-sided) are compared with their respective controls. Source data are available in Source data file.

Considering between-subject differences in the levels of complement proteins and nanomedicine-mediated complement activation pathways^1^, we further monitored the reproducibility of dendrimer-mediated complement responses in plasma of eight additional human subjects. Three donors were identified as genetically deficient in MBL (MBL levels <120 µgmL^−1^)^4^ (Supplementary Table 2). All other plasma samples were functional in all three pathways of the complement system as confirmed with WIESLAB qualitative Complement System Screen kit^4^ (herein as ‘complement-competent’ plasma). The results in Fig. 2a show variable sC5b-9 folds increase in ‘complement-competent’ individuals on treatment with amine-terminated G5 dendrimers. On the other hand, cohorts genetically deficient in MBL show no dendrimer-mediated sC5b-9 rises over their corresponding background levels. These differences among this small group of cohorts not only establish inter-individual variability in complement activation, but also delineate a predominant role for MBL and the lectin pathway in G5 dendrimer-mediated complement activation. These observations also indicate that, at least, in MBL-deficient plasma samples dendrimers do not directly trigger activation of the alternative pathway. This is consistent with the expected protonated state of the bulk of terminal amine groups at physiological pH^33^ (G5 amine-terminated dendrimer zeta potential = +39.3 ±  2.1 mV, mean ± s.d., in McIlvaine buffer, pH 7.0), which further minimises the possibility of nucleophilic attack towards the internal thioester bond in the α-chain of nascent C3b molecules (the proteolytically activated forms of C3)^5^.

Next, the role of lectin pathway in amine-terminated dendrimer-mediated complement activation was confirmed by addition of MBL-MASPs to MBL-deficient plasma samples, which restored complement activation by dendrimers (Fig. 2b) and the positive control mannan-coated plates (Fig. 2c). However, in all MBL-deficient plasma samples, the addition of MBL-MASPs generated comparable folds increase in sC5b-9 on mannan challenge, but not with dendrimers. These variations may be related either to a rate-limiting factor within the initiation phase of the lectin pathway (and presumably controlled by the fraction of protonated terminal amines) or to differences in the efficacy of the amplification loop of the alternative pathway on lectin pathway activation (this is studied below). In addition to the above, lack of complement activation by pyrrolidone- and carboxy-Tris-terminated dendrimers was also reproducible in other tested ‘complement-competent’ plasma samples (Supplementary Fig. 31).

### Amine-terminated dendrimers do not activate MBL-MASPs

First, we investigated the ability of amine-terminated dendrimers in activating MBL-MASPs in a reconstituted upstream lectin pathway. The results in Supplementary Fig. 32 show the inability of amine-terminated G2–G5 dendrimers to liberate C4d-containing activation fragments of C4 in the reconstituted upstream lectin pathway. Thus, amine-terminated dendrimers neither activate MASPs to cleave C4, nor cleave C4 directly. Contrary to these observations, we detected C4d-containing activation fragments of C4 in ‘complement-competent’ plasma and in MBL-deficient M48 plasma on reconstitution with MBL-MASP (denoted as M48*) by amine-terminated dendrimers (Fig. 2d). To further confirm that C4d-containing fragments are solely liberated on lectin pathway activation, plasma samples were pre-incubated with anti-C1s antibodies prior to dendrimer addition. These antibodies react with both active and inactive C1s and recognise the binding site of C1s for C4^5^. The results in Fig. 2d show no significant decline in the level of dendrimer-mediated C4d-containing fragments in the presence of anti-C1s antibodies. This excludes the role of classical pathway in complement activation. In contrast to anti-C1s antibody treatment, the broad-spectrum serine protease inhibitor futhan, which inhibits complement activation through all three pathways^5,34^, inhibited liberation of C4d-containing fragments on dendrimer challenge (Fig. 2d). Thus, liberation of C4d-containing fragments in dendrimer-treated plasma is serine protease-dependent.

Next, we established that lectin pathway-derived C3 convertases (and not dendrimers) are directly responsible for C3 cleavage. This was shown in a commercially available C2-depleted human serum where dendrimer-mediated elevation of C3a-desArg, C5a and sC5b-9 only occur on C2 restoration (Fig. 2e). The C3-dependency of terminal pathway activation was further confirmed with compstatin (an established C3 inhibitor)^35,36^ (Fig. 2f and g). Thus, these observations further support the notion that direct nucleophilic attack by any possible unprotonated peripheral amines of dendrimers on nascent C3b or dendrimer-mediated conformational changes in C3/C3(H_2_O) is unlikely to trigger terminal pathway activation.

Lectin-pathway activation could enhance alternative pathway turnover through amplification loop^1,27^. Thus, by assessing factor B cleavage, one can estimate the extent of alternative pathway utilisation when calcium-sensitive pathways are intentionally inhibited^4,5^. Factor B forms a magnesium-dependent complex with C3b or C3(H_2_O)^27^. The resulting C3bBb is the C3 convertase enzyme of the alternative pathway, which can be stabilised by Factor P^27^. These convertases can be dissociated by spontaneous decay dissociation, or by fH or complement receptor 1. Accordingly, we measured dendrimer-mediated rises in Bb levels both in the absence and presence of EGTA/Mg^2+^. The results in Fig. 2h show dendrimers do not elevate plasma Bb levels in the presence of EGTA/Mg^2+^ (i.e., where calcium-sensitive pathways are inhibited^4,5^). Thus, the amplification loop is responsible for Bb elevation and causes exponential amplification of the complement cascade initiated by the lectin pathway. As an additional control, Bb elevation is totally abolished by EDTA (an inhibitor of all three known pathways of the complement system)^6^ (Fig. 2h).

While the collective data in Fig. 2 confirm that amine-terminated dendrimer treatment of plasma lead to MBL-MASPs initiation of the whole complement system, it is still not clear how MBL-MASPs have themselves become activated, since dendrimers themselves do not modulate the function of MBL/MASPs (Supplementary Fig. 32). We speculate that MBL-MASPs activation is through an intermediary molecule, which in turn has become functional through dendrimer action/binding. This is investigated below.

### Natural IgMs as intermediary molecules in lectin pathway activation

IgM and IgG are among the most abundant Igs and some of their glycosylated variants are known to trigger lectin pathway and induce complement activation in vitro and in vivo^21,37,38^. The heavy chain of IgM bears oligosaccharides at five asparagine (Asn) residues, where two of these residues (Asn-402 and Asn-563) are occupied by oligomannose glycans that serve as target for MBL^21^. This glycosylated subset of plasma IgM accounts for ~20% of the total human IgM pool, but pentameric IgM bound to antigen cannot interact with MBL due to the presence of the J chain, whereas hexameric IgM lacks the J chain and exhibits a planar arrangement that allows for antigen and MBL binding^21^. MBL also binds to an agalactosyl subset of IgG, which accounts for ~35% of normal plasma IgG, where the single Asn-297 *N*-linked site in the γ chain is occupied by terminal N-acetyl-glucosamine residues^38^.

Earlier proteomic studies indicated association of amine-terminated G4–G7 PAMAM dendrimers with Ig μ chain (corresponding to IgM) and heavy constant γ-1 chain in human plasma^7^. Furthermore, amine-, pyrrolidone- and carboxylic acid-terminated PAMAM dendrimers, have all been shown to alter the secondary structure and conformation of γ-globulins^39^. Therefore, we hypothesise that interactions between dendrimers and MBL-binding Ig glycoforms in a functional motif-driven manner might trigger complement activation by enhancing MBL binding to complex oligomannose glycans in IgM (and/or IgG).

**Fig. 3:**
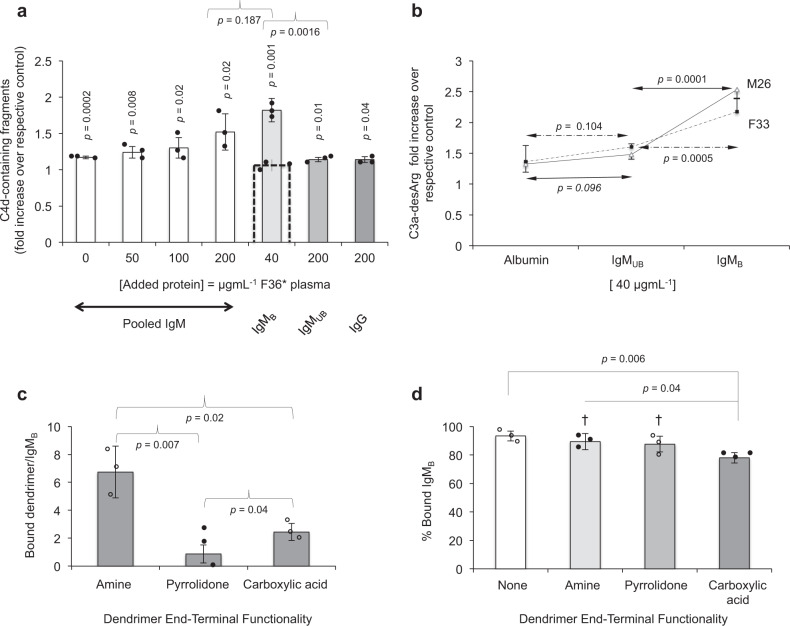
Natural IgM molecules bind to dendrimers and trigger complement activation through the lectin pathway. **a** Increasing concentrations of human IgM in F36* plasma (F36 plasma reconstituted with MBL-MASPs; equivalent to 3 µg MBL mL^−1^) elevates levels of C4d-containing activation fragments of C4 in the presence of amine-terminated G5 dendrimers. IgM_B_ = IgM glycosylated variants that bind to MBL, and IgM_UB_ = IgM variants that are not retained by agarose-MBL. Liberation of C4d-containing activation fragments of C4 is inhibited by 10 mM EGTA/2.5 mM Mg^2+^ (the broken-line overlay column). Bars represent mean values ± s.d. of three technical replicates, and each in triplicate samples. The *p* values (unpaired, two-sided) represent comparison of each treatment with their corresponding controls (plasma incubations without dendrimers, but in the presence of all other additives); otherwise indicated. **b** Complement activation (as a measure of C3a-desArg elevation) in two IgM-depleted plasma samples is restored on addition of IgM_B_ (40 µgmL^−1^). The points are mean of three separate experiments ± s.d. and each experiment was done in triplicate samples. Differences between groups were examined using ANOVA followed by multiple comparisons with Student–Newmann–Keul test. In **a** and **b**, the dendrimer concentration is 0.132 mM. **c** Estimation of dendrimer binding to IgM_B_. **d** Binding of dendrimer-IgM_B_ complexes to agarose-MBL. In panels **c** and **d**, bars show mean values ± s.d. of three technical replicates, and each in triplicate samples. ^†^*p* > 0.2, compared with “no dendrimer” (IgM_B_ only). Source data are available in Source data file.

To test this hypothesis first we reverted to the MBL-deficient F36 plasma. Indeed, among the MBL-deficient plasma samples, dendrimer-mediated complement activation was relatively inefficient in MBL-MASP reconstituted F36 plasma (herein as F36*) (Fig. 2a). This might have been related to low titre of MBL-binding Ig glycoforms in this plasma. The results in Fig. 3a show significant dendrimer-mediated folds increases in C4d-containing fragments of C4 in F36* plasma on addition of human IgM (from a pooled source) at concentrations ≥100 µgmL^−1^. Next, from a pooled IgM source we separated MBL-binding IgM variants (denoted as IgM_B_) using a commercial MBL-resin as described previously^21^. Addition of IgM_B_ to F36* significantly elevate the level of C4d-containing fragments on dendrimer challenge in a calcium-dependent manner, which support lectin pathway activation. On the other hand, MBL unbound IgM fraction (IgM_UB_) exert some effects at high concentrations (*p* = 0.01), whereas on IgG addition rises in C4d-containing fragments is even less significant (*p* = 0.04). Next, we studied the role of IgM in two ‘complement-competent’ plasma samples on partial IgM depletion. Again, addition of 40 µgmL^−1^ IgM_B_ to partially IgM-depleted plasma samples significantly elevate C3a-desArg levels on dendrimer challenge compared with human albumin (a non-specific protein control) and IgM_UB_ (Fig. 3b). Collectively, these observations indicate a role for IgM_B_ subsets in complement activation in the presence of dendrimers.

Two sets of experiments were then conducted to gain more information on dendrimer-IgM subset interaction. First, sulforhodamine B-labelled G4 PAMAM dendrimers^25^ were used to estimate the number of bound dendrimers per IgM_B_ molecule. The data in Fig. 3c further shows highest association of dendrimers with IgM_B_ in the order of amine > carboxylic acid > pyrrolidone functionality. Second, depending on dendrimer type, IgM_B_ retention in agarose-MBL is affected. With amine- and pyrrolidone-terminated dendrimers (dendrimer:IgM mole ratio = 10:1) ~90% of IgM_B_ is retained, which is not significantly different (*p* > 0.2, unpaired, two-sided) from IgM_B_ binding alone (Fig. 3d). However, carboxylic acid-terminated dendrimers marginally (but still significantly), decreases IgM_B_ binding to agarose-MBL (Fig. 3d). On the basis of these binding differences, we propose a complement activation scheme below.

### A proposed scheme in complement activation

**Fig. 4:**
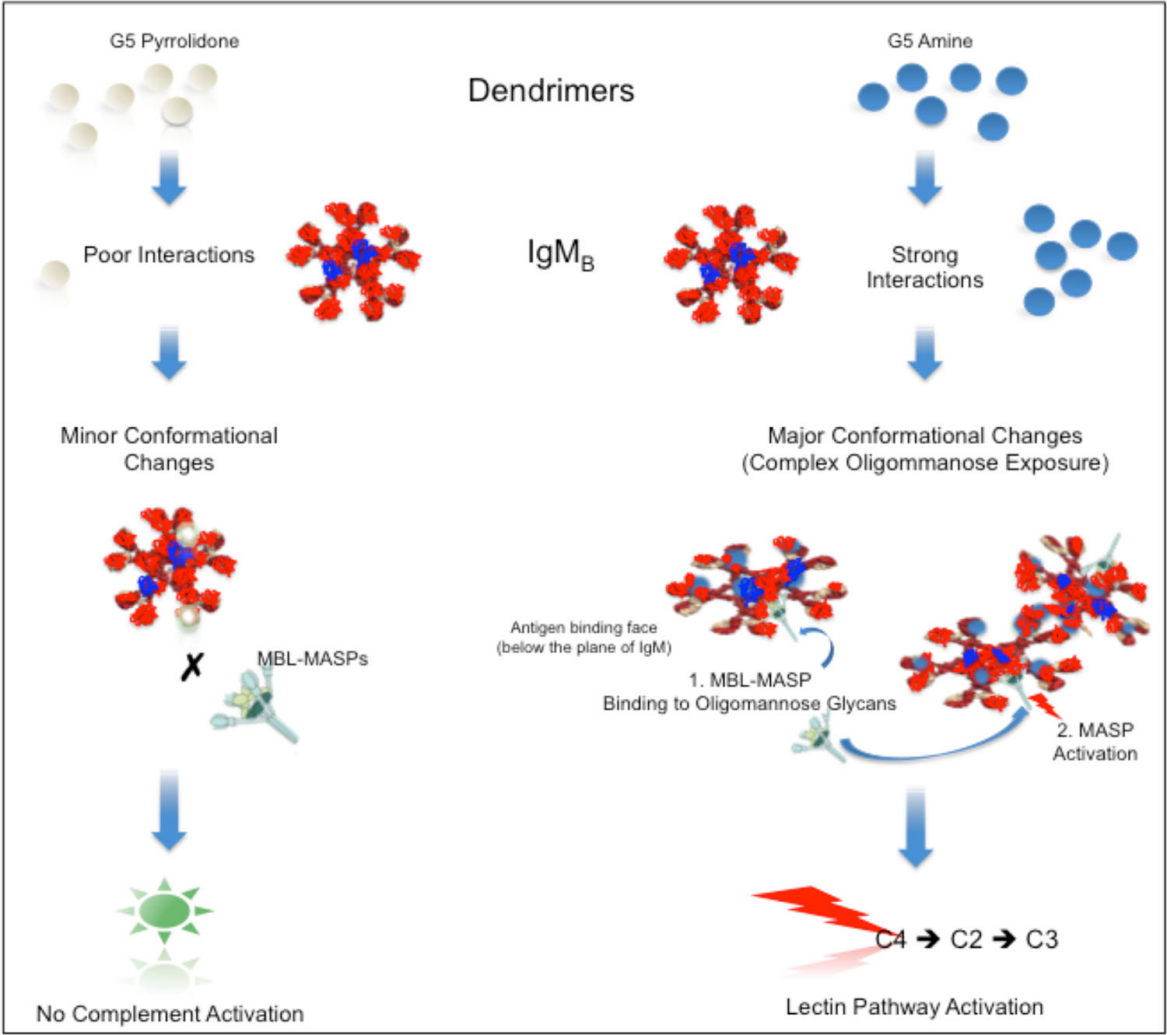
A proposed scheme for activation of the lectin pathway by dendrimer-IgM_B_ complexes. G5 dendrimers were chosen as example. Pyrrolidone-terminated dendrimers poorly bind to IgM_B_ and on binding do not induce significant conformational changes in the immunoglobulin structure to promote MBL-MASPs binding. Amine-terminated dendrimers bind to IgM in numbers (and might also promote complex formation with other plasma proteins). This induces conformational changes in IgM and exposes complex oligomannose glycans on the antigen-binding face of the immunoglobulin, thereby allowing MBL binding to complex oligomannose glycans. On MBL binding, MBL activates its MBL-associated serine proteases (MASPs), which, in turn, cleave C4 and C2 to form the lectin pathway C3 convertase.

Amine-terminated dendrimer hitchhiking on IgM_B_ triggers activation of the lectin pathway by IgM_B_. Thus, binding of amine-terminated dendrimers to IgM_B_ potentially induces necessary conformational changes in the Ig structure that promotes MBL-MASPs binding to complex glycans on the antigen-binding face of the molecule, which subsequently triggers MASP activation (Fig. 4). This process is more efficient with spherical dendrimers (G4 and G5) due to their higher multivalency and larger size compared with “star-like” G3 and G2 dendrimers. Thus, with lower G dendrimers (G3 and G2) more dendrimers must bind to IgM_B_ to induce necessary conformational changes to allow MBL binding. This might explain observations in Fig. 1f. Although the exact dendrimer-binding domains on IgM_B_ remain to be identified, dendrimers could either induce conformational changes on binding to non-antigen-binding face of the Ig or on binding to antigen-binding face might act cooperatively with the complex oligomannose glycans to enhance MBL-MASP docking. Amine-terminated dendrimers also bind to other plasma proteins such as albumin, fibrinogen, and apo-lipoprotein B-100^7,22,29,30,40,41^. Thus, G4 and G5 dendrimers due to their superior polyvalency and larger size than sub-G3 dendrimers might further promote the formation of large complement-activating IgM_B_-protein (and lipoprotein) complexes (Fig. 4). This is an important consideration, since in plasma only a small fraction of the dendrimers is expected to interact with IgM_B_.

In contrast to amine-terminated dendrimers, only a few pyrrolidone- and carboxylic acid-terminated dendrimers bind to IgM_B_ (Fig. 3e). The result with pyrrolidone functionality is in agreement with an earlier study showing low affinity of pyrrolidone dendrimers for other proteins, and where dendrimer binding had no significant effect on protein conformation^24^. Thus, dendrimer-induced IgM_B_ conformational changes are apparently minor to activate lectin pathway on MBL binding (Fig. 4). Carboxylic acid-terminated dendrimers, however, interfered with the binding of a small fraction of IgM_B_ to MBL. This may suggest that with some IgM_B_ molecules, carboxylic acid-terminated dendrimers stochastically occupy domains in the antigen-binding face of IgM_B_ and sterically interfere with MBL binding.

Considering limitations of some employed techniques used in this study (e.g., fluorescent measurements), future approaches with integrated biophysical modalities such as isothermal microcalorimetry, quartz crystal microbalance, second-harmonic generation, chemical force microscopy, and static and dynamic molecular simulations is still necessary to further verify modes of interactions between dendrimers (including an assessment of the role of G-dependent degree of dendrimer interpenetration), Igs and complement pattern-recognition molecules.

### Complement evasion by drug-incorporated dendrimers

**Fig. 5:**
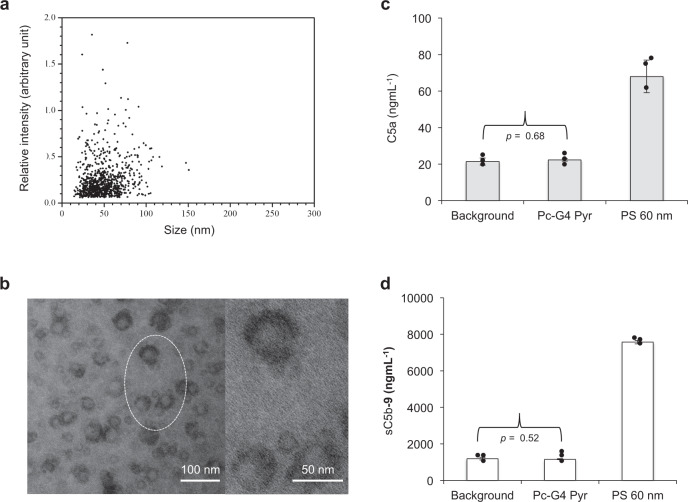
Characteristics of Pc-G4 Pyr dendrimer complexes and their complement responses. **a** A typical NTA 2D plot of relative light scattering intensity of Pc-G4 Pyr particles versus the estimate of the nanoparticle size. The experiment was repeated three times with identical distribution profile. A hydrodynamic size of 46 ± 3 nm (mean ± s.d.) was calculated from three independent determinations. **b** A representative transmission electron micrograph of Pc-G4 Pyr dendrimer complexes (left), and magnified view of the highlighted region (right). The experiment was repeated three times with identical results. **c**, **d** Lack of complement activation by Pc-G4 Pyr dendrimer complexes (3.5 mgmL^−1^) in a typical human plasma determined through measurements of fluid-phase C5a and sC5b-9. A commercially available sulfated polystyrene nanoparticle suspension (PS) of 62 ± 6 nm (polydispersity index = 0.03 as determined by laser light scattering) was used as positive control for complement activation (final nanoparticle concentration = 3.5 mgmL^−1^). In **c**, **d**, each bar represents the mean ± s.d. of three separate experiments, and each dot indicates the mean of three technical replicates. Indicated *p* values are derived from unpaired two-sided student *t*-test. Source data are available in Source data file.

G3–G5 PAMAM dendrimers have widely been used to enhance solubilisation of poorly soluble drugs by partially accommodating them in their void spaces^42–44^. Alternatively, guest molecules can be attached covalently to the reactive dendrimer end-groups. However, it has been shown that covalent and non-covalent drug association (and particularly bulky hydrophobic molecules) with dendrimer could yield complexes with variable sizes^11,43,44^. Thus, contrary to single-molecule pyrrolidone- and carboxy-Tris-terminated dendrimers, some drug-dendrimer complexes, depending on their size, morphology and surface properties, could trigger complement activation through non-specific plasma biomolecule deposition^6^. To investigate these possibilities, we chose phthalocyanine (Pc, a large aromatic macrocyclic compound) as a candidate molecule for incorporation into the complement-evading G4 pyrrolidone- and carboxy-Tris-terminated dendrimers. First, we covalently attached Pc molecules to the dendrimer exterior. This yielded two and three molecules of Pc attached per pyrrolidone- and carboxy-Tris-terminated dendrimers, respectively (Supplementary Tables 1, 3). The distribution of Pc molecules on the dendrimer surface is statistical, but this promoted the formation of complexes (herein as Pc-G4 Pyr or Pc-G4 Carboxy-Tris) with a range of hydrodynamic diameters exceeding the native dendrimer size as shown by Nanoparticle Tracking Analysis (Fig. 5a) and transmission electron microscopy (Fig. 5b). The extensive network of π electrons in Pc, presumably promote the formation of such complexes through π–π interaction. Notwithstanding, both Pc-G4 Pyr and Pc-G4 Carboxy-Tris complexes fully evaded complement activation (Fig. 5c, d; Supplementary Table 3). Next, we tested non-covalent entrapment of Pc in dendrimers and the ability of the resultant formulation in triggering complement activation. While dendrimers could solubilise Pc and form large complexes, no complement responses were observed (Supplementary Table 3).

## Discussion

This study has shown dendrimer end-terminal functionality can modulate complement responses. Complement pattern-recognition molecules have long been suggested to recognise patterned motifs in 2–15 nm ranges apart^17–20^. In dendrimers the end-terminal motifs are spaced <1 nm from each other, which might explain why dendrimers overcome sensing by complement pattern-recognition molecules such as C1q and MBL, and bypass complement activation through classical and lectin pathways. We cannot disregard some non-specific binding of dendrimers to C1q and MBL, but experiments described here ruled out complement activation processes through possible dendrimer interaction with these complement pattern-recognition molecules. Our results also show that dendrimers do not trigger the alternative pathway. While, pyrrolidone- and carboxy-Tris-terminated dendrimers evade complement response, and independent of fH binding, amine-terminated dendrimers indirectly trigger complement activation. The latter observation was a consequence of dendrimer interaction with Igs. More specifically, amine-terminated dendrimer hitchhiking on a subset of natural MBL-binding IgM led to antibody-mediated activation of the lectin pathway. Experiments with reconstituted lectin pathway further support this statement, since in the presence of amine-terminated dendrimers C4 cleavage did not occur.

Intravenously injected dendrimers passively accumulate at pathological sites where the barrier function of vasculature is compromised (e.g., selected tumours, and inflammatory regions)^11^. Accordingly, we propose pyrrolidone- and carboxylic acid (including carboxy-Tris)-terminated dendrimers for the development of therapeutic intervention of inflammatory conditions and pathologies where dysregulated local complement activation could be problematic (e.g., solid tumours, rheumatoid arthritis and atherosclerotic plaques)^1,45–48^. For instance, intratumoural complement activation by extravasated complement-activating nanoparticles was shown to promote tumour progression^49^, and in atherosclerosis anaphylatoxin liberation and formation of the terminal complement complex is suggested to be proatherogenic leading to plaque destabilisation and rupture^47,48^. Such dendrimers may even provide opportunities for the delivery of complement inhibitors^1,50^, by modulating their pharmacokinetics and tissue distribution. Furthermore, surfaces of appropriate organic and inorganic nanoparticles might be amenable to coating with pyrrolidone- and carboxylic acid/carboxy-Tris functionalised dendrimers (or their respective dendrons). This approach could lead to the development of a broader spectrum of complement-evading multifunctional nanomedicines and nanotheranostics. These dendrimers might also prove useful in vaccine stabilization and overcoming anaphylactoid reactions seen with PEGylated lipid nanoparticles (e.g., as in COVID-19 vaccines), where a role for anti-PEG antibodies has been speculated^51^. From a broader perspective, we also envisage dendrimeric (e.g., pyrrolidone-terminated) coating of drug-eluting stent surfaces, contact lenses and (haemo)dialysis membrane filters to minimise protein binding and complement activation.

Finally, our observations might project unrecognized strategies by which microbial pathogens evade complement sensing. Some virulent pathogens have adopted surface strategies based on sialylated glycans and polysialic acids to attract fH/fHL-1 and bypass complement activation^52^. Factor H, through its CCP20 domain bind to the glycerol side chain and the carboxyl moiety of sialic acid^53^, but fH binding is still dependent on the number of available and accessible binding sites, which is modulated by mobility and dynamics of surface projected polysialic acid chains. Motivated by the fH-independent complement escape of carboxylic acid/carboxy-Tris-terminated dendrimers, it is tempting to speculate that virulent pathogens might simultaneously display conformational clouds of carboxyl and hydroxyl motifs in Angstrom-scale spacing arrangement to escape complement attack independent of fH-directed molecular crypsis.

## Methods

### Reagents

Zymosan from *Saccharomyces cerevisiae* (Z4250), mannan (M3640), human IgM (18260, MFCD00163928) (0.05 M Tris-HCl, 0.2 M NaCl, 15 mM sodium azide, pH 8.0), human IgG (12511, MFCD00163923) (0.01 M sodium phosphate, 0.15 M NaCl, 15 mM sodium azide, pH 7.4), goat anti-mouse IgG antibody [(H+L) HRP conjugate] (AP308P), albumin from human serum (A1653), and HiTrap^®^ Protein G High Performance Columns (GE17-0404-01), and Nunc-Immuno™ Microwell™ 96-well solid plates (M9410) were from Sigma-Aldrich (Merck KGaA, Damstadt, Germany). Lepirudin (Refluden) was from Hoechst and Futhan (nafamostat mesylate) (SKU F-1160) was from AG Scientific. Mouse (IgG1) anti-human C1s monoclonal antibody (HM2108-500 UG, clone M81), human complement factor H ELISA kit, and human MBL-assay kit (HK323-02) were obtained from Hycult Biotech (Uden, The Netherlands). Complement proteins C1q (A400), C2 (A427), C4 (A402), Factor H (A410), Factor I (A411), C1q-depleted human serum (A509), C2-depleted human serum (A500) and MicoVue enzyme immunoassay kits for C4d (A008), C3a-desarg (A032), sC5b-9 (A029), C5a (A025) and Bb (A027) were from Quidel (CA, USA). Recombinant human C4-binding protein (ab130028) and mouse monoclonal [C18/3] anti-factor H antibody (ab121055) were from Abcam (UK). TMB (3,3′,5,5′-tetramethylbenzidine) and Human IgM ELISA kit (BMS2098) was from LifeTechnologies Ltd. (Paisley, UK). Albumin human ELISA kit (EHALB), Pierce^TM^ IgM Purification Kit (44897) and Pierce^TM^ Protein Concentrators (10 K and 30 K MWCO) were from ThermoFisher Scientific (Loughborough, UK). WIESLAB qualitative Complement System Screen kit (COMPL300) was from Svar (Malmo, Sweden). Compstatin and the compstatin control peptide were a kind gift from Prof. Tom E. Mollnes (Oslo University Hospital, Norway). Sulfated polystyrene nanospheres were from Polysciences Europe GmbH (Germany).

### Dendrimer synthesis and characterisation

Synthetic procedures and characterization steps for all native dendrimers and G4 PAMAM dendrimers with either covalently attached or non-covalently incorporated phthalocyanine (Pc) are presented in the Supplementary Methods. G4 PAMAM dendrimers with pyrrolidone, carboxylic acid and amine functionality bearing a precisely core positioned single sulforhodamine B were used from a characterised batch^25^.

### Complement activation

Blood samples were collected from volunteered human subjects with informed consent according to the Danish law of blood donation with general approval at Copenhagen University. Blood was collected into blood tubes containing lepirudin as an anticoagulant. This anticoagulant does not affect complement system functionality^6^. The blood was centrifuged at 5000 × *g* for 20 min to obtain plasma. Plasma was aliquoted in Eppendorf tubes and stored at −80 °C. For complement activation studies plasma samples were thawed at room temperature and then brought to 37 °C in a water bath prior to complement activation studies. Repeated freeze-thawing was avoided and never exceeded twice. Purified MBL/MASPs from pooled human serum was obtained as described elsewhere^4,54^. Hycult Biotech (Uden, The Netherlands) human MBL ELISA assay kit was used for determination of MBL levels in plasma and in purified MBL-MASPs following manufacturer’s instruction. The qualitative activity of all three-complement pathways in plasma samples was screened with the WIESLAB qualitative Complement System Screen kit in accordance with the kit’s instruction^4^.

For measurement of C4d-containing activation fragments of C4 (C4b, iC4b and C4d), C3a-desArg, Bb, C5a and sC5b-9, the reaction was started by adding the required quantities of dendrimers (in sterile 10 mM phosphate buffer, pH 7.0) to undiluted human plasma samples or to the C2 (or C1q)-depleted human serum in Eppendorf tubes. Typical dendrimer:plasma volume was 1:4. Samples were incubated in a shaking water bath at 37 °C for 30 min. Reactions were terminated by addition of “sample diluent” from the kits. Alternatively, physiological saline containing 25 mM EDTA (100 µL) was used to terminate reactions. Dendrimer-induced rises of plasma (or serum) complement activation products were measured in respective MicroVue ELISA kits by following manufacturer’s protocol^4,5^. Standard curves were constructed with standard complement activation products supplied in the assay kits. In some experiments, other additive were present to delineate enzymatic processes and pathways of complement activation by dendrimers. The additives included anti-C1s antibody (100 µgmL^−1^)^5^, futhan (150 µgmL^−1^)^5^, MBL/MASP (MBL = 3.0 µgmL^−1^ plasma)^4^, compstatin and its control peptide (40 µM)^36^, EGTA/Mg^2+^ (10 mM/2.5 mM)^4,6^, EDTA (10 mM)^6^, C2 (30 µgmL^−1^), human IgM (40–200 µgmL^−1^) and human IgG (200 µgmL^−1^). These additives were added to human plasma or serum and incubated for 5 min at 37 °C prior to dendrimer addition. Control plasma (or serum) incubations contained physiological saline (the same volume as dendrimers and other additions) to account for the background levels of complement activation products throughout the assay procedures. Zymosan (250 µgmL^−1^ serum) was used as positive control to monitor complement activation in plasma and sera^4^. In some experiments, complement activation by Pc-G4 dendrimer complexes (3.5 mgmL^−1^) was studied. Prior to ELISA measurements, samples were centrifuged in a benchtop Microfuge at 12,000 × *g* for 15 min and the supernatant was used for ELISA determination of the fluid-phase sC5b-9 and C5a.

For specific experiments, Nunc-Immuno^TM^ Microwell™ 96-well solid plates were coated either with 200 µL of mannan (500 µgmL^−1^ in 15 mM sodium carbonate pH 9.5) or different generation of amine-terminated dendrimers (750 µgmL^−1^ in 10 mM phosphate buffer, pH 7.0) at 4 °C overnight. After incubation, the plates were washed twice with 10 mM phosphate buffer, pH 7.0. Immediately after washing, coated plates were treated with either undiluted human plasma (40 µL) or reconstituted upstream lectin pathway. Reconstituted upstream lectin pathway contained MBL/MASPs = 5 µgmL^−1^ MBL, C2 = 30 µgmL^−1^, C4 = 650 µgmL^−1^, C4-binding protein = 200 µgmL^−1^ and complement factor I = 35 µgmL^−1^. The final volume was adjusted to 100 µL. After 30 min of incubation at 37 °C the reactions were stopped by the addition of 100 µL of physiological saline containing 25 mM EDTA. This was followed by ELISA measurement of fluid-phase complement activation fragments.

Mannan-coated wells were also used to assess complement functionality of MBL-deficient human plasma (40 µL) before and after addition of purified MBL-MASPs (equivalent to 3 µg MBL mL^−1^) in a final volume of 100 µL. After 30 min of incubation at 37 °C reactions were stopped by the addition of 100 µL of physiological saline containing 25 mM EDTA, and the levels of fluid-phase sC5b-9 were determined by ELISA.

### IgM treatment and dendrimer binding

Prior to use, human IgM was buffer exchanged with Tris-buffered saline (10 mM Tris-HCl, 150 mM NaCl, and 0.5 mM EDTA, pH 7.2). To account for possible IgG contaminants, IgM samples were passed through HiTrap^®^ protein G column. Collected IgM was buffer exchanged with 10 mM HEPES, 150 mM NaCl, 10 mM CaCl_2_ and 10 mM MgCl_2_, pH 7.4 (working buffer), then concentrated using Pierce^TM^ Protein Concentrators (30 K MWCO) on centrifugation (swinging bucket rotor at 5000 × *g* for 30 min at 22 °C). For IgM quantification a human IgM ELISA kit was used.

In some experiments, IgM was passed through Pierce^TM^ IgM Purification Column (rabbit MBL conjugated to agarose beads). This column has affinity for human IgM glycoforms that bind MBL^21^. The column was prewashed with a small volume (10 mL) of Tris buffer at 21 °C and then equilibrated with 20 mL of Tris-binding buffer (containing CaCl_2_, supplied by the manufacturer). IgM in Tris-binding buffer was applied to the column at 4 °C and left for 30 min at the same temperature. Afterwards, the column was washed with 40–50 mL of Tris-binding buffer. Bound protein was eluted at room temperature with elution buffer (provided by the manufacturer). Collected IgM was buffer exchanged with the working buffer and concentrated using Pierce^TM^ Protein Concentrators (30 K, MWCO) for complement activation studies. IgM was quantified with the human IgM ELISA kit.

To deplete plasma from IgM glycoforms that bind MBL, 0.5 mL of agarose-MBL was placed in Eppendorf tubes and washed three times each with 0.7 mL ice-cold Tris-binding buffer. After each washing step, tubes were centrifuged at 2800 × *g* for 1 min and the supernatant was removed from the slurry. Freshly thawed plasma (150 µL) was mixed with an equal volume of ice-cold Tris-binding buffer (without sodium azide) and added to the slurry. The tubes were gently agitated for 30 min at 4 °C. Following the incubation period, tubes were centrifuged at 2800 × *g* for 1 min. Supernatants were carefully removed and incubated twice more with a 0.5 mL washed ice-cold agarose-MBL. After the final run, plasma samples were used for complement activation studies with and without addition of IgM fractions obtained from the commercial IgM pool.

Dendrimer interaction with designated human blood proteins. Dendrimer interaction with IgM, C1q and human fH was followed with G4 dendrimers bearing a precisely core positioned single sulforhodamine B^25^. Dendrimers were incubated either with IgM glycoforms that bind to MBL (dendrimer to IgM mole ratio of up to 10:1) or C1q or fH (at different dendrimer to protein mole ratio) at 37 °C for 5 min. Unbound dendrimers were removed by centrifugation (swinging bucket rotor at 5000 × *g* for 30 min at 21 °C) using protein concentrators (30 K, MWCO). Retained protein-dendrimer complexes were washed twice with Tris-binding buffer. Control preparations were run in parallel (dendrimers alone and proteins alone). Dendrimer binding (average dendrimer molecule per protein molecule) was quantified from a dendrimer standard curve by measuring sample fluorescence (*λ*_abs,max_ = 520 nm, *λ*_em,max_ = 544 nm)^25^ and subtraction from respective controls. IgM and fH levels were quantified with respective ELISA kits following manufacturer’s protocols. C1q levels were quantified by protein assay. The binding of dendrimer-IgM complexes to MBL was assessed with 0.5 mL agarose-MBL.

In a different set of experiments, Nunc-Immuno^TM^ Microwell™ 96-well solid plates were coated with either 200 µL of human serum albumin (500 µgmL^−1^) overnight at 4 °C. After incubation, the plates were washed twice with 10 mM phosphate buffer, pH 7.0. Immediately after washing, some wells were incubated with fluorescent dendrimers (dendrimer to protein mole ratio, 5:1) for 1 h at 21 °C to assess dendrimer binding. Albumin levels were quantified with a commercially available ELISA kit. Unbound dendrimers were removed by washing the wells three times with 10 mM phosphate buffer, pH 7.0) and the amount of bound dendrimers was quantified by measuring sample fluorescent. Next, albumin and dendrimer-albumin-coated plates were treated with increasing concentrations of human fH and allowed to incubate for 60 min at 21 °C. Wells were washed with 10 mM phosphate buffer, and the presence of fH was assessed using mouse anti-fH IgG1 (1:400 dilution) followed by goat anti-mouse-HRP conjugated IgG (1:1000 dilution). Next, TMB substrate was added. Finally, the reactions were stopped with 1 N sulfuric acid and the plates were read at *λ* = 450 nm. Subtractions were made using plates without fH addition.

### Nanoparticle tracking analysis

NanoSight LM20 (Malvern, UK) was used for determination of size distribution and particle concentration of Pc-dendrimer complexes. Samples were diluted in Milli-Q water and measurements were performed at 21 °C. NanoSight NTA software (version 3.2) was used for data analysis. Each experiment was repeated three times.

### Zeta potential estimation

The zeta potential of dendrimers, where appropriate, was calculated from electrophoretic mobility measurements performed on a Zetasizer Nano ZS (Malvern, UK) in McIlvaine buffer (3.63 mM Na_2_HPO_4_, 0.18 mM citric acid and 0.13 mM KCl; pH 7.0) at 21 °C. Experiments were repeated five times and mean values ± s.d. is presented.

### Transmission electron microscopy (TEM)

A 10 μL of 0.05 mgmL^−1^ Pc-dendrimer complexes was placed onto a 200 mesh carbon-coated copper grids and allowed to stand for 5 min. Excess solvent was carefully removed by capillary action using a filter paper and the sample was immediately stained with 10 μL of 2% (v/v) phosphotungstic acid solution for 1 min. Excess stain was removed and the grids were allowed to dry for 30 min. Images were taken with a Philips CM100 transmission electron microscope (FEI/Philips, Eindhoven, Netherland) with an accelerating voltage of 80 kV. Image analyses were performed with Matlab 2014b (Mathworks, MA, USA).

### Statistical analyses

Complement activation studies were done in triplicate samples and each experiment was repeated three times. The efficacy of complement activation studies was established by comparison with baseline levels of complement fluid-phase products using two-sided unpaired *t*-test. Correlations between two variables were analysed by linear regression, and differences between groups (when indicated) were examined using ANOVA followed by multiple comparisons with Student–Newmann–Keul test^4^.

### Reporting summary

Further information on research design is available in the Nature Research Reporting Summary linked to this article.

## Supplementary information

Supplementary Information

Reporting Summary

## Data Availability

All original ^1^H-NMR and ^13^C-NMR spectra (Supplementary Figs. 1–20; Supplementary Table 1), Fourier transform infrared spectra (Supplementary Figs. 21–23; Supplementary Table 1), UV/Vis and fluorescence spectra (Supplementary Fig. 24; Supplementary Table 1), size exclusion chromatography-multi angular light scattering chromatogram (Supplementary Figs. 25–27; Supplementary Table 1), and mass spectra (Supplementary Methods; Supplementary Table 1) are reported in Supplementary Information file. Raw data for Fig. 1c is included in the Supplementary Table 1 in Supplementary Information file. No raw data is associated with Figs. 1a, b and 4. Remaining raw data supporting the findings of this study are available from the corresponding author upon reasonable request. Source data are provided with this paper.

## Source data

Source Data
