# Supplementary material for: Dendrimer end-terminal motif-dependent evasion of human complement and complement activation through IgM hitchhiking
Source: Nat Commun. 2021 Aug 11;12:4858. doi: 10.1038/s41467-021-24960-6 (PMC8357934; doi:10.1038/s41467-021-24960-6)
Supplement: Supplementary file 1 — Supplementary Information [file 41467_2021_24960_MOESM1_ESM.pdf]

Supplementary Information

Dendrimer end-terminal motif-dependent evasion of human complement and complement activation through IgM hitchhiking

Lin-Ping Wu, Mario Ficker, Jørn B. Christensen, Dmitri Simberg, Panagiotis N. Trohopoulos, Seyed M. Moghimi

Supplementary methods

General dendrimer synthesis and characterisation procedures. Unless otherwise stated, all starting materials were obtained from commercial suppliers and used as received. Solvents were HPLC grade and used as received. Methanol was dried over molecular sieves. Thin-layer chromatography was carried out using silica plates on aluminum (Merck, Silica 60-F_{254}, 0.2 mm layer thickness) with detection under UV light and, if required treatment with 1 % solution of ninhydrin in ethanol. UV/Vis spectra were recorded on a Perkin Elmer apparatus using 1 cm quartz cuvettes.

^{1}H-NMR and ^{13}C-NMR spectra were performed on a 300 MHz NMR (Bruker) apparatus (300 MHz ^{1}H-NMR, 75 MHz ^{13}C-NMR) or on a 500 MHz NMR (Bruker) apparatus (500 MHz ^{1}H-NMR, 125 MHz ^{13}C-NMR). Chemical shifts are reported in parts per million (ppm) downfield of TMS (tetramethylsilane) using the resonance of the deuterated solvent as internal standard. Proton couplings are described as s (singlet), d (doublet), t (triplet), q (quartet), br (broad) and m (multiplet), coupling constants are reported in Hertz. NMR data was analysed using Mnova 10.0 software (Mestrelab).

A Jasco V-650 spectrophotometer (Jasco, Japan) and a Jasco Model FP-6200 spectrofluorometer (Jasco, Japan) were used to collect absorption and emission spectra, respectively. Spectra were measured using 1 cm path quartz cuvettes and Milli-Q water as solvent. The final concentration of all PAMAM dendrimers was 0.4 mM.

A size-exclusion chromatography system (HPLC: Dionex Ultimate ® 3000, Column: TSKgel® GMPWXL HPLC Column) coupled with a multiangle light scattering (miniDAWN TREOS-AQUEOUS) and refractive index (RI Detector Refractomax 521) detectors were used to acquire the RI and LS signals. The number and weight average molecular mass values were obtained using ASTRA software. The SEC-MALS-RI system and ASTRA software were purchased from Wyatt technology Europe (Dernbach, Germany), and the column was from Sigma Aldrich (Denmark). Citrate buffer (pH 2.9) was used as solvent to dissolve amine-terminated dendrimers, while pyrrolidone- and carboxy-Tris-terminated

dendrimers were dissolved in phosphate buffer (pH 7.4). A 50 µL volume of sample solution prefiltered with 0.2 µm Acrodisc ® Syringe Filter (Supor ® Membrane) was injected into the system with a flow rate of 0.5 $mLmin^{-1}$.

MALDI was measured on a Bruker SolariX XR instrument in positive mode with a SA matrix. For HPLC-MS analysis a Dionex Ultimate 300 PLC connected to an ESI-MS (MSQ Plus Mass Spectrometer, Dionex) was used.

IR spectra were recorded on an FT-IR instrument using the attenuated total reflectance (ATR) sampling technique, and the measurements were carried out on a thin film of each sample obtained by evaporation from a solution of deuterated chloroform or methanol.

Core: DAB-PAMAM-(CO_2Me)$_4$ [1]. Under nitrogen atmosphere, methyl acrylate (166 g, 1.93 mol) dissolved in methanol (100 mL) was cooled to 0 °C, using an ice bath, and 1,4-diaminobutane (15.15 g, 0.172 mol) dissolved in methanol (75 mL) was added dropwise to the acrylate solution over 1 h. The reaction was stirred overnight at room temperature. TLC was used to monitor full conversion. The excess methyl acrylate and methanol were removed using a rotary evaporator. The final product was gained as colourless oil with a yield of 97% (71.55 g, 0.167 mol).

G0: DAB-PAMAM-(NH_2)$_4$ [2]. DAB-PAMAM-Core (71.55 g, 0.167 mol) was dissolved in methanol (850 mL). Ethylene diamine (EDA) (524 g, 8.7 mol, 13 eq. per surface group) was dissolved in methanol (110 mL) and cooled to 0 °C, using an ice bath. The dendrimer solution was added to this solution dropwise over 1 h under nitrogen atmosphere. The reaction was stirred for 4 days at room temperature and kept under vacuum. After that, azeotropic distillation with a mixture of methanol/toluene 1/9 was performed until all EDA was removed in vacuum. The excess toluene was removed by azeotropic distillation with methanol. The final compound was a colourless oil (91.5 g, 0.167 mol).

General Synthesis of DAB-PAMAM dendrimer of generation (G) 0.5 to G5. The PAMAM dendrimer synthesis consists of a repeating series of two reactions, first a branching of the amine unit to two branches using a Michael addition of two methyl acrylate molecules per amine, this leads to the half-generation dendrimers G0.5; G1.5; G2.5; G3.5 and G4.5. This step is always followed by an "activation" step where EDA forms an amide, substituting the outer methyl esters, which yields to new primary amines as the outer dendrimer layer (full generation dendrimers G1; G2; G3; G4 and G5).

General synthesis of half generation dendrimers. Methyl acrylate (3 eq. per dendrimer amine surface group) was dissolved in methanol (typical same volume as the methyl acrylate) and cooled with an ice bath to 0 °C. Under nitrogen atmosphere, DAB-PAMAM-Gn (1 eq.) dissolved in methanol (10 w/w %) was added dropwise over 1 h. The reaction was stirred for two days at room temperature. Full conversion was checked by KAISER test (1 % ninhydrin in ethanol) for remaining amines. The solvent and excess methyl acrylate was removed on a rotary evaporator, followed by high vacuum. This gave the half-generation dendrimer in form of a slight yellowish oil.

General synthesis of full generation dendrimers. DAB-PAMAM-half-generation (1 eq.) was dissolved in methanol (10 w/w %). EDA (25 eq. per ester surface group) was dissolved in methanol (typical 25% of the EDA volume) and cooled to 0 °C, using an ice bath. The dendrimer solution was added to this solution dropwise over 1 h under nitrogen atmosphere. The reaction was stirred for 4 days at room temperature. Methanol and EDA was removed under vacuum. After that, azeotropic distillation with a mixture of methanol/toluene 1/9 was performed until all EDA was removed under vacuum. The excess toluene was removed by azeotropic distillation with methanol. Dendrimers of generations G2-5 were further purified by dialysis against first water and then methanol. The full generation dendrimer was typically gained in form of white foam.

Surface functionalization of PAMAM dendrimers

General procedure for 1-(4-carbomethoxypyrrolidone) terminated PAMAM dendrimers. A 10 w/w% solution of dendrimer was added slowly to a 70 w/w% solution of dimethyl itaconate in methanol (1.05 equivalents per amine surface group), while cooling to 0 °C. Upon completion of the addition, the reaction mixture was left in the cooling bath to slowly heat to ambient temperature. The reaction was stirred until the KAISER-test (1% ninhydrin in ethanol) was negative, usually 2-4 days depending on the generation. The functionalized dendrimer was isolated by removing methanol, dissolving the dendrimer in a minimum amount of water, and multiple extraction times with diethyl ether until any remaining dimethyl itaconate had been removed. Higher dendrimer generations (G2–G5) were dialysed against first methanol then water. The aqueous phase was lyophilized to give the pure and quantitative covered 1-(4-carbomethoxypyrrolidone) terminated PAMAM dendrimers as white solids.

Dimethyl itaconate [13]. Itaconic acid (30 g, 0.23 mol) was dissolved in methanol (400 mL) and acetyl chloride (89 mL, 1.2 mol, 5 eq.) was added carefully, while cooling the reaction mixture with a water/ice bath. After complete addition, the reaction was heated to reflux for 1 h and then stirred for 72 h at room temperature. Volatiles were removed in vacuum and the solid material was dissolved in diethyl ether (300 mL) and extracted three times with slightly basic water (300 mL, pH approximately 10). The ether phase was dried over $MgSO_4$ and evaporated. After 24 h vacuum the compound was gained in form of colorless crystals (18.1 g, 11.4 mmol, 50 %).

General procedure for carboxy-Tris terminated PAMAM dendrimers. The PAMAM dendrimer was dissolved in methanol (10 wt%) and reacted with 2,5-dioxopyrrolidin-1-yl methyl succinate ("NHS activated methylsuccinic acid", 1.05 eq. per dendrimer-NH_2 surface group) which was directly added to the solution. The reaction mixture was stirred

for two days. To this solution (10 wt%) of the methyl succinate functionalized dendrimer in dry methanol was added 2-amino-2-hydroxymethyl-propane-1,3-diol (1.20 eq. per dendrimer methyl ester surface group). Anhydrous potassium carbonate was added in catalytic amounts (10 wt%) to promote the reaction. After two days water was added to the reaction mixture to hydrolyze the remaining succinic methyl esters. The potassium carbonate, NHS and the excess 2-amino-2-hydroxymethyl-propane-1,3-diol were removed by dialysis. After removal of the solvent the dendrimer was gained as a white solid. It needs to be noted that the introduction of TRIS groups is purely statistical. Distribution of Tris groups on the dendrimer surface is statistical. The number of attached Tris groups was calculated by comparing the integral of the Tris signals with the integral of the PAMAM dendrimers. It was calculated to be 6 Tris groups for the G4-Dendrimer and 12 Tris groups for the G5-Dendrimer.

2,5-Dioxopyrrolidin-1-yl methyl succinate [20]. Freshly distilled methyl 4-chloro-4-oxobutanoate (5g, 33.2 mmol) was dissolved in THF (50 mL) and cooled to 0 °C with a water/ice bath. Triethyl amine (4.85 mL, 1.08 eq) was slowly added, followed by the addition of N-hydroxysuccinimide (3.82 g, 33.5 mmol, 1.01 eq.). The reaction mixture was slowly allowed to warm to ambient temperature and stirred overnight. The triethyl ammonia salt was removed by filtration followed by removal of volatiles in vacuum. The final product was gained after recrystallization from 2-propanol in form of a white crystalline solid (4.8 g, 21 mmol, 63 %).

Covalent attachment of phthalocyanine (Pc) to G4 pyrrolidonated dendrimers. Pc (173 mg, 0.225 mmol) was dissolved in dichloromethane (15 mL). A solution of N-hydroxysuccinimide (52 mg, 0.45 mmol, 2 eq.) in anhydrous DMSO (30 mL) was added to the Pc solution followed by the addition of N,N'-dicyclohexylcarbodiimide (65.8 mg, 0.315 mmol) to the reaction mixture. The reaction was stirred overnight at room

temperature and under nitrogen and the formed 1,3-dicyclohexylurea side product was filtered of the reaction mixture followed by a removal of the solvent under reduced pressure. The product was then dissolved in DMSO. This solution was added slowly to an ice bath cooled solution of G4-PAMAM dendrimer (1.60 g, 0.1125 µmol → 2Pc per dendrimer) in dry methanol (20 mL). The reaction was stirred 2 days, followed by a removal of insoluble side products by filtration . The dendrimer-Pc solution was then directly used for dendrimer surface functionalization without further purification.

The PAMAM dendrimer solution from the previous reaction (Pc coupling) was directly taken and added drop wise to an ice bath cooled solution of dimethyl itaconate (1.68 g, 10.6 mmol, 1.47 eq. per dendrimer surface group) dissolved in methanol (4 mL). The solution was cooled with an ice bath during the addition. The reaction was stirred for four days. The excess of dimethyl itaconate was removed by dialysis:

1. DMSO overnight (solution gets very blue → removed Pc),
2. Methanol for 2 days changing to fresh methanol every 24 hrs,
3. Water for 2 days changing to fresh water every 24 hrs,
4. The content of the dialysis bag was filtered through a 200 nm Millipore filter to remove any remaining particles and freeze-dried.

Covalent attachment of phthalocyanine (Pc) to G4 Carboxy$_{58}$-Tris$_{6}$ dendrimers. The activated Pc-NHS ester was prepared by dissolving Pc (222 mg, 0.289 mmol) Pc in acetonitrile (10 mL). N-hydroxysuccinimide (33 mg, 0.0291 mmol) dissolved in acetonitrile (10 mL) was added to the Pc solution followed by 58 mg (0.290 mmol) of N,N'-dicyclohexylcarbodiimide. The reaction was stirred overnight at room temperature and under nitrogen and the formed 1,3-dicyclohexylurea side-product was filtered of the reaction mixture followed by a removal of the acetonitrile in vacuo. The activated Pc-NHS ester (0.0375 mmol) was dissolved in dry DMSO (12 mL) and slowly added to a solution of G4-PAMAM dendrimer (123 mg, 18.5 µmol) in dry methanol (6 mL). The reaction was

stirred 4 days, followed by a removal of insoluble side products by filtration. 2,5-Dioxopyrrolidin-1-yl methyl succinate (525 mg, 2,34 mmol, 1.1 eq. per dendrimer surface group) (G. A. Digenis, B. J. Agha, K. Tsuji, M. Kato, M. Shinogi, J. Med. Chem. 1986, 29, 1468-1476) was added directly to the solution. The reaction mixture was stirred for two days and afterwards 2-amino-2-hydroxymethyl-propane-1,3-diol (327 mg, 2.41 mmol, 1.30 eq. per dendrimer surface group) was added. Anhydrous potassium carbonate was added in catalytic amounts (37.5 mg, 0.28 mmol) to promote the reaction. After four days stirring at ambient temperature the potassium carbonate and the excess 2-amino-2-hydroxymethyl-propane-1,3-diol were removed by dialysis against Milli-Q water (membrane: regenerated cellulose, cutoff 2000 Da).

G4 Carboxy$_{58}$-Tris$_{6}$ dendrimers with trapped phthalocyanine (Pc). The SuccTRIS G4-PAMAM dendrimer (259 mg, 9.5 µmol) was dissolved in methanol (25 mL) and the Pc (39 mg, 47 µmol, 5 eq. per dendrimer) dissolved in dichloromethane (7 mL) was added. After 20 min incubation time, a small amount of water (5 mL) was added and the mixture was stirred for another 15 minutes. The solvent was removed using a rotary evaporator. The dark blue compound was then taken up in water, filtered and freeze-dried.

Supplementary Table 1. Characteristics of intermediates and full generation dendrimers and phthalocyanine (Pc)-dendrimers

**Core: DAB-PAMAM-(CO_2Me)$_4$ [1].** MW: 432.5 - ^{1}H-NMR: (500 MHz, $CDCl_3$) [ppm]: δ = 1.33-1.45 (m, 4 H); 2.35-2.41 (m, 4 H); 2.43 (t, 8 H,^3J=7.2 Hz); 2.75 (t, 8 H,^3J=7.2 Hz); 3.66 (s, 12 H) - ^{13}C-NMR: (125 MHz, $CDCl_3$) [ppm]: δ = 24.83; 32.51; 49.20; 51.49; 53.61; 173.05 - MS: MALDI-TOF: m/ z calc. = 433.247 $[M+H]^+$; m/ z found = 433.254 $[M+H]^+$ - Yield: 71.55 g, 97%.
**G0: DAB-PAMAM-(NH_2)$_4$ [2].** MW: 544.7 - ^{1}H-NMR: (500 MHz, MeOD-d_4) [ppm]: δ = 1.30-1.36 (m, 4 H); 2.26 (t, 8 H,^3J=6.8 Hz); 2.34-2.43 (m, 4 H); 2.62 (t, 8 H,^3J=6.4 Hz); 2.65 (t, 8 H,^3J=6.8 Hz); 3.14 (t, 8 H,^3J=6.4 Hz) - ^{13}C-NMR: (125 MHz, MeOD-d_4) [ppm]: δ = 25.84; 34.59; 42.07; 43.05; 50.86; 54.45; 175.32 - MS: MALDI-TOF: m/ z calc. = 567.425 $[M+Na]^+$; m/ z found = 567.425 $[M+Na]^+$; SEC-MALS: Elution time = 19.55 min - UV/Vis: ε_{215nm} = 3560 $M^{-1}cm^{-1}$, ε_{280nm} = 30 $M^{-1}cm^{-1}$- Fluorescence: λ_{exc} = 360 nm, $\lambda_{em,}$max = 470 nm - FT-IR: $\tilde{\nu}$[cm^{-1}]= 3263 (m, ν_{strech}(N-H Amide); 3076 (w, ν_{strech}(C-H); 2934 (m, ν_{strech}(C-H); 2862 (m, ν_{strech}(C-H); 1629 (s, ν_{strech}(C=O Amide); 1545 (s, $\nu_{bending}$(N-H Amide); 1463 (s); 1432 (s); 1314 (s); 1232 (m); 1191 (s); 1116 (m); 1027 (w); 945 (w); 818 (m) - Yield: 91.5 g, 99%.
**G0.5: PAMAM(CO_2Me)$_8$ [3].** MW: 1233.5 - ^{1}H-NMR: (500 MHz, MeOD-d_4) [ppm]: δ = 1.46-1.54 (m, 4 H); 2.40 (t, 8 H,^3J=6.7 Hz); 2.49 (t, 16 H,^3J=6.7 Hz); 2.50-2.55 (m, 4 H); 2.58 (t, 8 H,^3J=6.4 Hz); 2.79 (t, 24 H,^3J=6.7 Hz); 3.28 (t, 8 H,^3J=6.4 Hz); 3.69 (s, 24 H) - ^{13}C-NMR: (125 MHz, MeOD-d_4) [ppm]: δ = 25.82; 33.61; 34.45; 38.46; 50.52; 50.81; 52.21; 53.80; 54.39; 174.75; 174.77 - MS: MALDI-TOF: m/ z calc. = 1233.719 $[M+H]^+$; m/ z found = 1233.719 $[M+H]^+$- Yield: 25.1g, 99%.
**G1: PAMAM(NH_2)$_8$ [4].** MW: 1457.9 - ^{1}H-NMR: (500 MHz, MeOD-d_4) [ppm]: δ = 1.46-1.53 (m, 4 H); 2.39 (t, 24 H,^3J=6.7 Hz); 2.48-2.53 (m, 4 H); 2.60 (t, 8 H,^3J=6.7 Hz); 2.75 (t, 16 H,^3J=6.3 Hz); 2.82 (t, 24 H,^3J=6.8 Hz); 3.27 (t, 24 H,^3J=6.3 Hz) - ^{13}C-NMR: (125 MHz, MeOD-d_4) [ppm]: δ = 25.81; 34.37; 34.83; 38.60; 42.07; 43.07; 50.83; 51.19; 53.55; 54.39; 174.85, 175.20 - MS: ESI-MS: m/ z calc. = 1458.059 $[M+H]^+$; m/ z found = 1458.071 $[M+H]^+$; SEC-MALS: Elution time = 19.05 min - UV/Vis: ε_{215nm} = 7930 $M^{-1}cm^{-1}$, ε_{280nm}= 90 $M^{-1}cm^{-1}$ - Fluorescence: λ_{exc} = 360 nm, $\lambda_{em,max}$ = 430 nm - FT-IR: $\tilde{\nu}$[cm^{-1}]= 3284 (m, ν_{strech}(N-H Amide); 3072 (w, ν_{strech}(C-H); 2936 (m, ν_{strech}(C-H); 2863 (m, ν_{strech}(C-H); 1639 (s, ν_{strech}(C=O Amide); 1552 (s, $\nu_{bending}$(N-H Amide); 1463 (s); 1360 (s); 1232 (m); 1198 (s); 1154 (m); 1127 (m); 1041 (w); 952 (w) - Yield: 16.7 g, 95%.
**G1.5: PAMAM(CO_2Me)$_{16}$ [5].** MW: 2835.3 - ^{1}H-NMR: (500 MHz, MeOD-d_4) [ppm]: δ = 1.48-1.54 (m, 4 H); 2.41 (t, 24 H,^3J=6.8 Hz); 2.49 (t, 32 H,^3J=6.7 Hz); 2.49-2.53 (m, 4 H); 2.58 (t, 16 H,^3J=6.5 Hz); 2.63 (t, 16 H,^3J=6.5 Hz); 2.79 (t, 32 H,^3J=6.7 Hz); 2.85 (t, 24 H,^3J=6.8 Hz); 3.28 (t, 24 H,^3J=6.5 Hz); 3.69 (s, 48 H) - ^{13}C-NMR: (125 MHz, MeOD-d_4) [ppm]: δ = 25.84; 33.62; 34.44; 34.78; 38.53; 38.65; 50.53; 50.85; 51.10; 52.24; 53.53; 53.83; 54.34; 174.68, 174.71; 174.77 - MS: MALDI-TOF: m/ z calc. = 1418.329 $[M+2H]^{2+}$; m/ z found = 1418.331 $[M+2H]^{2+}$- Yield: 32.5 g, 99%.

G2: PAMAM$(NH_2)_{16}$ [6]. MW: 3284.2 - ^{1}H-NMR: (500 MHz, MeOD-d_4) [ppm]: δ = 1.34-1.43 (m, 4 H); 2.27 (t, 56 H,^3J=6.9 Hz); 2.37-2.43 (m, 4 H); 2.48 (t, 24 H,^3J=6.6 Hz); 2.63 (t, 32 H,^3J=6.3 Hz); 2.70 (t, 56 H,^3J=6.9 Hz); 3.15 (t, 56 H,^3J=6.3 Hz) - ^{13}C-NMR: (125 MHz, MeOD-d_4) [ppm]: δ = 25.86; 34.47; 34.84; 36.63; 38.67; 39.98; 42.10; 43.09; 50.86; 51.18; 51.32; 53.56; 54.43; 174.75; 175.17 - MS: ESI-MS: m/ z calc. = 1642.669 $[M+2H]^{2+}$; m/ z found = 1642.713 $[M+2H]^{2+}$; SEC-MALS: Elution time= 18.45 min, Mw= 2,6 ± 0,3 kDa - UV/Vis: ε_{215nm} = 8490 $M^{-1}cm^{-1}$, ε_{280nm}= 190 $M^{-1}cm^{-1}$ - Fluorescence: λ_{exc} = 360 nm, $\lambda_{em,max}$ = 410 nm - FT-IR: $\tilde{\nu}[cm^{-1}]$= 3280 (m, ν_{strech}(N-H Amide); 3076 (w, ν_{strech}(C-H); 2935 (m, ν_{strech}(C-H); 2822 (m, ν_{strech}(C-H); 1633 (s, ν_{strech}(C=O Amide); 1543 (s, $\nu_{bending}$(N-H Amide); 1461 (s); 1434 (s); 1357 (s); 1248 (m); 1198 (s); 1151 (m); 1030 (w); 949 (w) - Yield: 8.1 g, 84%.

G2.5: PAMAM$(CO_2Me)_{32}$ [7]. MW: 6039.1 - ^{1}H-NMR: (500 MHz, MeOD-d_4) [ppm]: δ = 1.43-1.56 (m, 4 H); 2.41 (t, 56 H,^3J=6.7 Hz); 2.49 (t, 64 H,^3J=6.7 Hz); 2.50-2.54 (m, 4 H); 2.58 (t, 32 H,^3J=6.4 Hz); 2.61-2.67 (m, 24 H); 2.79 (t, 64 H, ^3J=6.7 Hz); 2.85 (t, 56 H, ^3J=6.8 Hz); 3.28 (t, 56 H,^3J=6.4 Hz); 3.69 (s, 96 H) - ^{13}C-NMR: (125 MHz, MeOD-d_4) [ppm]: δ = 25.87; 33.63; 34.47; 34.09; 34.89; 38.54; 38.68; 39.88; 50.55; 51.09; 51.17; 51.24; 52.26; 53.55; 53.84; 54.51; 174.6; 174.75 - Yield: 14.1 g, 95%.

G3: PAMAM$(NH_2)_{32}$ [8]. MW: 6936.9 - ^{1}H-NMR:(500 MHz, MeOD-d_4) [ppm]: δ = 1.32-1.43 (m, 4 H); 2.27 (t, 120 H,^3J=6.4 Hz); 2.37-2.43 (m, 4 H); 2.48-2.55(m, 60 H); 2.62-2.65 (m, 64 H); 2.65-2.73 (m, 120 H); 3.14-3.19 (m, 120 H) - ^{13}C-NMR: (125 MHz, MeOD-d_4) [ppm]: δ = 25.94; 34.84; 36.62; 38.67; 40.00; 42.07; 42.99; 51.18, 53.56; 54.71; 174.74; 175.18 - MS: ESI: m/ z calc. = 1388.2 $[M+5H]^{5+}$; m/ z found = 1388.3 $[M+5H]^{5+}$ SEC-MALS: Elution time = 18.10 min, Mw = 6,7 ± 2,1 kDa - UV/Vis: ε_{215nm} = 8820 $M^{-1}cm^{-1}$, ε_{280nm}= 320 $M^{-1}cm^{-1}$ - Fluorescence: λ_{exc} = 360 nm, $\lambda_{em,max}$ = - 410 nm - FT-IR: $\tilde{\nu}[cm^{-1}]$= 3273 (m, ν_{strech}(N-H Amide); 3081 (w, ν_{strech}(C-H); 2938 (m, ν_{strech}(C-H); 2828 (m, ν_{strech}(C-H); 1630 (s, ν_{strech}(C=O Amide); 1545 (s, $\nu_{bending}$(N-H Amide); 1462 (s); 1435 (s); 1356 (s); 1248 (m); 1199 (s); 1152 (m); 1027 (w); 950 (w) - Yield: 8.8 g, 55%.

G3.5: PAMAM$(CO_2Me)_{64}$ [9]. MW: 11698.0 - ^{1}H-NMR: (500 MHz, MeOD-d_4) [ppm]: δ = 1.45-1.53 (m, 4 H); 2.38 (t, 120 H,^3J=6.8 Hz); 2.47 (t, 128 H,^3J=6.8 Hz); 2.53-2.65 (m, 120 H,); 2.74-2.88 (m, 248 H); 3.26 (t, 120 H,^3J=6.8 Hz); 3.67 (s, 192 H) - ^{13}C-NMR: (125 MHz, MeOD-d_4) [ppm]: δ = 33.61; 34.77; 38.31; 38.64; 51.07; 52.24; 53.54; 53.82; 174.57; 174.67- Yield: 5.1 g, 94%.

G4: PAMAM$(NH_2)_{64}$ [10]. MW: 14242.5 - ^{1}H-NMR:(500 MHz, MeOD-d_4) [ppm]: δ = 1.40-1.51 (m, 4 H); 2.37 (t, 240 H,^3J=6.4 Hz); 2.53-2.65(m, 128 H); 2.69-2.78 (m, 120 H); 2.79-2.91 (m, 248 H); 3.22-3.31 (m, 248 H) - ^{13}C-NMR: (125 MHz, MeOD-d_4) [ppm]: δ = 34.85; 38.66; 42.10; 43.04; 51.19, 53.57; 174.69; 175.12 - MS: SEC-MALS: Elution time = 17.85 min, Mw = 15,4 ± 1,3 kDa - UV/Vis: ε_{215nm} = 8790 $M^{-1}cm^{-1}$, ε_{280nm}= 330 $M^{-1}cm^{-1}$ - Fluorescence: λ_{exc} = 360 nm, $\lambda_{em,max}$ = 420 nm - FT-IR: $\tilde{\nu}[cm^{-1}]$= 3276 (m, ν_{strech}(N-H Amide); 3068 (w, ν_{strech}(C-H); 2938 (m, ν_{strech}(C-H); 2830 (m, ν_{strech}(C-H); 1633 (s, ν_{strech}(C=O Amide); 1544 (s, $\nu_{bending}$(N-H Amide); 1462 (s); 1433 (s); 1319 (s); 1198 (s); 1153 (m); 1036 (w) - Yield: 4.7 g, 82%.

**G4.5: PAMAM(CO_2Me)$_{128}$ [11].** MW: 25142.1 - ^{1}H-NMR: (500 MHz, MeOD-d_4) [ppm]: δ = 1.46-1.57 (m, 4 H); 2.39 (t, 248 H,^3J=6.8 Hz); 2.47 (t, 256 H,^3J=6.8 Hz); 2.54-2.69 (m, 248 H,); 2.74-2.92 (m, 496 H); 3.22-3.331 (m, 248 H); 3.67 (s, 384 H) - ^{13}C-NMR: (125 MHz, MeOD-d_4) [ppm]: δ = 33.64; 34.78; 38.54; 38.67; 50.55; 51.09; 52.29; 53.57; 53.85; 174.59; 174.70 - Yield: 11.9, 99%.
**G5: PAMAM(NH_2)$_{128}$ [12].** MW: 28853.1 - ^{1}H-NMR:(500 MHz, MeOD-d_4) [ppm]: δ = 1.42-1.52 (m, 4 H); 2.37 (t, 504 H,^3J=6.4 Hz); 2.54-2.65(m, 248 H); 2.71-2.75 (m, 256 H); 2.76-2.94 (m, 504 H); 3.24-3.33 (m, 504 H) - ^{13}C-NMR: (125 MHz, MeOD-d_4) [ppm]: δ = 34.85; 38.66; 42.10; 43.04; 51.19, 53.57; 174.69; 175.12 - MS: SEC-MALS: Elution time = 17.45 min, Mw = 29,0 ± 5,2 - UV/Vis: ε_{215nm} = 10330 $M^{-1}cm^{-1}$, ε_{280nm}= 4680 $M^{-1}cm^{-1}$ - Fluorescence: λ_{exc} = 360 nm, $\lambda_{em,max}$ = 420nm - FT-IR: $\tilde{\nu}$[cm^{-1}]= 3275 (m, ν_{strech}(N-H Amide); 3076 (w, ν_{strech}(C-H); 2935 (m, ν_{strech}(C-H); 2830 (m, ν_{strech}(C-H); 1631 (s, ν_{strech}(C=O Amide); 1545 (s, $\nu_{bending}$(N-H Amide); 1463 (s); 1321 (s); 1200 (s); 1153 (m); 1036 (w) - Yield: 8.3 g, 61%.
**Dimethyl itaconate [13].** MW: 158.2 - ^{1}H-NMR:(500 MHz, MeOD-d_4) [ppm]: δ = 3.37 (s, 2H); 3.69 (s, 3H); 3.76 (s, 3H); 5.77 (s, 1H); 6.28 (s, 1H) - ^{13}C-NMR: (125 MHz, MeOD-d_4) [ppm]: δ = 38.18; 52.44; 52.55; 129.34; 135.46; 168.16; 173.00 - MS: (MALDI) m/ z calc. = 181.0471 $[M+H]^+$; m/ z found = 181.0651 $[M]^+$ - Yield: 18.1 g, 50%.
**G0: PAMAM(Pyr)$_4$ [14].** MW: 1049.2 - ^{1}H-NMR:(500 MHz, MeOD-d_4) [ppm]: δ = 1.42-1.56 (m, 4 H); 2.36 (t, 8 H,^3J=6.8 Hz); 2.49-2.53 (m, 4 H); 2.63-2.69 (m, 8 H); 2.78 (t, 8 H,^3J=6.8 Hz); 3.35-3.48 (m, 20 H); 3.65-3.81 (m, 20 H) - ^{13}C-NMR: (125 MHz, MeOD-d_4) [ppm]: δ = 25.38; 34.09; 34.99; 37.13; 37.49; 43.33; 50.60; 52.92; 54.09; 174.93; 175.07; 175.67 - MS: MALDI: m/ z calc. = 1049.551 $[M+H]^+$; m/ z found = 1049.552 $[M+H]^+$ - UV/Vis: ε_{280nm}= 30 $M^{-1}cm^{-1}$ - Fluorescence: λ_{exc} = 360 nm, $\lambda_{em,max}$ =440 nm - FT-IR: $\tilde{\nu}$[cm^{-1}]= 3283(m, ν_{strech}(N-H Amide); 3076(w, ν_{strech}(C-H); 2949 (m, ν_{strech}(C-H); 1732 (s, ν_{strech}(C=O Ester); 1655 (s, ν_{strech}(C=O Amide); 1554 (s, $\nu_{bending}$(N-H Amide); 1493 (s); 1435 (s); 1363 (s); 1268 (m); 1123 (m); 1023 (w); 939 (w); 698 (w); 665 (w); 573 (m) - Yield: 0.35 g, 45%.
**G1: PAMAM(Pyr)$_8$ [15].** MW: 2466.8 - ^{1}H-NMR:(500 MHz, MeOD-d_4) [ppm]: δ = 1.44-1.53 (m, 4 H); 2.33 (t, 16 H,^3J=6.9 Hz); 2.42 (t, 8 H,^3J=6.9 Hz); 2.58 (t, 12 H,^3J=6.9 Hz); 2.62-2.69 (m, 16 H); 2.77 (t, 16 H,^3J=6.9 Hz); 2.84 (t, 8 H,^3J=6.9 Hz); 3.27 (t, 8 H,^3J=6.9 Hz); 3.32-3.45 (m, 40 H); 3.65-3.81 (m, 40 H) - ^{13}C-NMR: (125 MHz, MeOD-d_4) [ppm]: δ = 25.43; 33.98; 34.45; 35.01; 37.13; 37.53; 38.46; 43.35; 50.57; 50.83; 52.95; 53.32; 54.30; 174.50; 174.94; 175.07; 175.63 - MS: MALDI: m/ z calc. = 2467.316 $[M+H]^+$; m/ z found = 2467.349 $[M+H]^{2+}$, m/ z calc. = 1234.166 $[M+2H]^{2+}$; m/ z found = 1234.168 $[M+2H]^{++}$, m/ z calc. = 823.114 $[M+3H]^{3+}$; m/ z found = 823.114 $[M+3H]^{3+}$ - UV/Vis: ε_{215nm} = 8200 $M^{-1}cm^{-1}$, ε_{280nm}= 320 $M^{-1}cm^{-1}$ - Fluorescence: λ_{exc} = 360 nm, $\lambda_{em,max}$ =440 nm - FT-IR: $\tilde{\nu}$[cm^{-1}]= 3294(m, ν_{strech}(N-H Amide); 3078(w, ν_{strech}(C-H); 2950 (m, ν_{strech}(C-H); 1735 (s, ν_{strech}(C=O Ester); 1663 (s, ν_{strech}(C=O Amide); 1548 (s, $\nu_{bending}$(N-H Amide); 1493 (s); 1436 (s); 1363 (s); 1269 (m); 1204 (s); 1022 (w); 938 (w); 700 (w); 577 (m) - Yield: 1.7 g, 90%.
**G2: PAMAM(Pyr)$_{16}$ [16].** MW: 5302.0 - ^{1}H-NMR:(500 MHz, MeOD-d_4) [ppm]: δ = 1.45-

1.54 (m, 4 H); 2.35 (t, 32 H,^3J=6.7 Hz); 2.41 (t, 24 H,^3J=6.7 Hz); 2.54 (t, 24 H,^3J=6.7 Hz); 2.63-2.70 (m, 32 H); 2.79 (t, 32 H,^3J=6.7 Hz); 2.80-2.88 (m, 24 H); 3.30 (t, 24 H,^3J=6.7 Hz); 3.35-3.49 (m, 80 H); 3.67-3.86 (m, 80 H) - ^{13}C-NMR: (125 MHz, MeOD-d_4) [ppm]: δ = 25.73; 34.34; 34.51; 34.73; 35.03; 37.16; 37.56; 38.48; 38.65; 43.38; 50.59; 50.86; 51.13; 52.98; 53.38; 53.50; 54.43; 174.70; 174.97; 175.09; 175.65 - MS: MALDI: m/ z calc. = 5301.8 $[M+H]^+$; m/ z found = 5304.7 $[M+H]^+$ - ESI: m/ z calc. = 1326.2 $[M+4H]^{4+}$; m/ z found = 1326.3 $[M+4H]^{4+}$; SEC-MALS: Elution time = 17.90 min, Mw = 5,2 ± 0,1 kDa - UV/Vis: ε_{215nm} = 8430 $M^{-1}cm^{-1}$, ε_{280nm}= 440 $M^{-1}cm^{-1}$ Fluorescence: λ_{exc} = 360 nm, $\lambda_{em,max}$ = 450 nm - FT-IR: $\tilde{\nu}$[cm^{-1}]= 3284(m, ν_{strech}(N-H Amide); 3079(w, ν_{strech}(C-H); 2938 (m, ν_{strech}(C-H); 1735 (s, ν_{strech}(C=O Ester); 1649 (s, ν_{strech}(C=O Amide); 1552 (s, $\nu_{bending}$(N-H Amide); 1493 (s); 1436 (s); 1365 (s); 1267 (m); 1204 (s); 1024 (w); 699 (m); 670 (w); 572 (m) - Yield: 0.61 mg, 98%.
**G3: PAMAM(Pyr)$_{32}$ [17].** MW: 10972.4 - ^{1}H-NMR:(500 MHz, MeOD-d_4) [ppm]: δ = 1.51-1.58 (m, 4 H); 2.29-2.36 (m, 64 H); 2.37-2.44 (m, 56 H); 2.45-2.49 (m, 4 H); 2.56-2.64 (m, 56 H); 2.65-2.70 (m, 64 H); 2.76-2.82 (m, 64 H); 2.83-2.89 (m, 56 H); 3.26-3.34(m, 56 H); 3.35-3.56 (m, 160 H); 3.64-3.82 (m, 160 H) - ^{13}C-NMR: (125 MHz, MeOD-d_4) [ppm]: δ = 34.55; 34.76; 34.84; 35.06; 37.03; 37.59; 38.49; 38.64; 43.40; 50.08); 50.61; 50.89; 51.14; 53.01; 53.43; 174.64; 174.78; 174.96; 175.03; 175.61 - MS: MALDI m/ z calc. = 10971.9 $[M+H]^+$; m/ z found = 10973.6 $[M+H]^+$; SEC-MALS: Elution time = 17.30 min, Mw = 12,1 ± 0,1 kDa - UV/Vis: ε_{215nm} = 9490 $M^{-1}cm^{-1}$, ε_{280nm}= 2050 $M^{-1}cm^{-1}$ - Fluorescence: λ_{exc} = 360 nm, $\lambda_{em,max}$ = 440 nm - FT-IR: $\tilde{\nu}$[cm^{-1}]= 3287 (m, ν_{strech}(N-H Amide); 3081 (w, ν_{strech}(C-H); 2949 (m, ν_{strech}(C-H); 2830 (m, ν_{strech}(C-H); 1734 (s, ν_{strech}(C=O Ester); 1644 (s, ν_{strech}(C=O Amide); 1547 (s, $\nu_{bending}$(N-H Amide); 1494 (s); 1435 (s); 1362 (s); 1268 (m); 1203 (s); 1023 (w); 939 (w); 729 (w); 698 (m); 573 (m) - Yield: 6.4 g, 96%.
**G4: PAMAM(Pyr)$_{64}$ [18].** MW: 22313.6 - ^{1}H-NMR:(500 MHz, MeOD-d_4) [ppm]: δ = 1.45-1.52 (m, 4 H); 2.28-2.35 (m, 128 H); 2.36-2.44 (m, 120 H); 2.56-2.63 (m, 120 H); 2.64-2.69 (m, 128 H); 2.73-2.79 (m, 128 H); 2.80-2.89 (m, 120 H); 3.25-3.31 (m, 120 H); 3.35-3.47 (m, 320 H); 3.64-3.81 (m, 320 H) - ^{13}C-NMR: (125 MHz, MeOD-d_4) [ppm]: δ = 34.23; 34.52; 34.74; 35.05; 37.17; 37.58; 38.49; 38.65; 43.39; 50.02; 50.14; 50.60; 50.88; 51.12; 52.93; 53.04; 53.40; 54.70; 174.66; 174.97; 175.05; 175.60; 175.72 MS: SEC-MALS: Elution time = 16.95 min, Mw = 23,6 ± 0,1 kDa - UV/Vis: ε_{215nm} = 8800 $M^{-1}cm^{-1}$, ε_{280nm}= 320 $M^{-1}cm^{-1}$ - Fluorescence: λ_{exc} = 360 nm, $\lambda_{em,max}$ = 440 nm - FT-IR: $\tilde{\nu}$[cm^{-1}]= 3284 (m, ν_{strech}(N-H Amide); 3079 (w, ν_{strech}(C-H); 2947 (m, ν_{strech}(C-H); 2838 (m, ν_{strech}(C-H); 1734 (s, ν_{strech}(C=O Ester); 1643 (s, ν_{strech}(C=O Amide); 1546 (s, $\nu_{bending}$(N-H Amide); 1494 (s); 1435 (s); 1362 (s); 1268 (m); 1202 (s); 1022 (w); 939 (w); 729 (w); 698 (m); 572 (m) - Yield: 2.6 g, 91%.
**G5: PAMAM(Pyr)$_{128}$ [19].** MW: 44996.0 - ^{1}H-NMR:(500 MHz, MeOD-d_4) [ppm]: δ = 2.27-2.35 (m, 248 H); 2.36-2.45 (m, 256 H); 2.53-2.63 (m, 248 H); 2.64-2.70 (m, 256 H); 2.74-2.79 (m, 256 H); 2.80-2.91 (m, 248 H); 3.23-3.30 (m, 248 H); 3.35-3.45 (m, 640 H); 3.62-3.83 (m, 640 H) - ^{13}C-NMR: (125 MHz, MeOD-d_4) [ppm]: δ = 34.54; 34.73; 35.07; 37.19; 37.61; 38.50; 38.64; 43.40; 50.60; 50.90; 51.14; 53.06; 53.44; 53.57; 174.51; 174.59;

174.95; 175.54 - MS: SEC-MALS: Elution time = 16.75 min, Mw = 49,0 ± 0,1 kDa - UV/Vis: ε_{215nm} = 10140 $M^{-1}cm^{-1}$, ε_{280nm}= 2800 $M^{-1}cm^{-1}$ - Fluorescence: λ_{exc} = 360 nm, $\lambda_{em,max}$ = 430nm - FT-IR: $\tilde{\nu}$[cm^{-1}]= 3284 (m, ν_{strech}(N-H Amide); 3079 (w, ν_{strech}(C-H); 2947 (m, ν_{strech}(C-H); 2838 (m, ν_{strech}(C-H); 1734 (s, ν_{strech}(C=O Ester); 1643 (s, ν_{strech}(C=O Amide); 1546 (s, $\nu_{bending}$(N-H Amide); 1494 (s); 1435 (s); 1363 (s); 1268 (m); 1202 (s); 1022 (w); 939 (w); 729 (w); 698 (m); 572 (m) - Yield: 2.9 g, 92%.
**2,5-Dioxopyrrolidin-1-yl methyl succinate [20].** MW: 229.2 - ^{1}H-NMR:(500 MHz, MeOD-d_4) [ppm]: δ = 2.74 (t, J = 7.0 Hz, 2H); 2.83 (s, 4H); 2.95 (t, J = 7.0 Hz, 2H); 3.72 (s, 3H) - ^{13}C-NMR: (125 MHz, MeOD-d_4) [ppm]: δ = 25.70; 26.44; 28.68; 52.29; 167.84; 169.02; 171.52 - MS: (MALDI) m/ z calc. = 252.0479 $[M+H]^+$; m/ z found = 252.0484 $[M+H]^+$ - Yield: 4.8 g, 63%.
**G4: PAMAM-$(COOH)_{58}(Tris)_6$ [21].** MW: 21233.5 - ^{1}H-NMR:(500 MHz, MeOD-d_4) [ppm]: δ = 1.42-1.49 (m, 4 H); 2.24-2.32 (m, 120 H); 2.33-2.43 (m, 180 H); 2.55-2.66 (m, 180 H); 2.66-2.71 (m, 130 H); 2.72-2.77 (m, 130 H); 2.78-2.86 (m, 130 H); 3.24-3.31 (m, 160 H); 3.33-3.39 (m, 120 H); 3.57-3.64 (m, 120 H); 3.66 (s, 36) - ^{13}C-NMR: (125 MHz, MeOD-d_4) [ppm]: δ = 29.33; 30.20; 31.53; 34.46; 34.78; 38.16; 38.45; 39.62; 40.05; 50.87; 51.16; 52.41; 53.37; 174.70; 175.13; 180.07 - MS: SEC-MALS: Elution time = 17.45 min, Mw = 18,6 ± 0,1kDa - UV/Vis: ε_{215nm} = 167140 $M^{-1}cm^{-1}$, ε_{280nm}= 2830 $M^{-1}cm^{-1}$ -Fluorescence: λ_{exc} = 360 nm, $\lambda_{em,max}$ =440 nm - FT-IR: $\tilde{\nu}$[cm^{-1}]= 3274 (m, ν_{strech}(N-H Amide); 3079 (w, ν_{strech}(C-H); 2941 (m, ν_{strech}(C-H); 1697 (s, ν_{strech}(C=O Carboxy); 1633 (s, ν_{strech}(C=O Amide); 1543 (s, $\nu_{bending}$(N-H Amide); 1432 (s); 1401 (s); 1359 (s); 1253 (s); 1110 (m); 1040 (w); 818 (m) - Yield: 2.8 g, 82%.
**G5: PAMAM-$(COOH)_{118}(Tris)_{12}$ [22].** MW: 43620.1 - ^{1}H-NMR:(500 MHz, MeOD-d_4) [ppm]: δ = 2.25-2.33 (m, 250 H); 2.34-2.43 (m, 380 H); 2.49-2.64 (m, 380 H); 2.65-2.71 (m, 510 H); 2.66-2.72 (m, 250 H); 2.73-2.77 (m, 340 H); 2.78-2.86 (m, 130 H); 3.23-3.29 (m, 240 H); 3.35-3.41 (m, 250 H); 3.56-3.64 (m, 250 H); 3.66 (s, 60) ^{13}C-NMR: (125 MHz, MeOD-d_4) [ppm]: δ = 29.31; 30.19; 31.51; 34.46; 34.79; 38.16; 39.61; 40.18; 50.87; 51.16; 53.28; 53.52; 63.65; 174.68; 175.24; 180.08 - MS: SEC-MALS: Elution time = 16.65 min, Mw = 47,6 ± 0,2 kDa - UV/Vis: ε_{215nm} = 218830 $M^{-1}cm^{-1}$, ε_{280nm}= 2940 $M^{-1}cm^{-1}$ -Fluorescence: λ_{exc} = 360 nm, $\lambda_{em,max}$ = 450 nm - FT-IR: $\tilde{\nu}$[cm^{-1}]= 3269 (m, ν_{strech}(N-H Amide); 3078 (w, ν_{strech}(C-H); 2943 (m, ν_{strech}(C-H); 1635 (s, ν_{strech}(C=O Amide); 1544 (s, $\nu_{bending}$(N-H Amide); 1397 (s); 1251 (s); 1156 (m); 1037 (w) - Yield: 0.58 g, 86%.
**Pc-G4 Pyr (Pc covalently attached).** ^{1}H-NMR (500 MHz, MeOD-d_4) [ppm]: δ = 1.83-1.99 (m, 54 H); 2.28-2.46 (m, 320 H); 2.55-2.72 (m, 300 H); 2.74-2.90 (m, 290 H); 3.22-3.30 (m, 120 H); 3.34-3.48 (m, 300 H); 3.63-3.82 (m, 310 H) - ^{13}C-NMR (125 MHZ, MeOD-d_4) [ppm]: δ = 34.37; 35.08; 37.20; 37.61; 38.42; 43.39; 49.56; 50.63; 50.92; 51.18; 52.30; 53.05; 53.57; 174.47; 174.97; 175.59. UV-VIS in DMSO (ε_{680nm}: 23500 $M^{-1}cm^{-1}$). MW: MALDI-TOF MS: 23609.5 $(M+H)^+$. Loading of Pc estimated by UV-VIS: 1.95 Pc per dendrimer. Yield: 0.82 g (32%). Final appearance: blue powder.
**Pc-G4 $Carboxy_{58}$-$Tris_6$ (Pc covalently attached).** ^{1}H-NMR (500 MHz, MeOD-d_4) [ppm]: δ =

1.42–1.49 (m, 4 H); 2.24–2.32 (m, 120 H); 2.33–2.43 (m, 180 H); 2.55–2.66 (m, 180 H); 2.66–2.71 (m, 130 H); 2.72–2.77 (m, 130 H); 2.78–2.86 (m, 130 H); 3.24–3.31 (m, 160 H); 3.33-3.39 (m, 120); 3.57–3.64 (m, 120 H); 3.66 (s, 36 H) - ^{13}C NMR (125 MHZ, MeOD-d_4) [ppm]: δ = 28.04, 31.04, 31.44, 32.46, 33.13, 38.30, 38.49, 38.63, 38.69, 38.77, 47.86, 49.07, 51.55, 59.54, 60.41, 62.07, 174.15, 175.13, 176.01, 180.79, 181.34. UV-VIS in DMSO (ε_{680nm}: 477000 $M^{-1}cm^{-1}$). Loading of Pc estimated by UV-VIS: 3.0 Pc per dendrimer. Yield: 125 mg, 8.0 μmol (43%). Final appearance: blue gel.
**Pc-G4 Carboxy$_{58}$-Tris$_6$ (Pc non-covalently associated).** ^{1}H-NMR (500 MHz, MeOD-d_4) [ppm]: δ = 1.42–1.49 (m, 4 H); 2.24–2.32 (m, 120 H); 2.33–2.43 (m, 180 H); 2.55–2.66 (m, 180 H); 2.66–2.71 (m, 130 H); 2.72–2.77 (m, 130 H); 2.78–2.86 (m, 130 H); 3.24–3.31 (m, 160 H); 3.33-3.39 (m, 120); 3.57–3.64 (m, 120 H); 3.66 (s, 36 H) - ^{13}C-NMR (125 MHZ, MeOD-d_4) [ppm]: δ = 29.33; 30.20; 31.53; 34.46; 34.78; 38.16; 38.45; 39.62; 40.05; 50.87; 51.16; 52.41; 53.37; 174.70; 175.13; 180.07. UV-VIS in DMSO (ε_{680nm}: 640000 $M^{-1}cm^{-1}$). Loading of Pc estimated by UV-VIS: 3.0 Pc per dendrimer. Yield: 252 mg, 8.3 μmol (87%). Final appearance: dark blue solid.

Supplementary Table 2. Mean MBL levels in plasma samples

Plasma code	M26	M29	F33	M48	F36	F50	M61	M63	F55
MBL ($ngmL^{-1}$)	2100 ± 107	78 ± 11	1888 ± 96	111 ± 21	86 ± 23	988 ± 132	875 ± 81	1381 ± 162	1403 ± 75

Mean values and s.d. are from 3 determinations and each in triplicate.

Supplementary Table 3. Characteristics and complement activation properties of phthalocyanine (Pc)-loaded $Carboxy_{58}$-$Tris_6$ dendrimers

Formulation	Estimated Pc content (molecules/dendrimer)	Mean size ± s.d.[3]	sC5b-9 (fold increase)[4]	C5a (fold increase)[4]
Pc-G4 Carboxy-Tris[1]	3 (covalently attached)	58 ± 4 nm	0.98 ± 0.05	0.91 ± 0.05
Pc-G4 Carboxy Tris[1]	3 (non-covalently associated)	62 ± 7 nm	1.34 ± 0.07	1.05 ± 0.05
Zymosan (control)[2]	-	-	21.26 ± 1.21	13.5 ± 0.91

[1]The final concentration of Pc-dendrimers in human plasma for complement activation studies was 3.5 $mgmL^{-1}$.
[2]The final concentration of zymosan in human plasma for complement activation studies was 0.2 $mgmL^{-1}$.
[3]Nanoparticle Tracking Analysis was used to estimate mean hydrodynamic particle sizes after dilution in Milli-Q water at 21 °C. Each experiment was repeated three times and the results are presented as hydrodynamic mean sizes ± s.d.
[4]Complement activation in typical human plasma. Complement activation was followed through measurements of fluid-phase levels of sC5b9 and C5a by ELISA. Complement activation is reported as fold increase over the respective background (plasma without Pc-dendrimesr or on zymosan addition). The results are means of three technical replicates ± s.d. and each experiment was done in triplicate samples. The results were not statistically significant compared with their respective background ($p>0.05$; unpaired, two-sided).

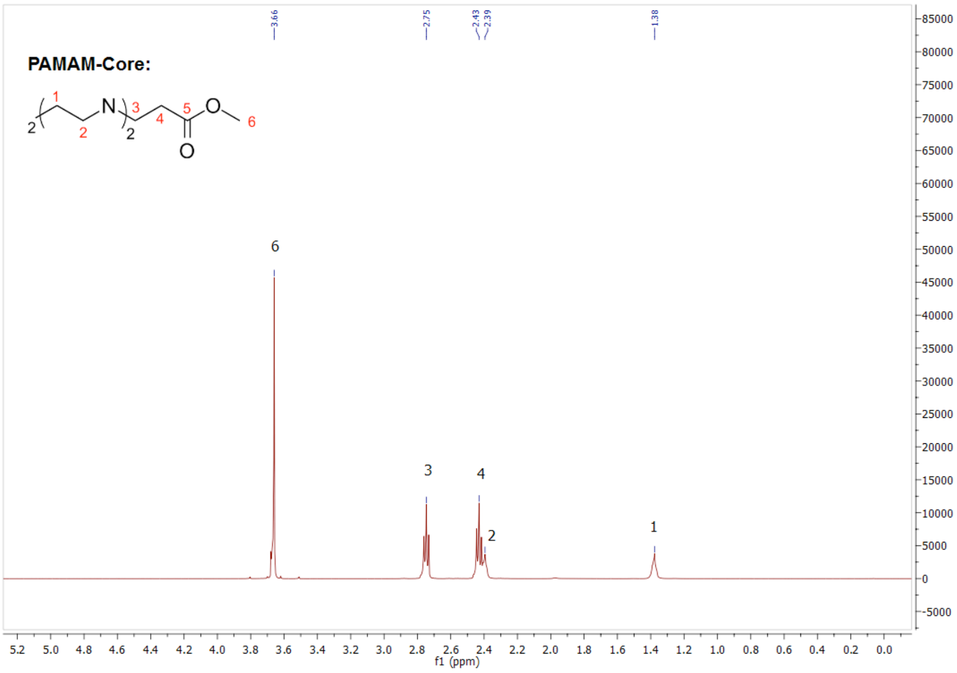

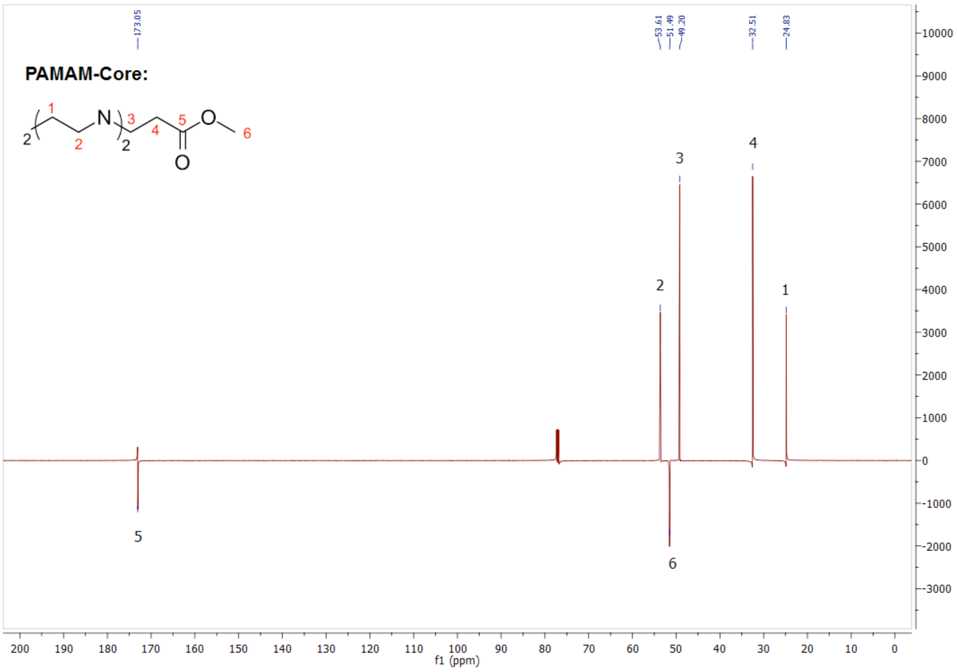

Supplementary Fig. 1. NMR Spectra of DAB-PAMAM-$(CO_2Me)_4$ [1]. Top: ^{1}H-NMR (500 MHz, MeOD-d_4). Bottom: ^{13}C-NMR (125 MHz, MeOD-d_4).

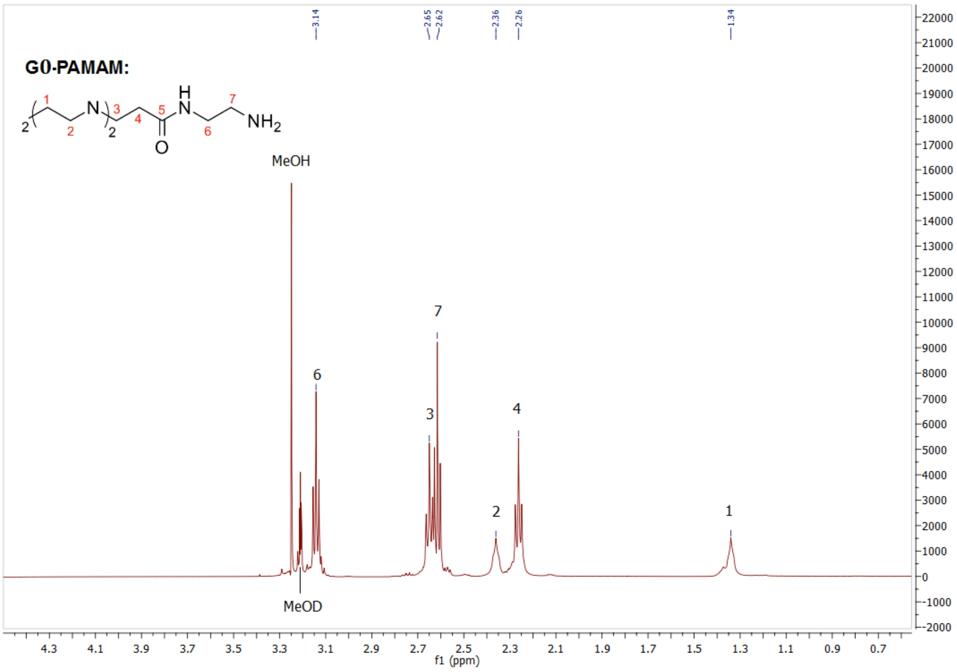

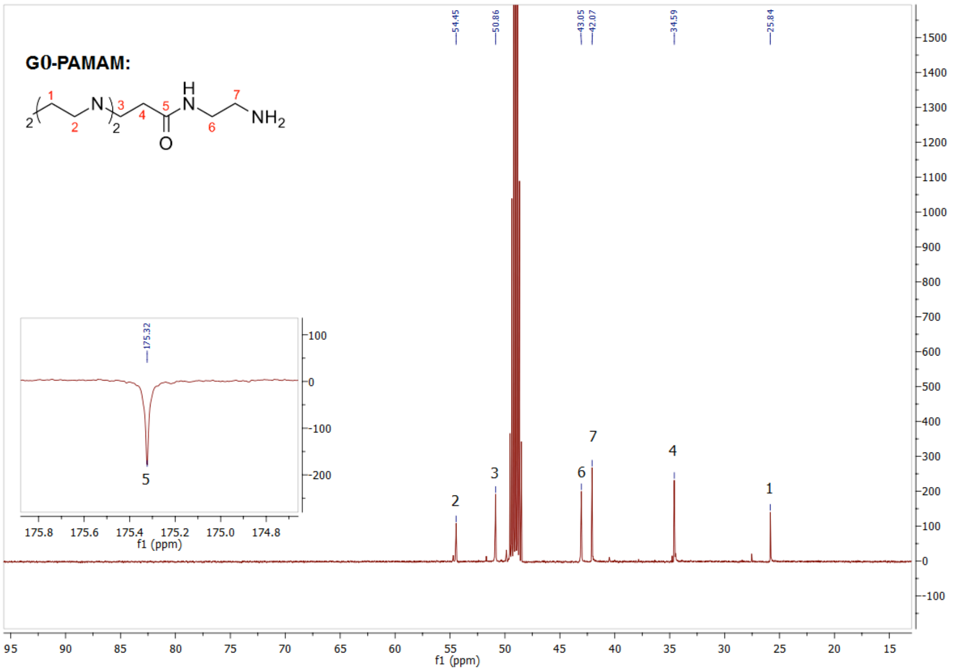

Supplementary Fig. 2. NMR Spectra of G0: DAB-PAMAM-$(NH_2)_4$ [2]. Top: ^{1}H-NMR (500 MHz, MeOD-d_4). Bottom: ^{13}C-NMR (125 MHz, MeOD-d_4).

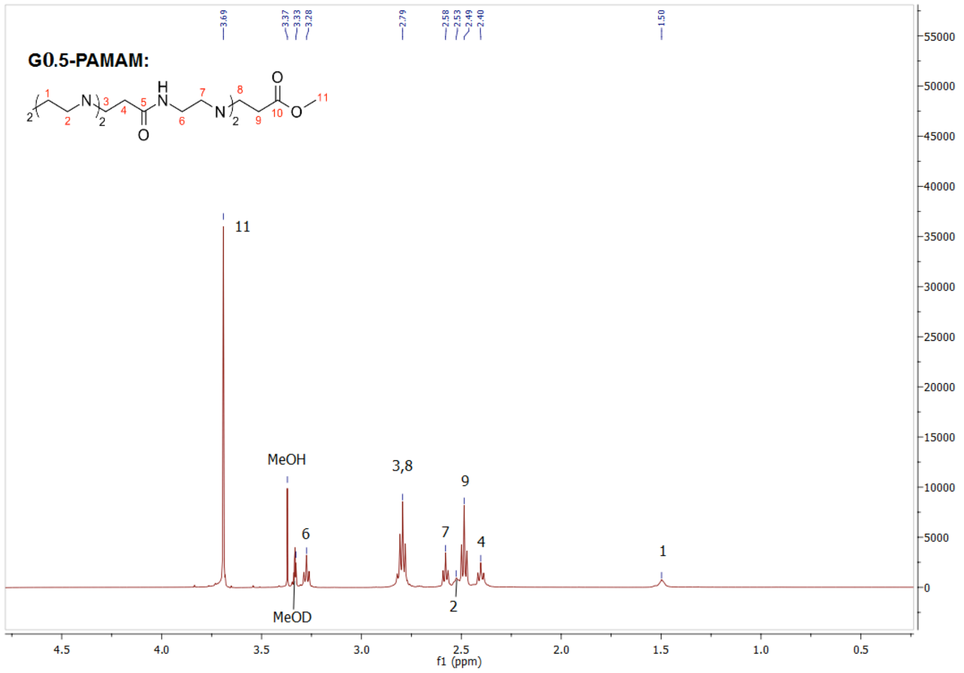

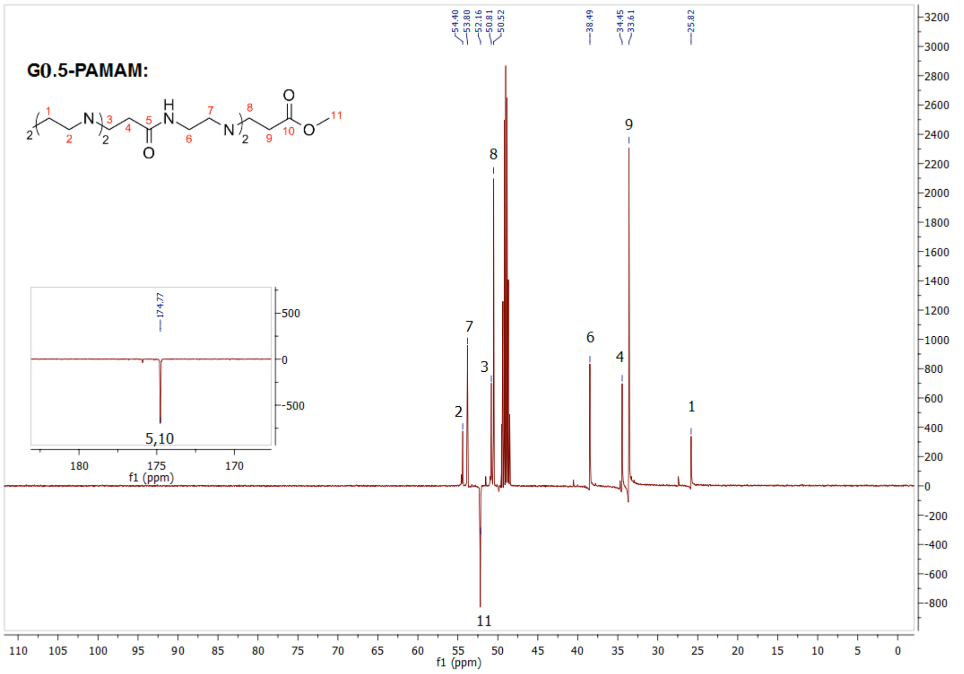

Supplementary Fig. 3. NMR spectra of G0.5: PAMAM(CO_2Me)$_8$ [3]. Top: ^{1}H-NMR (500 MHz, MeOD-d$_4$). Bottom: ^{13}C-NMR (125 MHz, MeOD-d$_4$).

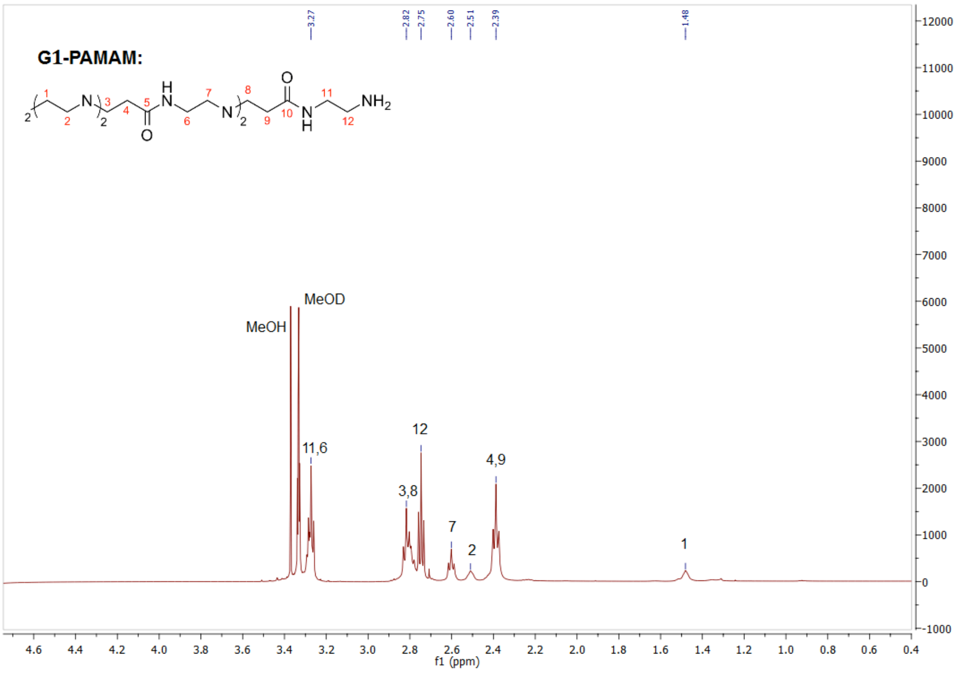

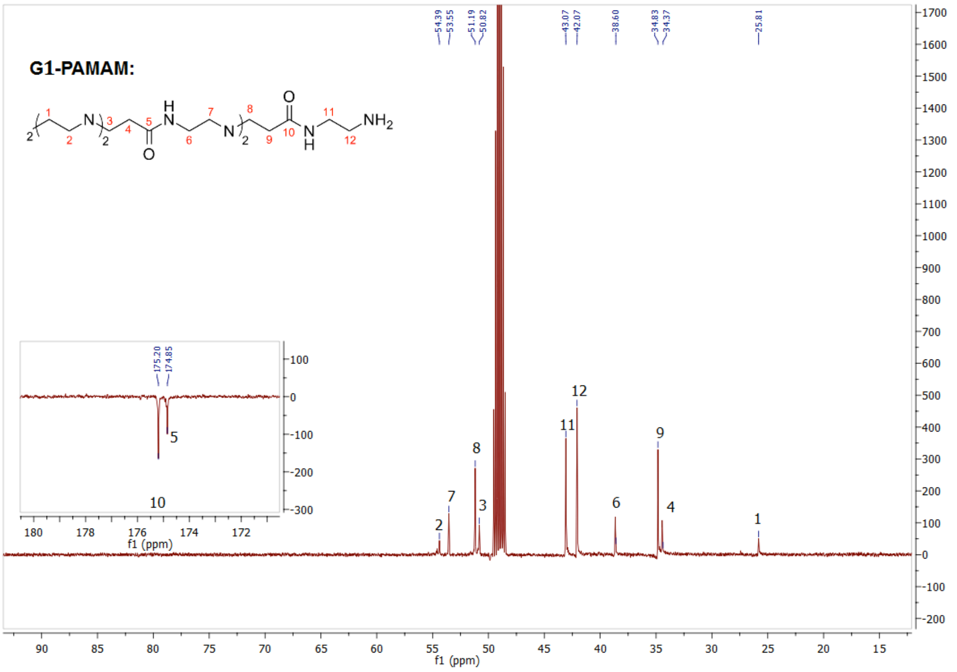

Supplementary Fig. 4. NMR spectra of G1: PAMAM$(NH_2)_8$ [4]. Top: ^{1}H-NMR (500 MHz, MeOD-d_4). Bottom: ^{13}C-NMR (125 MHz, MeOD-d_4).

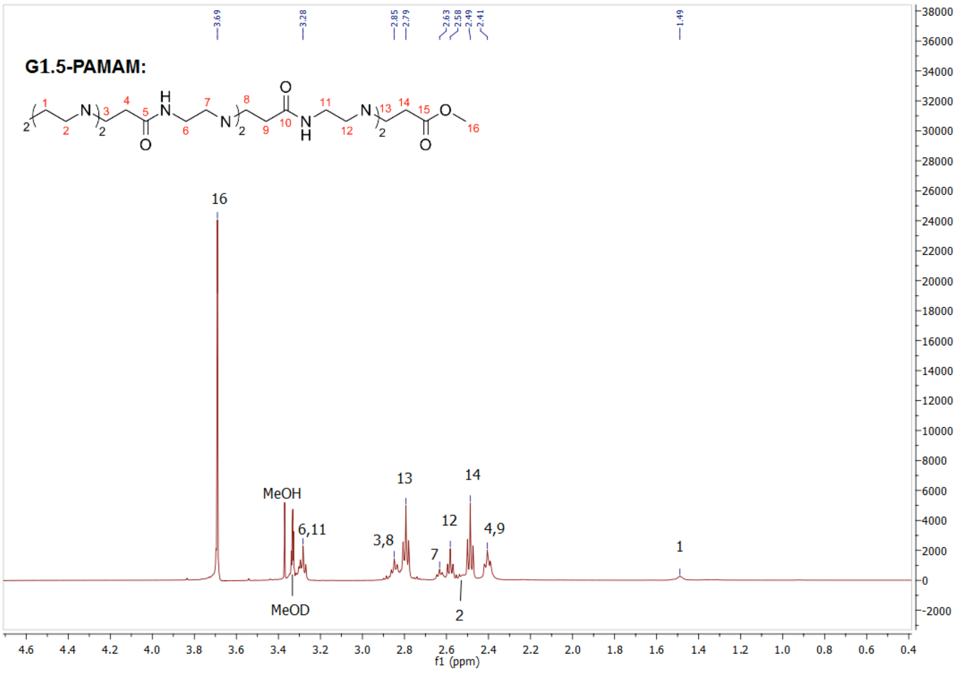

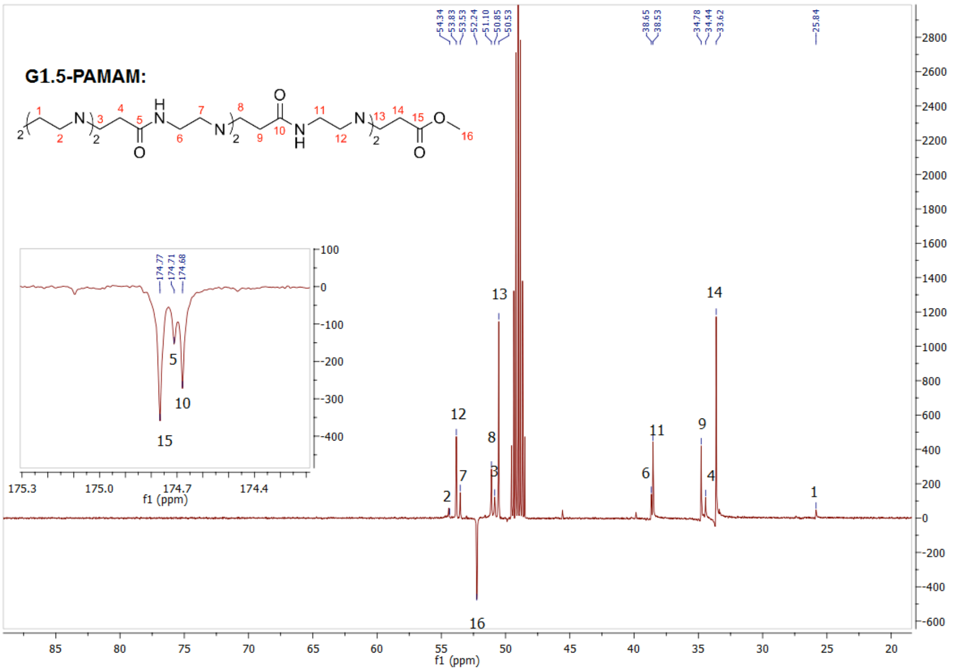

Supplementary Fig. 5. NMR spectra of G1.5: PAMAM(CO_2Me)$_{16}$ [5]. Top: ^{1}H-NMR (500 MHz, MeOD-d$_4$). Bottom: ^{13}C-NMR (125 MHz, MeOD-d$_4$).

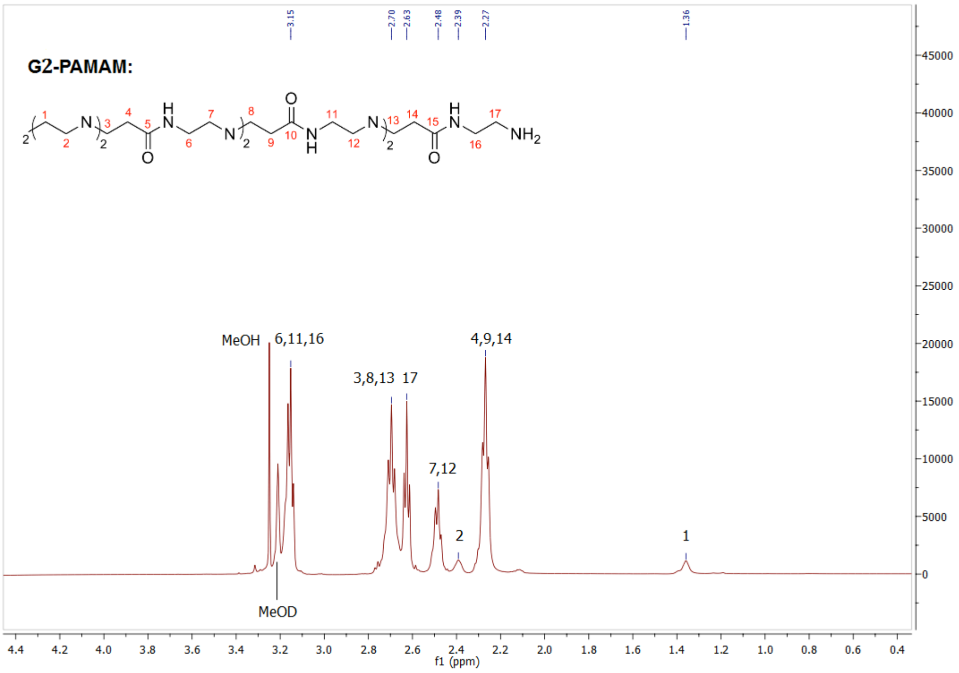

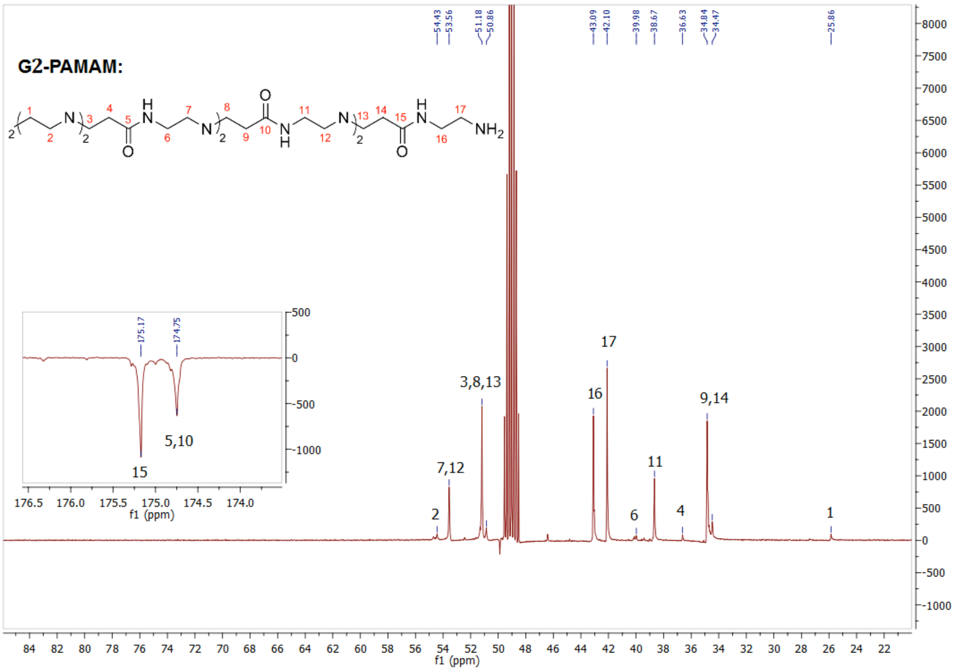

Supplementary Fig. 6. NMR spectra of G2: PAMAM$(NH_2)_{16}$ [6]. Top: ^{1}H-NMR (500 MHz, MeOD-d_4). Bottom: ^{13}C-NMR (125 MHz, MeOD-d_4).

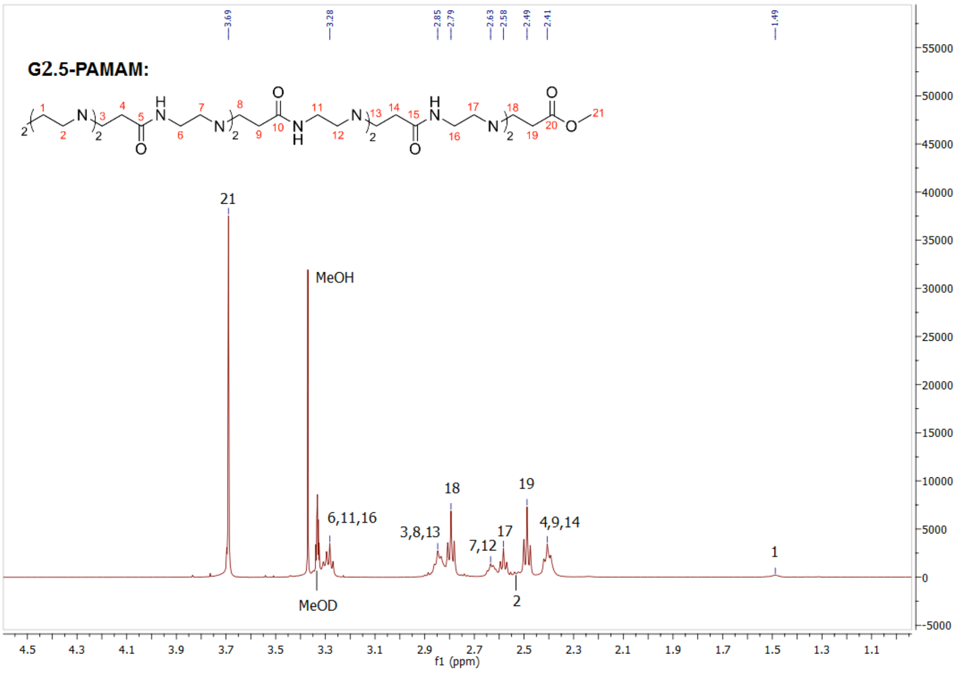

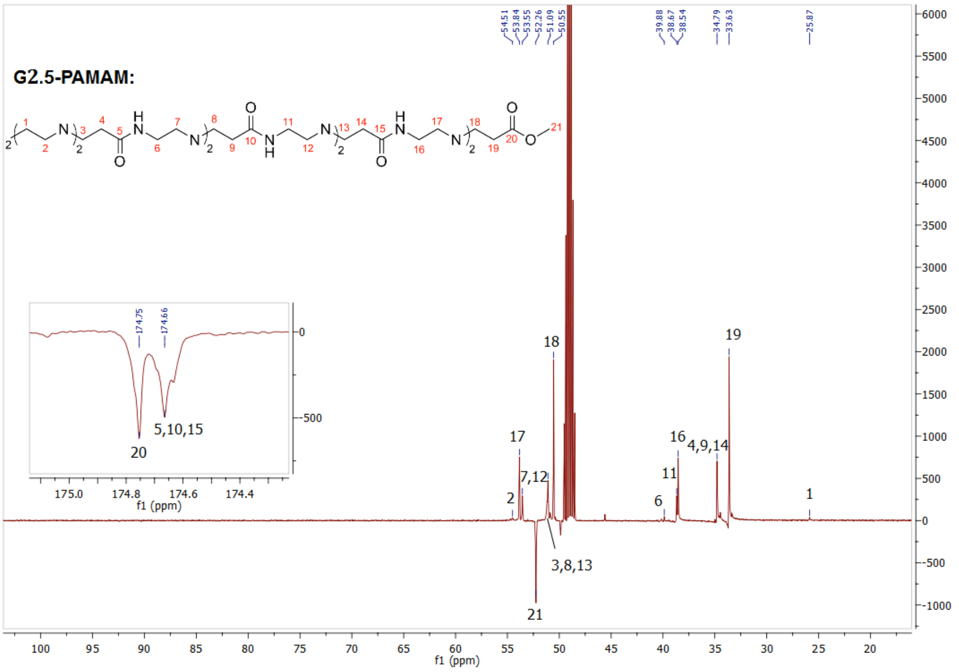

Supplementary Fig. 7. NMR spectra of G2.5: PAMAM(CO_2Me)$_{32}$ [7]. Top: ^{1}H-NMR (500 MHz, MeOD-d_4). Bottom: ^{13}C-NMR (125 MHz, MeOD-d_4).

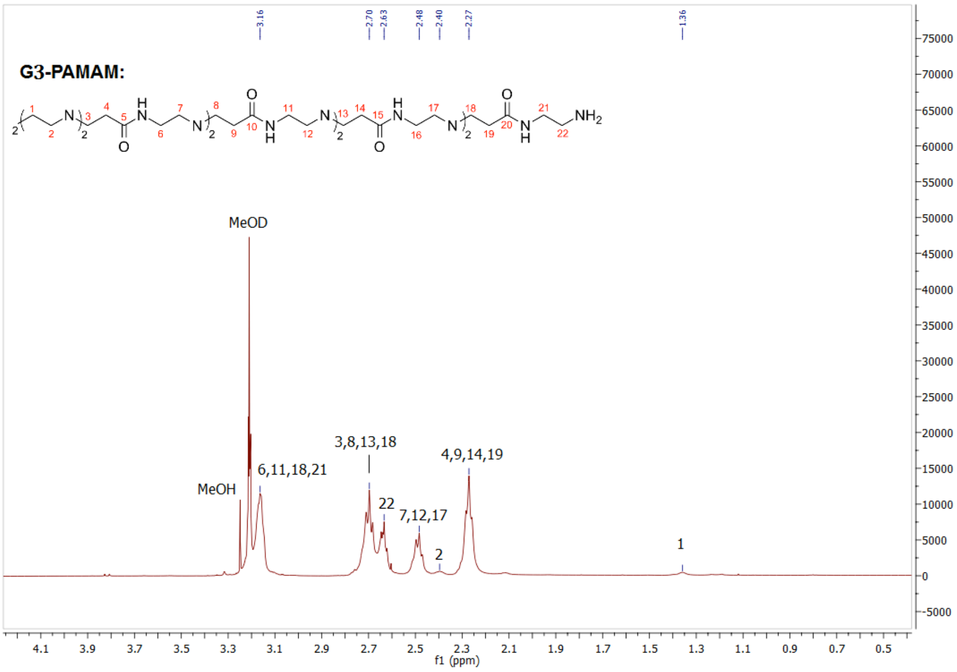

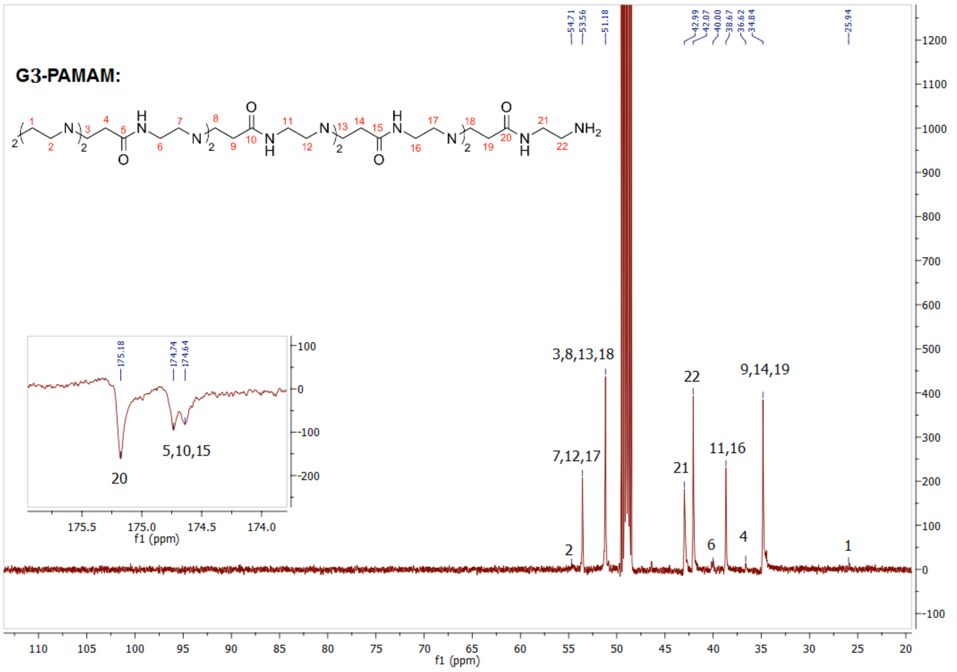

Supplementary Fig. 8. NMR spectra of G3: PAMAM$(NH_2)_{32}$ [8]. Top: ^{1}H-NMR (500 MHz, MeOD-d_4). Bottom: ^{13}C-NMR (125 MHz, MeOD-d_4).

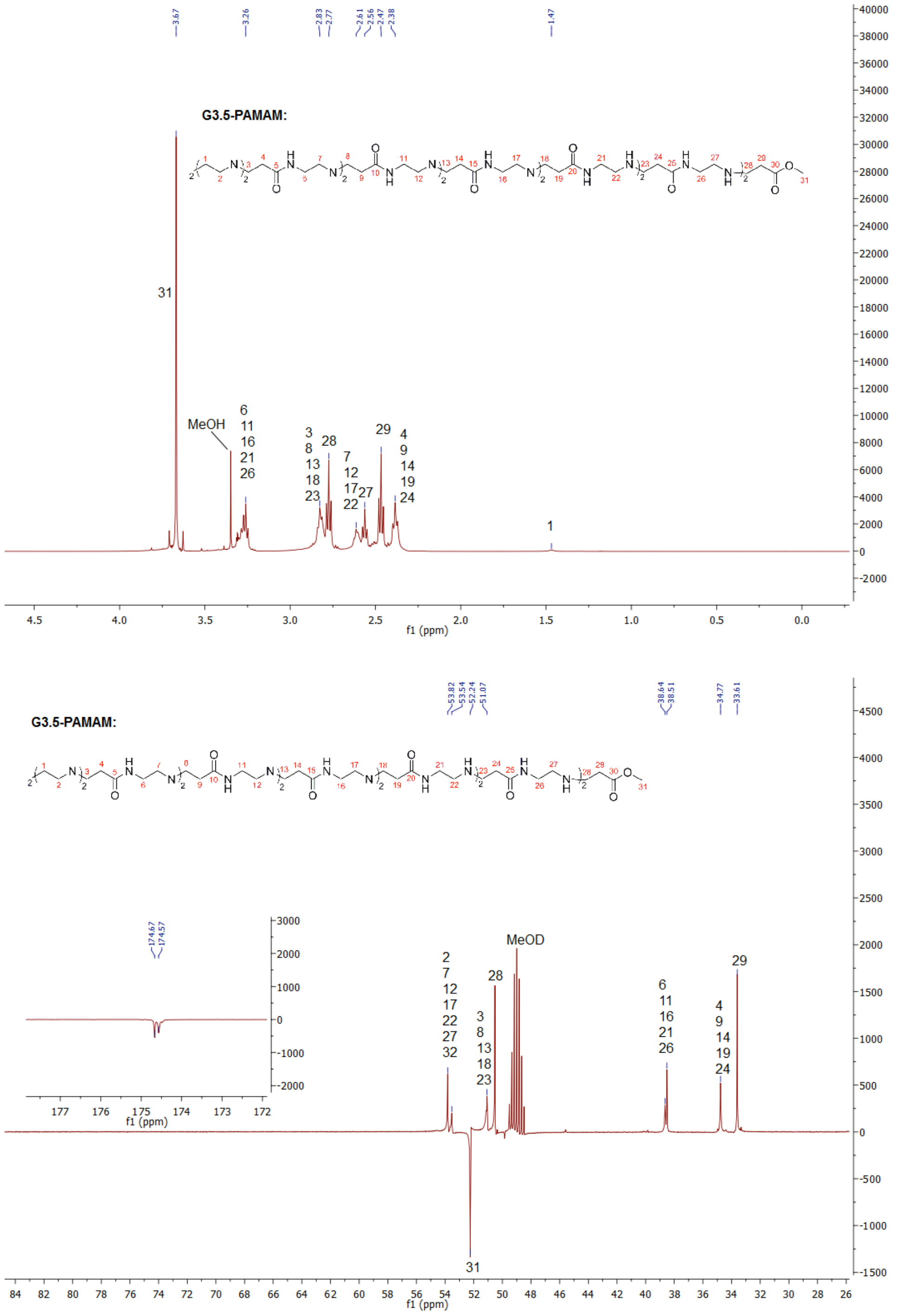

Supplementary Fig. 9. NMR spectra of G3.5: PAMAM$(CO_2Me)_{64}$ [9]. Top: ^{1}H-NMR (500 MHz, MeOD-d_4). Bottom: ^{13}C-NMR (125 MHz, MeOD-d_4).

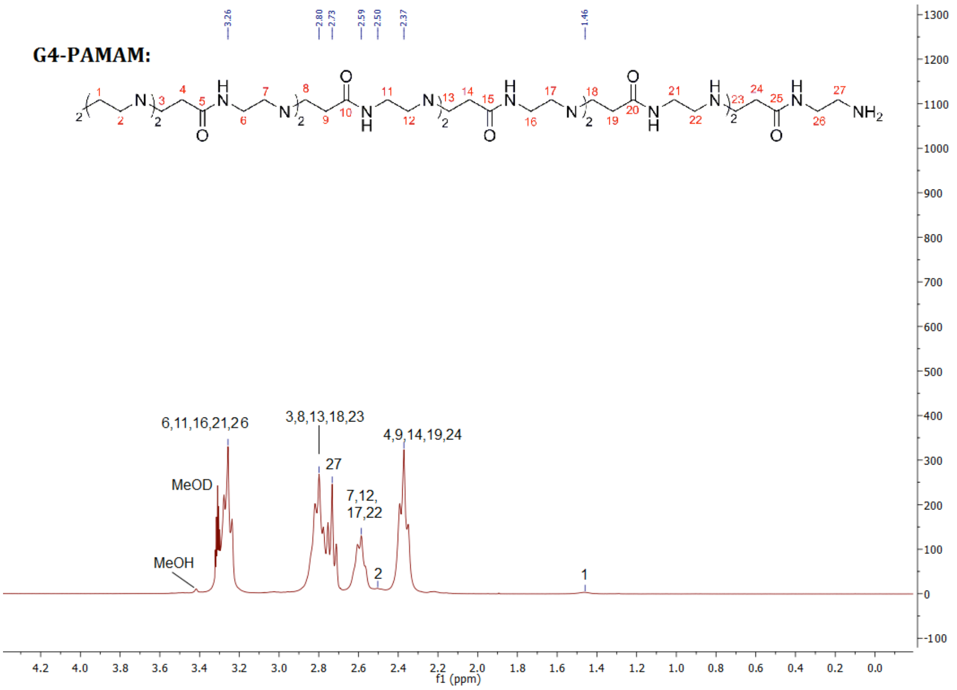

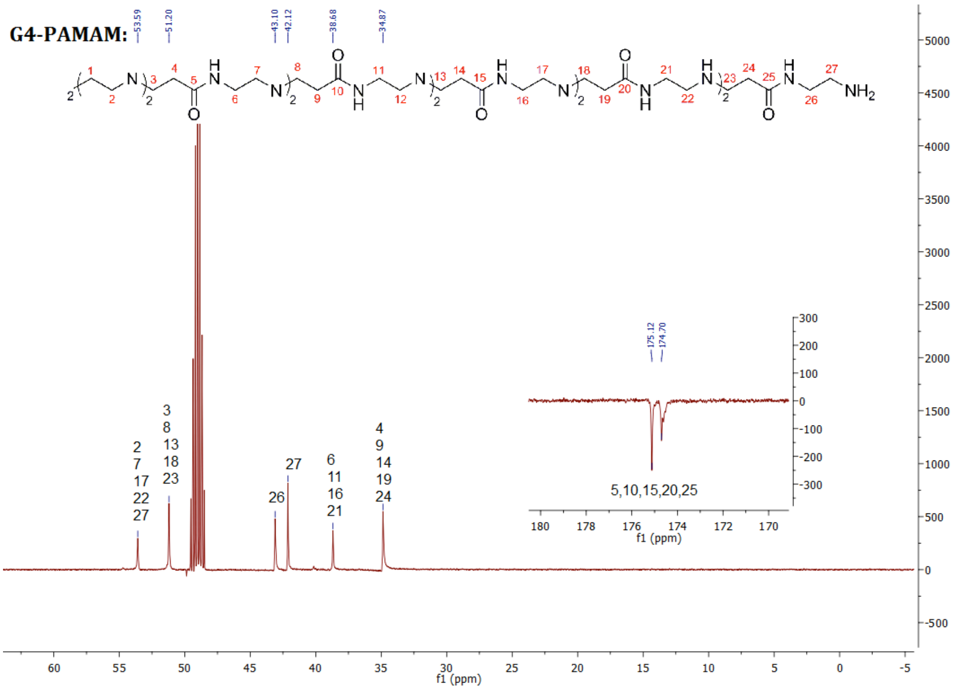

Supplementary Fig. 10. NMR spectra of G4: PAMAM$(NH_2)_{64}$ [10]. Top: ^{1}H-NMR (500 MHz, MeOD-d_4). Bottom: ^{13}C-NMR (125 MHz, MeOD-d_4).

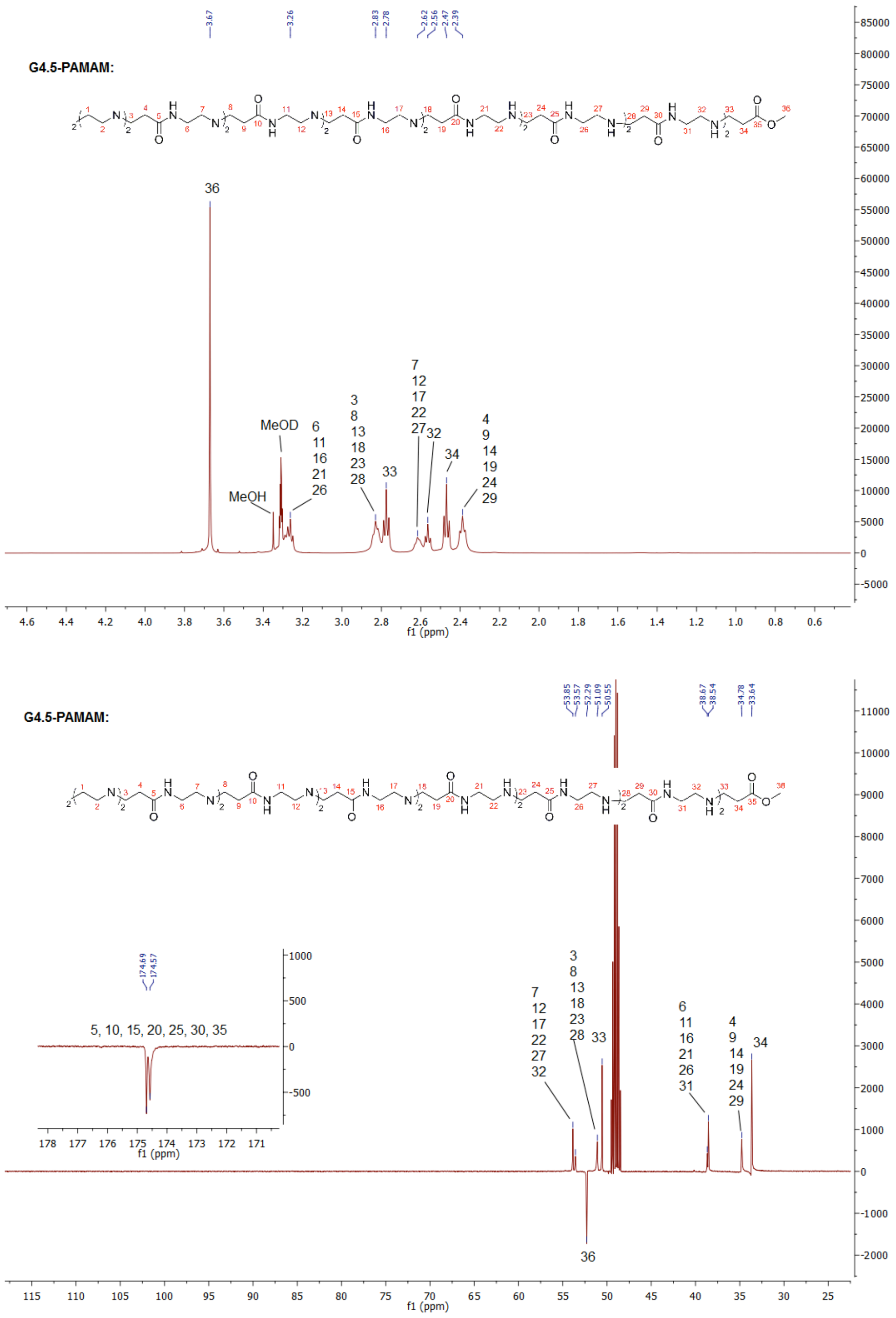

Supplementary Fig. 11. NMR spectra of G4.5: PAMAM(CO_2Me)$_{128}$ [11]. Top: ^{1}H-NMR (500 MHz, MeOD-d_4). Bottom: ^{13}C-NMR (125 MHz, MeOD-d_4).

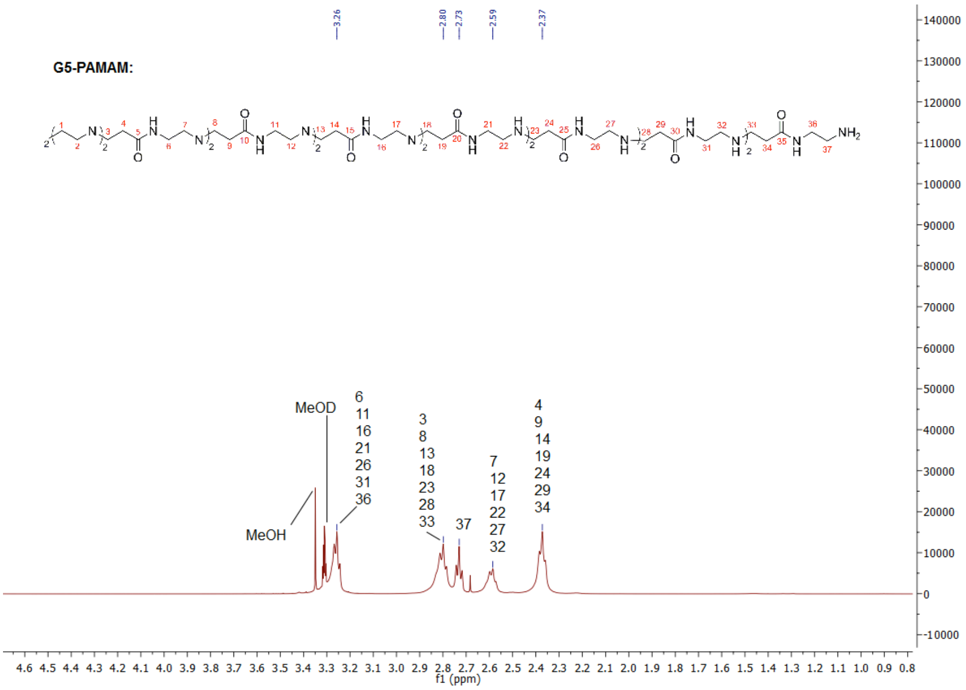

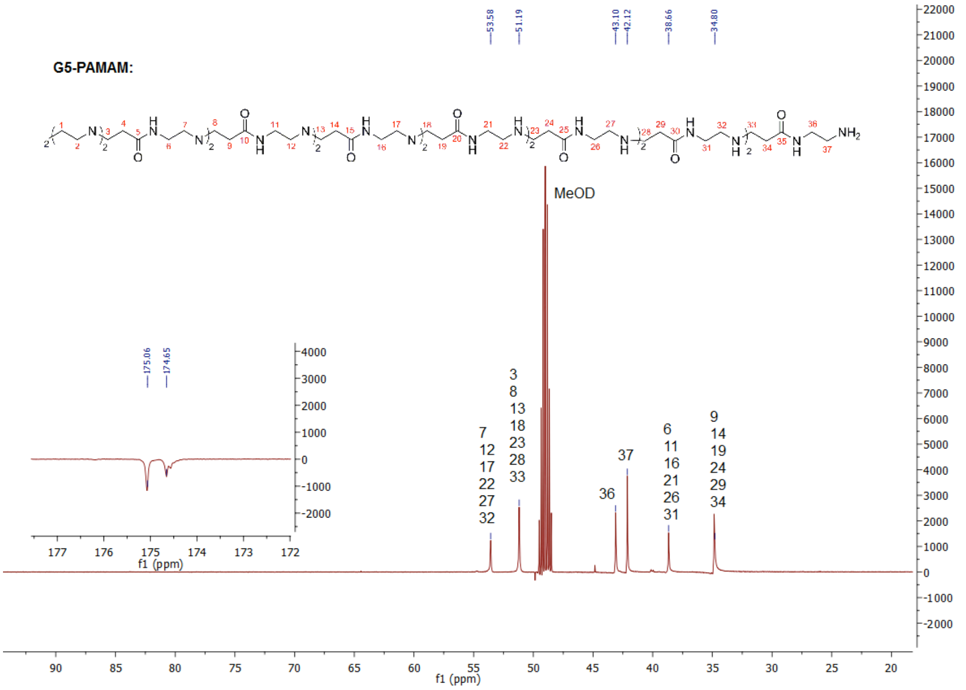

Supplementary Fig. 12. NMR spectra of G5: PAMAM$(NH_2)_{128}$ [12]. Top: ^{1}H-NMR (500 MHz, MeOD-d_4). Bottom: ^{13}C-NMR (125 MHz, MeOD-d_4).

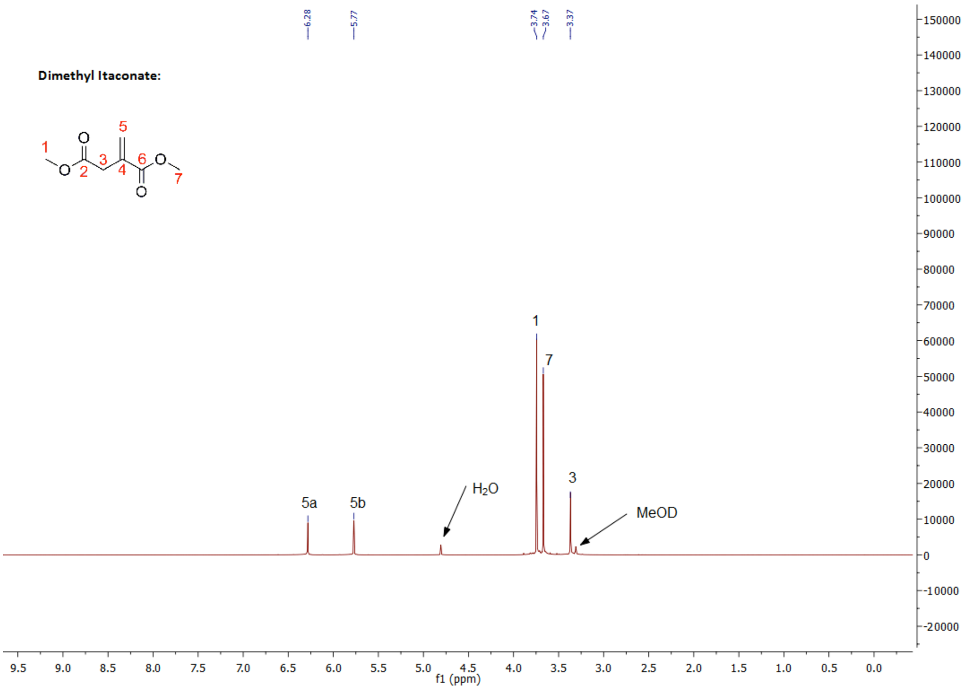

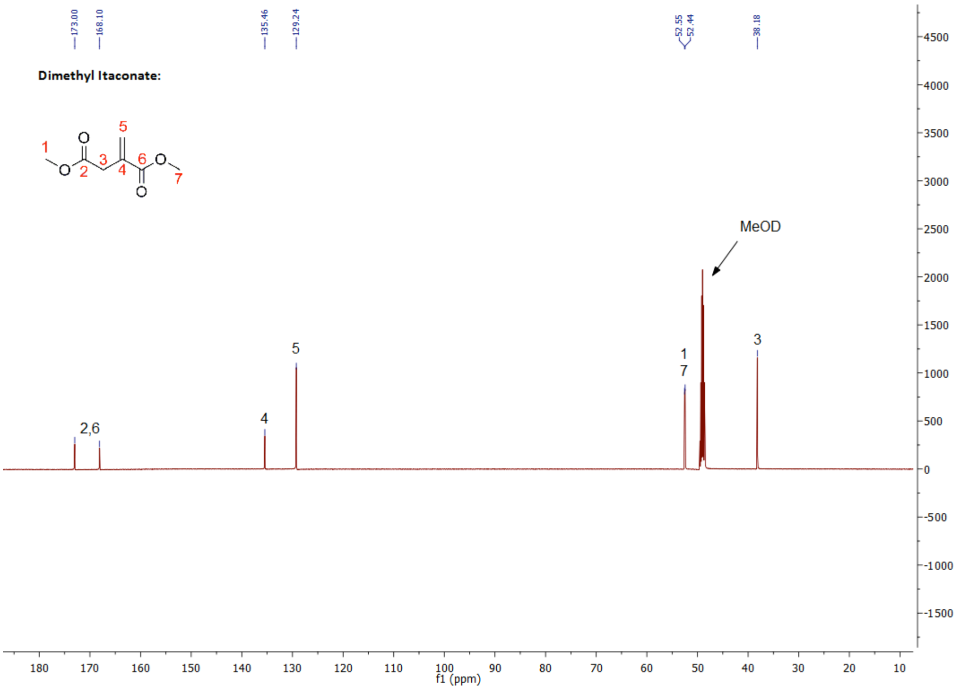

Supplementary Fig. 13. NMR spectra of dimethyl itaconate [13]. Top: ^{1}H-NMR (500 MHz, MeOD-d$_4$). Bottom: ^{13}C-NMR (125 MHz, MeOD-d$_4$).

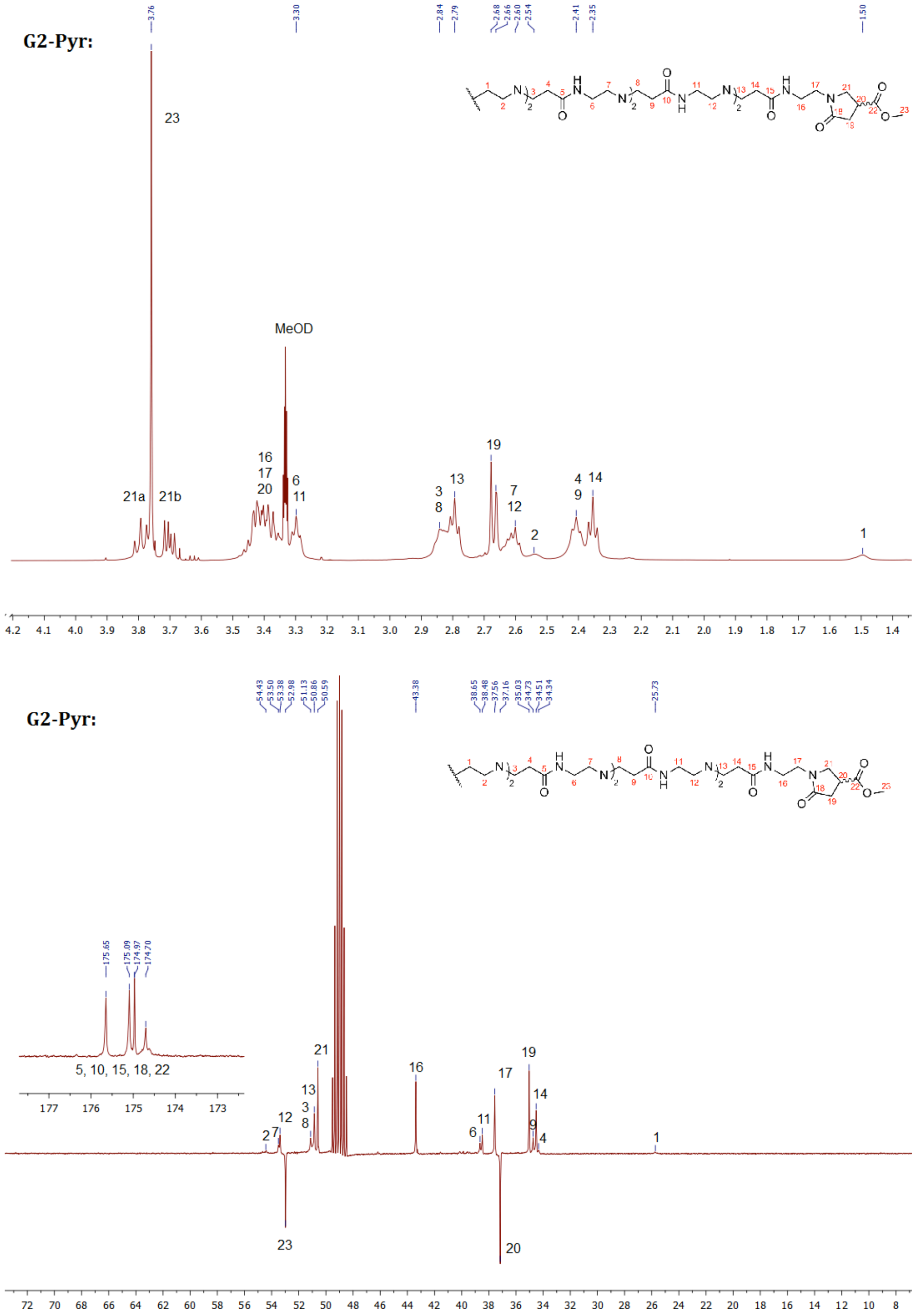

Supplementary Fig. 14. NMR spectra of G2: PAMAM(Pyr)$_{16}$ [16]. Top: ^{1}H-NMR (500 MHz, MeOD-d$_4$). Bottom: ^{13}C-NMR (125 MHz, MeOD-d$_4$).

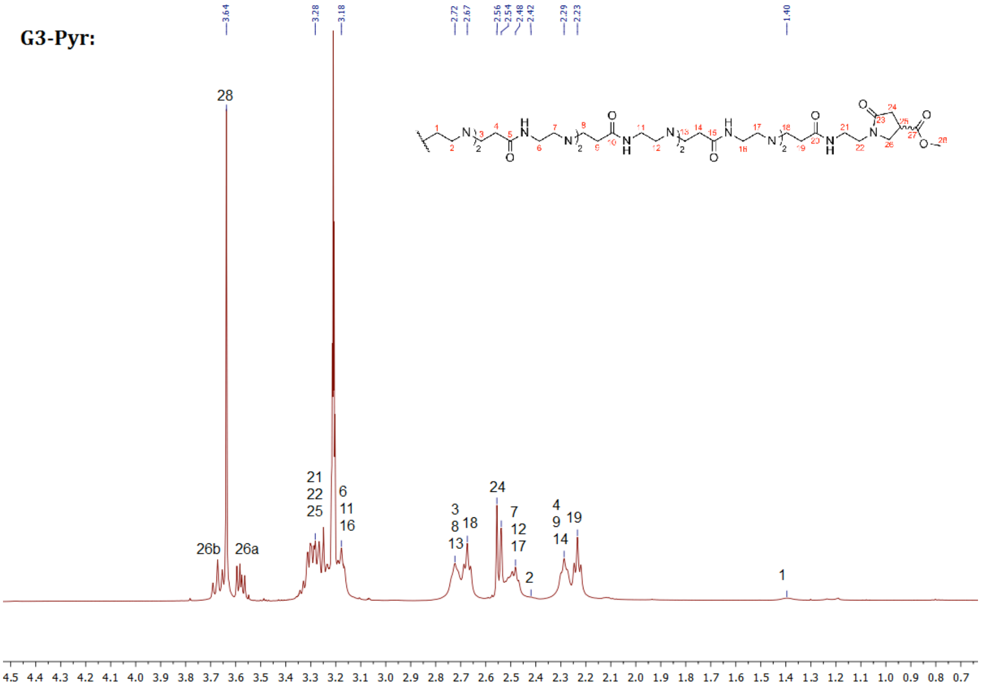

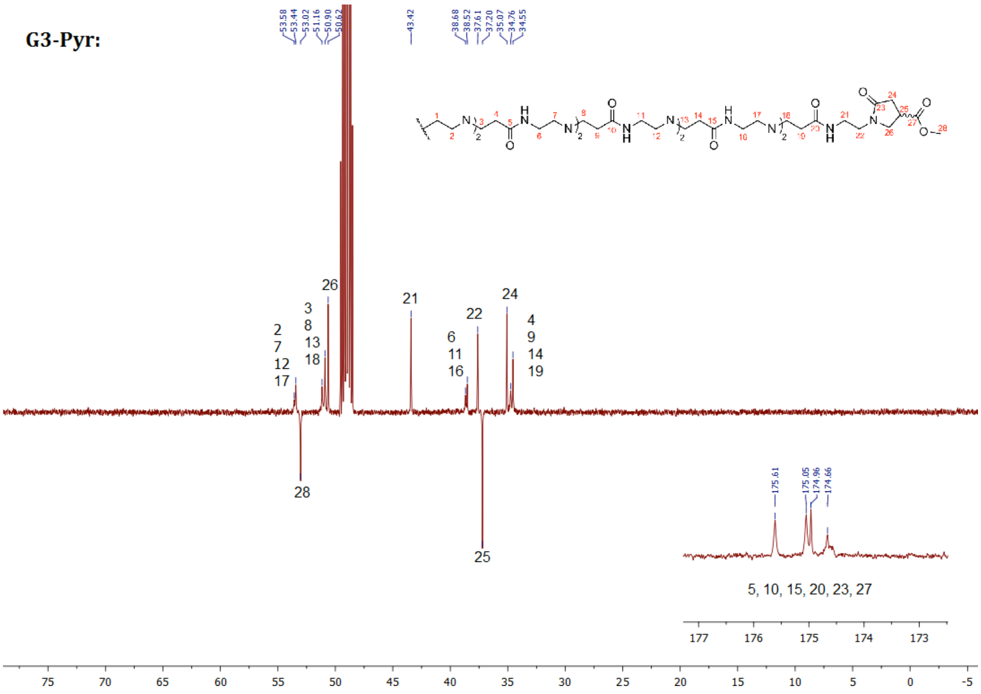

Supplementary Fig. 15. NMR spectra of G3: PAMAM(Pyr)$_{32}$ [17]. Top: ^{1}H-NMR (500 MHz, MeOD-d$_4$). Bottom: ^{13}C-NMR (125 MHz, MeOD-d$_4$).

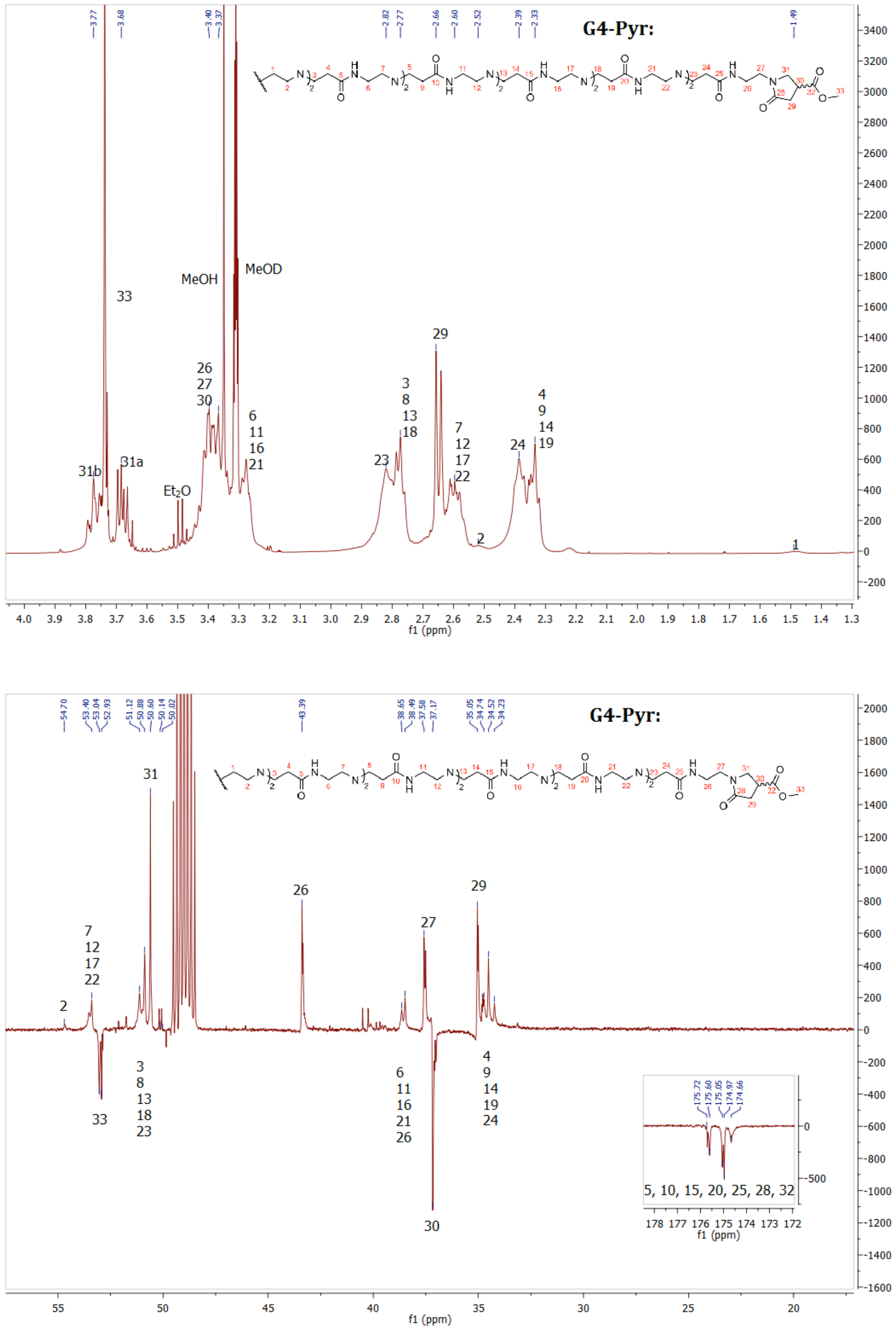

Supplementary Fig. 16. NMR spectra of G4: PAMAM(Pyr)$_{64}$ [18]. Top: ^{1}H-NMR (500 MHz, MeOD-d$_4$). Bottom: ^{13}C-NMR (125 MHz, MeOD-d$_4$).

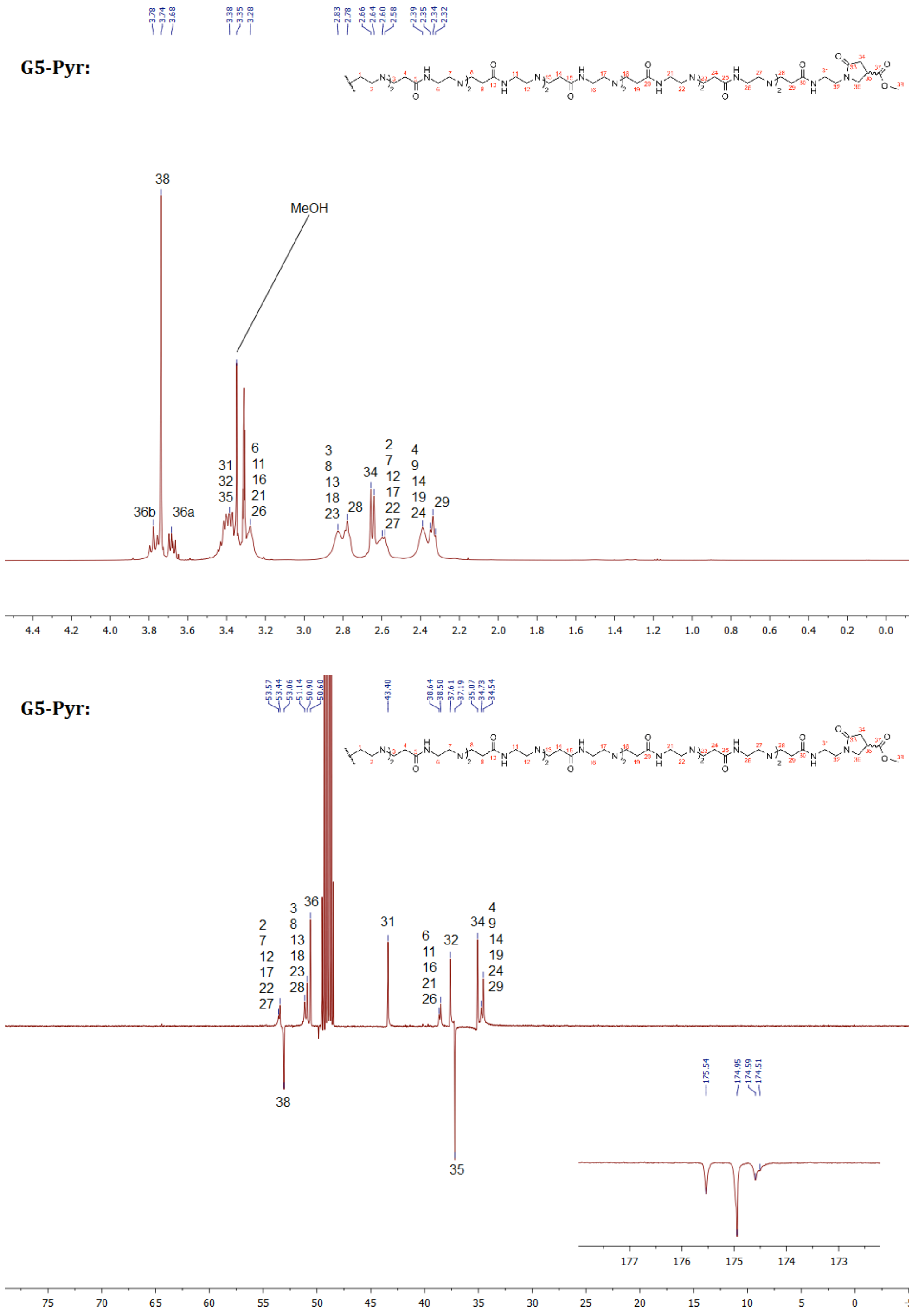

Supplementary Fig. 17. NMR spectra of G5: PAMAM(Pyr)$_{128}$ [19]. Top: ^{1}H-NMR (500 MHz, MeOD-d$_4$). Bottom: ^{13}C-NMR (125 MHz, MeOD-d$_4$).

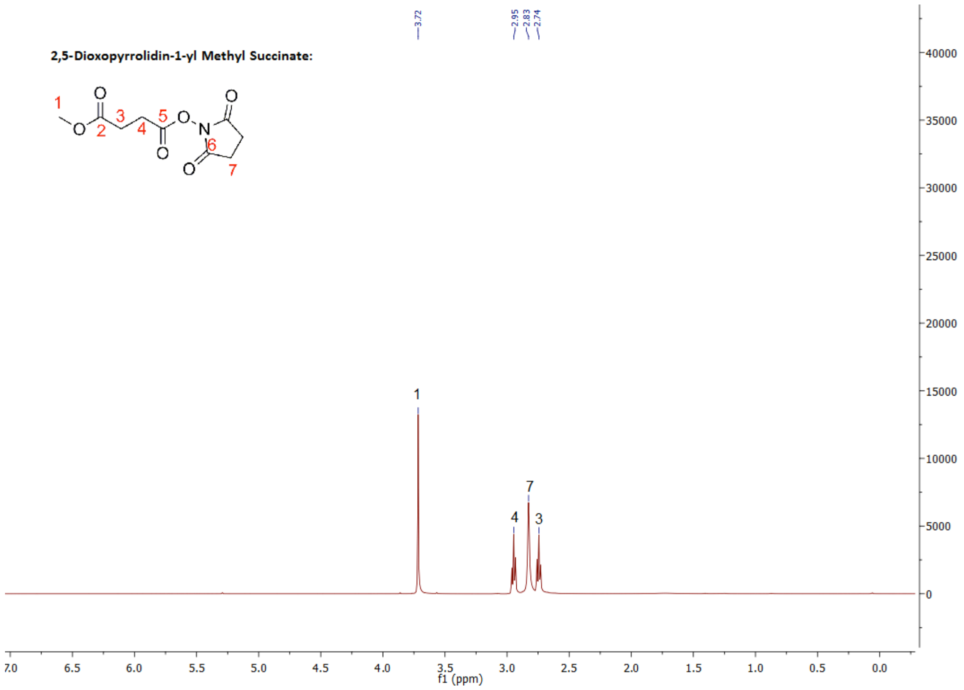

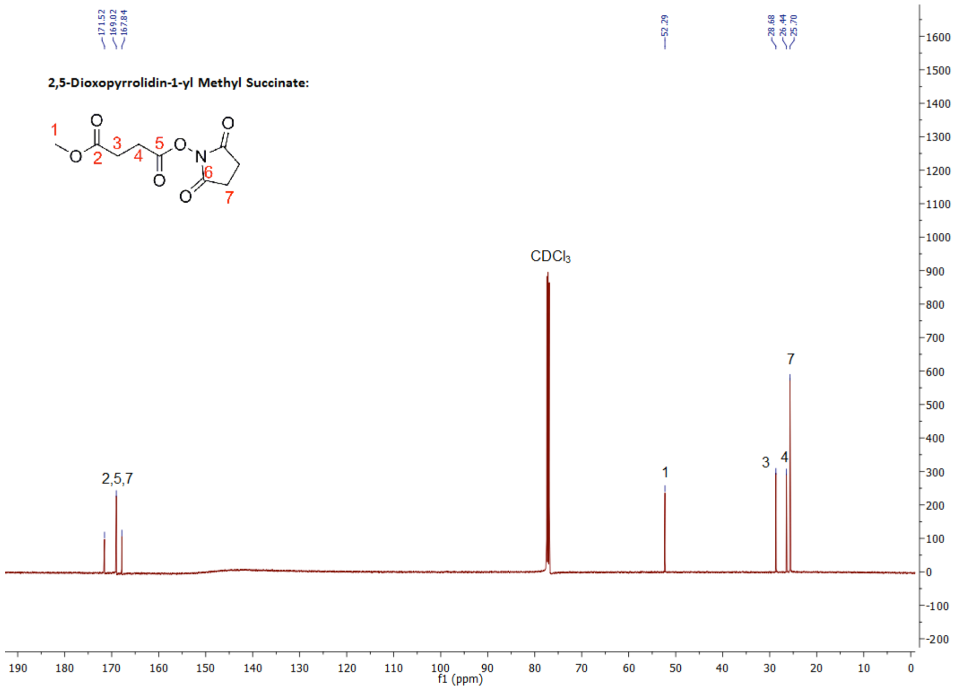

Supplementary Fig. 18. NMR spectra of 2,5-dioxopyrrolidin-1-yl methyl succinate [20]. Top: ^{1}H-NMR (500 MHz, $CDCl_3$). Bottom: ^{13}C-NMR (125 MHz, $CDCl_3$).

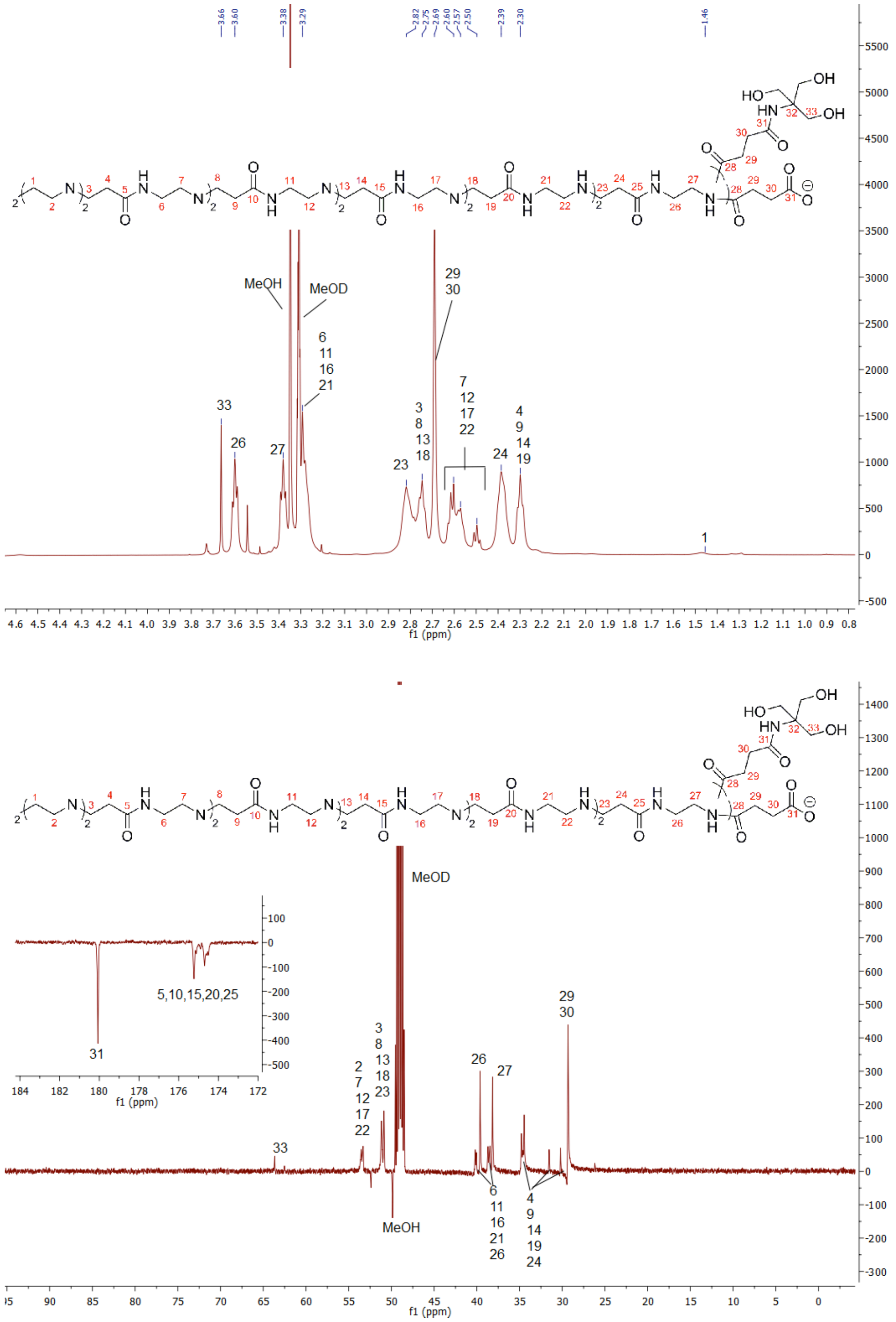

Supplementary Fig. 19. NMR spectra of G4: PAMAM-(COOH)$_{58}$(Tris)$_{6}$ [21]. Top: ^{1}H-NMR (500 MHz, MeOD-d$_{4}$). Bottom: ^{13}C-NMR (125 MHz, MeOD-d$_{4}$).

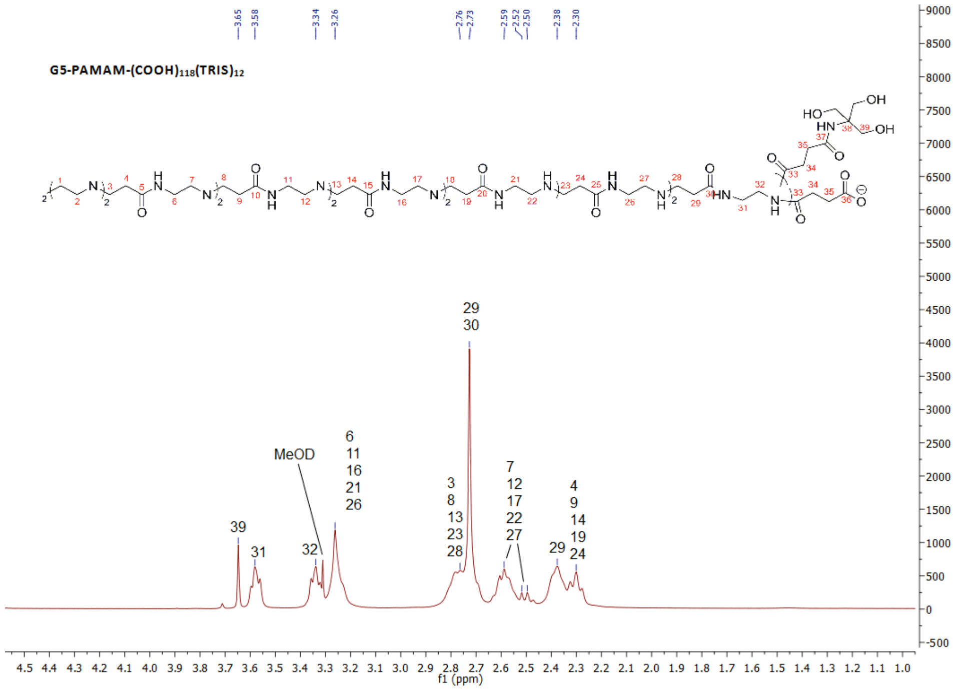

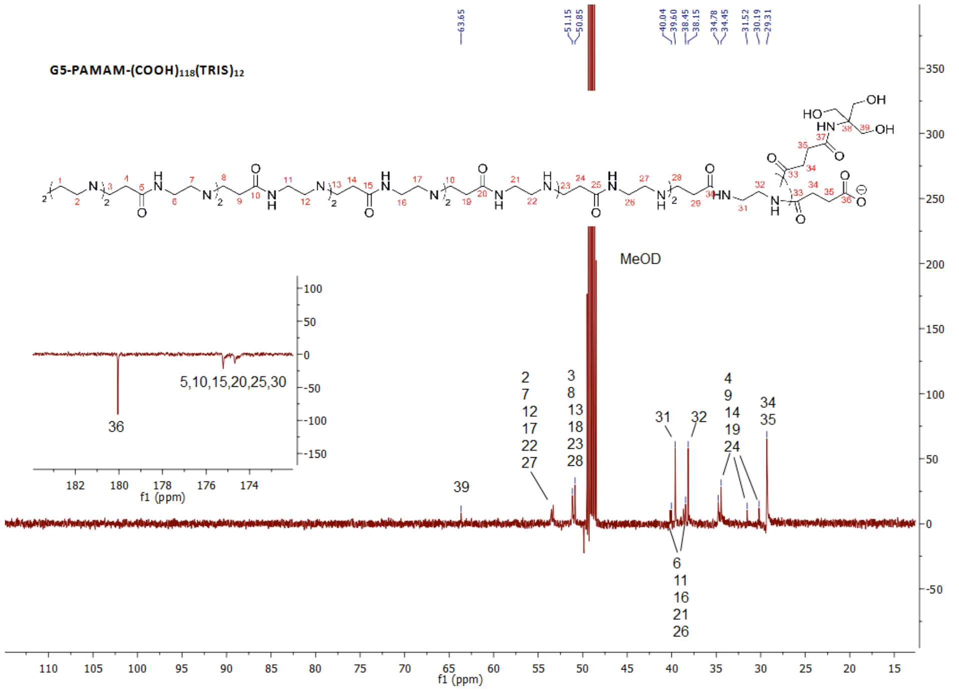

Supplementary Fig. 20. NMR spectra of G5: PAMAM-$(COOH)_{118}(Tris)_{12}$ [21]. Top: ^{1}H-NMR (500 MHz, MeOD-d_4). Bottom: ^{13}C-NMR (125 MHz, MeOD-d_4).

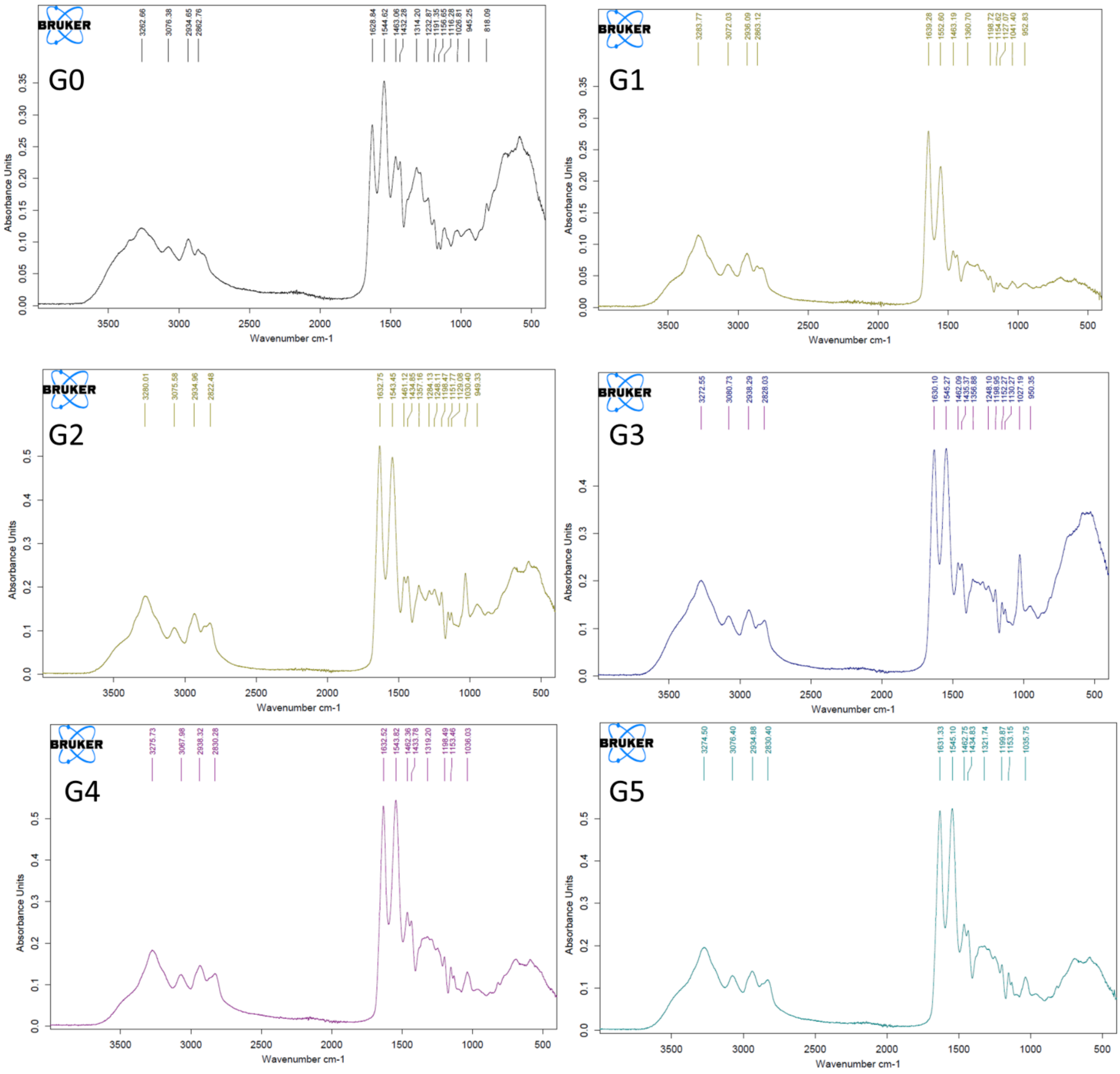

Supplementary Fig. 21. FT-IR-spectra (absorbance) of G0–G5 amine terminated PAMAM dendrimers.

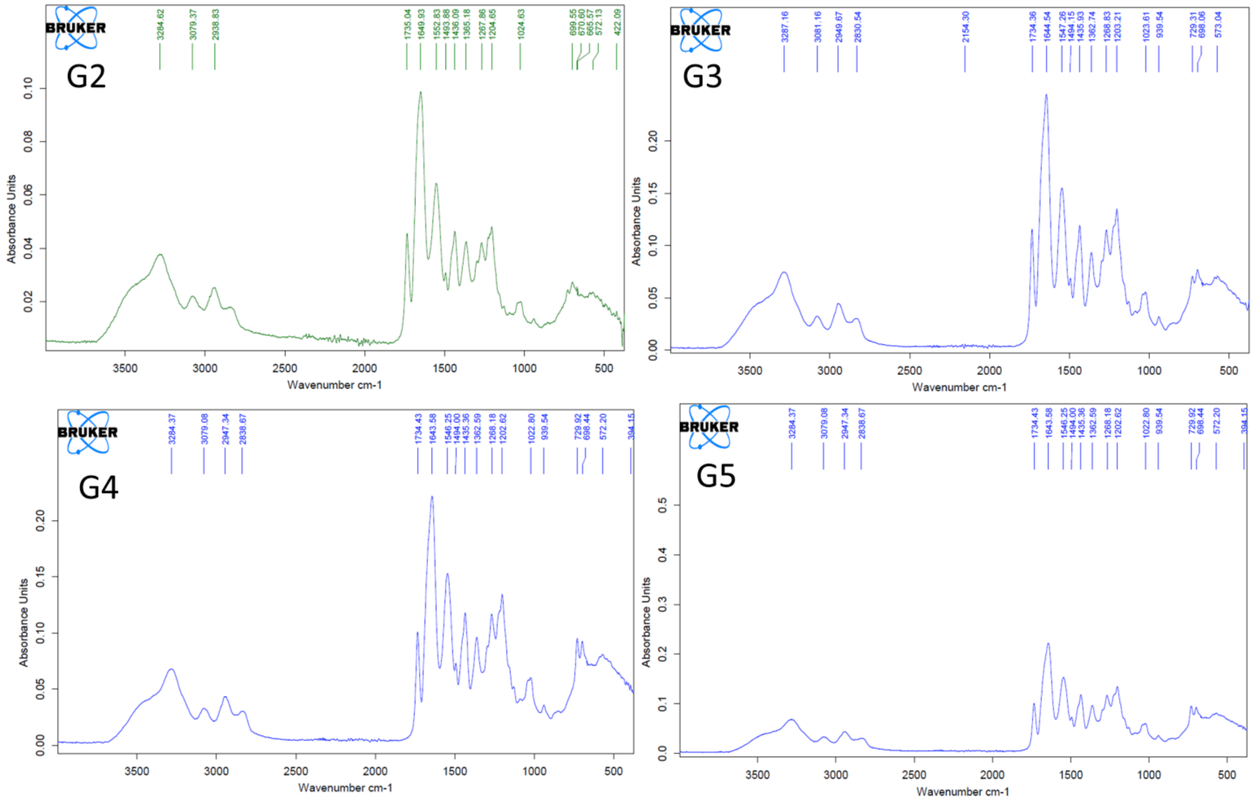

Supplementary Fig. 22. FT-IR-spectra (absorbance) of G2–G5 1-(4-carbomethoxypyrrolidone) terminated PAMAM dendrimers.

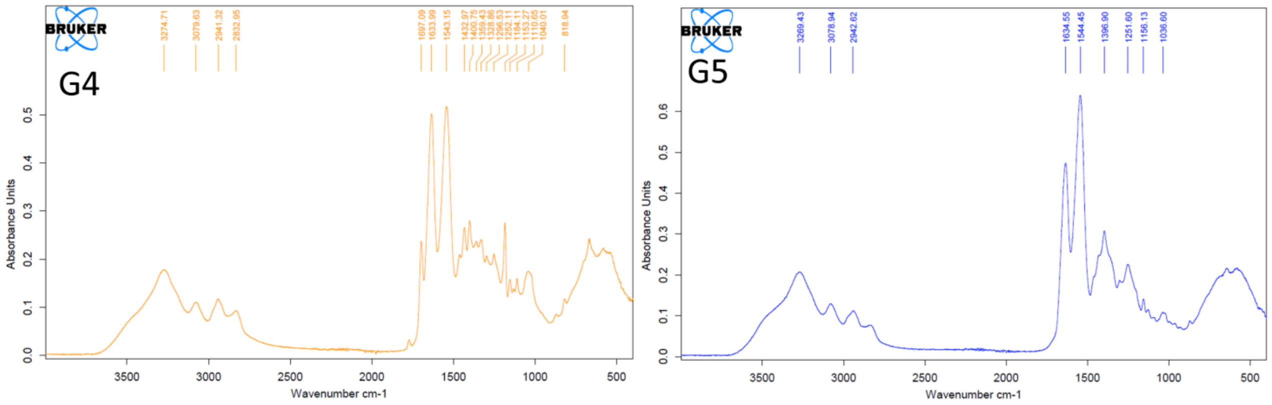

Supplementary Fig. 23. FT-IR-Spectra (absorbance) of G4 and G5 carboxy-Tris terminated PAMAM dendrimers.

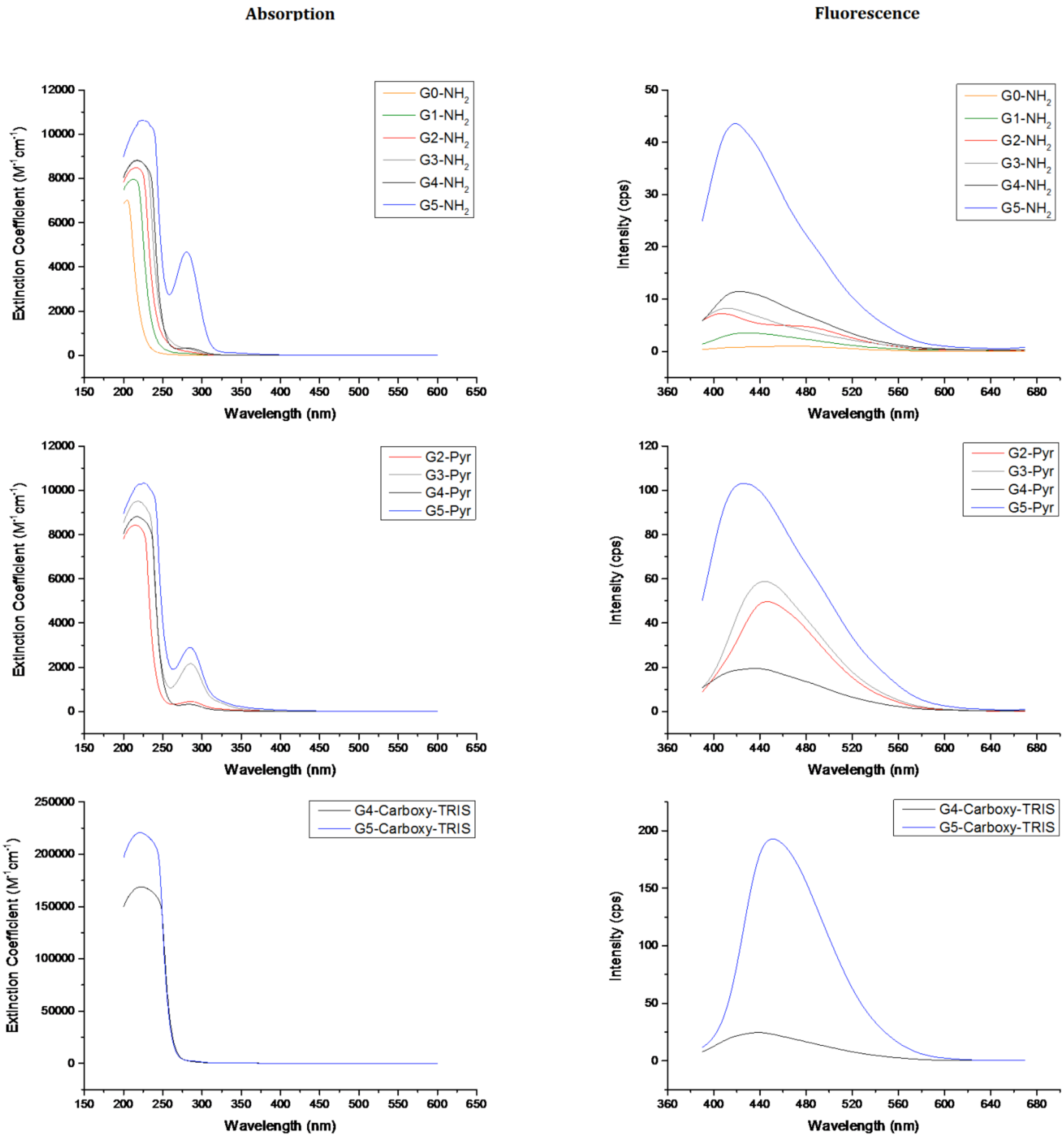

Supplementary Fig. 24. UV/Vis (absorption) and fluorescence (excitation at λ = 360 nm) spectra of G0–G5 amine terminated PAMAM dendrimers, G2–G5 1-(4-carbomethoxypyrrolidone) terminated PAMAM dendrimers, and G4 and G5 carboxy-Tris terminated PAMAM dendrimers.

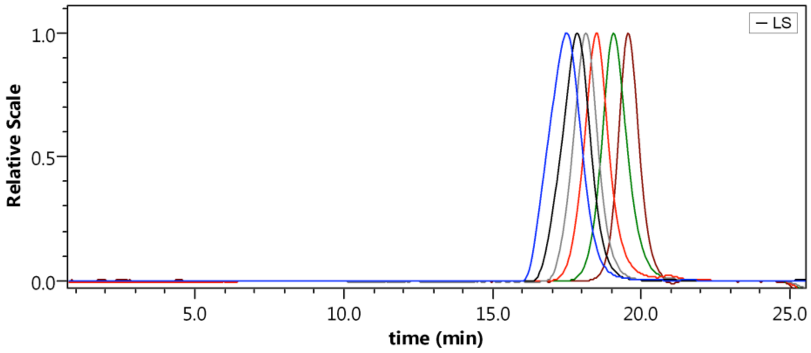

Supplementary Fig. 25. SEC-MALS chromatogram of G0–G5 amine terminated PAMAM dendrimers. G0 = brown, G1 = green, G2 = red, G3 = grey, G4 = black, and G5 = blue.

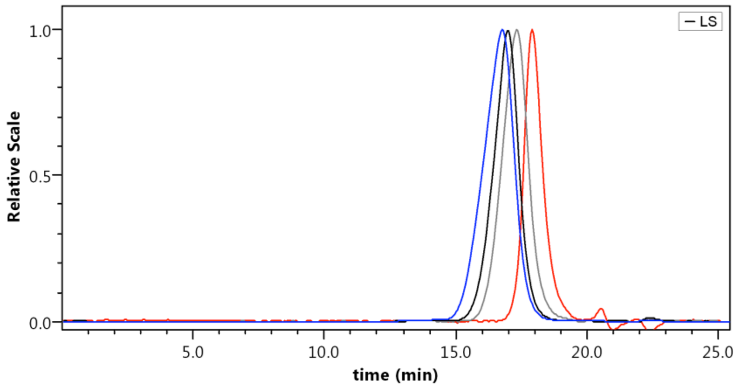

Supplementary Fig. 26. SEC-MALS chromatogram of G2–G5 1-(4-carbomethoxypyrrolidone) terminated PAMAM dendrimers. G2 = red, G3 = grey, G4 = black, and G5 = blue.

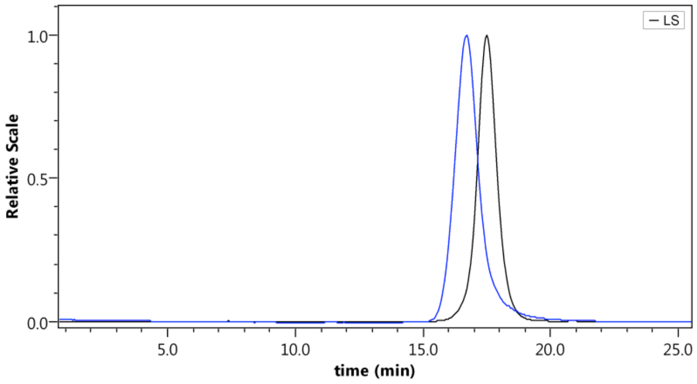

Supplementary Fig. 27. SEC-MALS chromatogram of G4 and G5 carboxy-Tris terminated PAMAM dendrimers. G4 = black and G5 = blue.

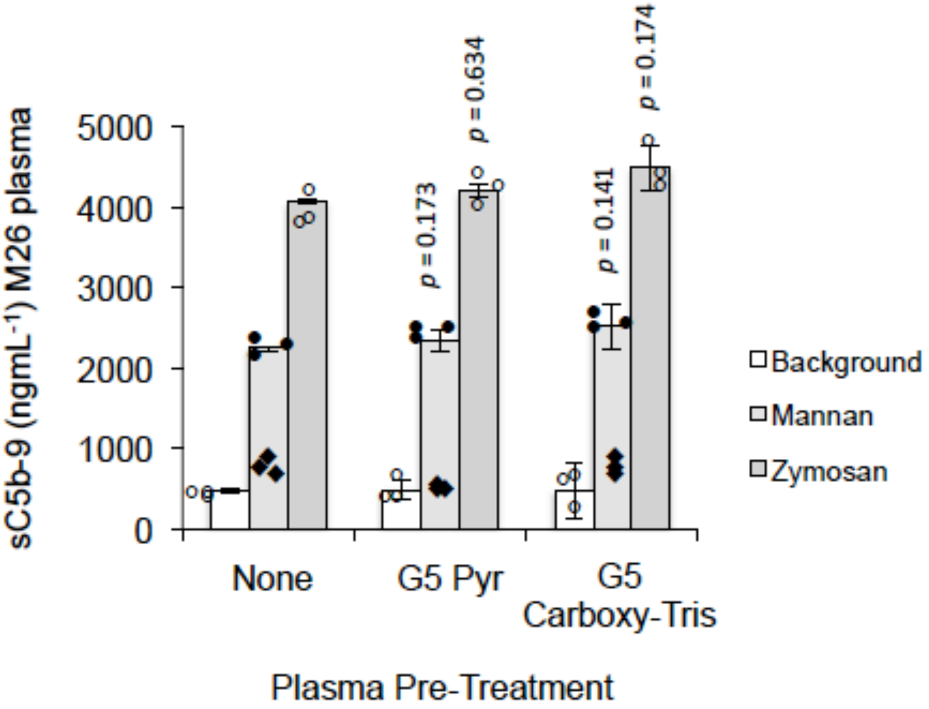

Supplementary Fig. 28. Plasma (M26) pre-treatment (15 min, 37 °C) with G5 pyrrolidone (G5 Pyr)- and carboxy$_{116}$-Tris$_{12}$ (G5 Carboxy-Tris) dendrimers (final dendrimer concentration in plasma = 0.132 mM) has no effect on complement functionality. Dendrimer pre-treated plasma exhibit complement activation (sC5b-9 elevation) in mannan-coated wells with levels comparable to un-treated plasma (no treatment). Zymosan (750 μgmL^{-1}) also activates complement in dendrimer pre-treated plasma with levels comparable to untreated plasma. Experimental details are described in Methods. The "Background" column refers to plasma sCb5-9 background levels for comparison with zymosan treatment. The black rhombus dots in mannan columns refer to plasma sC5b-9 background levels in the presence of 10 mM EDTA (background) in mannan-coated wells. Bars show mean ± s.d. of three technical replicates. Each experiment was done in triplicate samples. The statistical analysis is explained in the Methods and *p* values (unpaired, two-sided) are compared with respective background values.

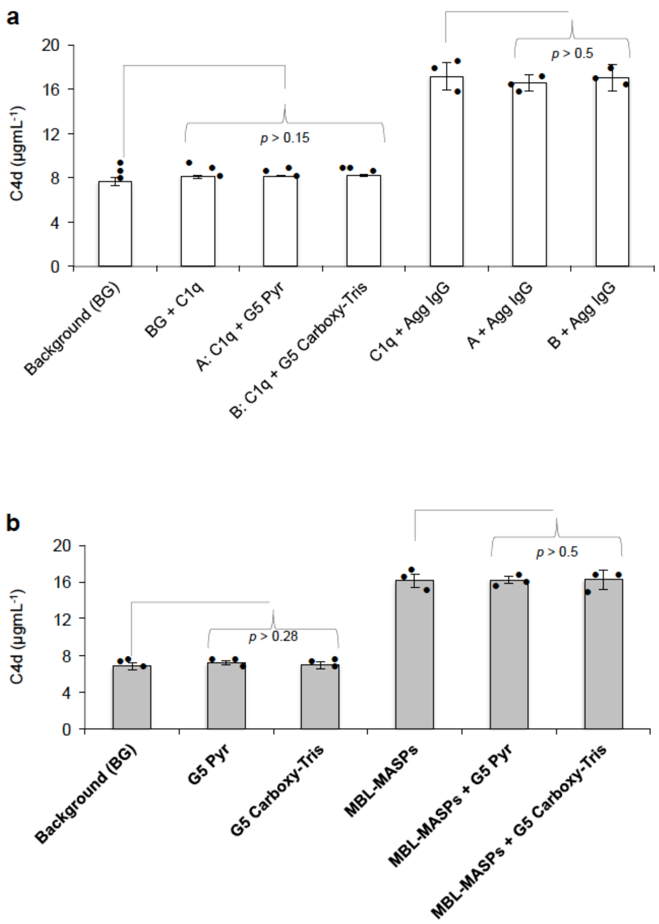

Supplementary Fig. 29. The effect of G5 pyrrolidone (G5 Pyr) and carboxy$_{116}$-Tris$_{12}$ (G5 Carboxy-Tris) dendrimers on substrate-mediated generation of C4d-conatining activation fragments of C4 (C4b, iC4b, and C4d) (collectively referred to as C4d) in a C1q-depleted human serum **(a)** and MBL-deficient M48 human plasma **(b)**. In (a) the substrate was aggregated IgG and the experiments were performed in Eppendorf tubes as described in Methods. In (b) the substrate were mannan-coated wells (for experimental procedures see Methods). Dendrimer concentration = 0.132 mM; C1q = 180 μgmL^{-1}; and MBL/MASPs = 5 μgmL^{-1} MBL. Bars show mean ± s.d. of three technical replicates. Each experiment was done in triplicate samples. Differences between groups were examined using ANOVA followed by multiple comparisons with two-tailed Student-Newmann-Keul test.

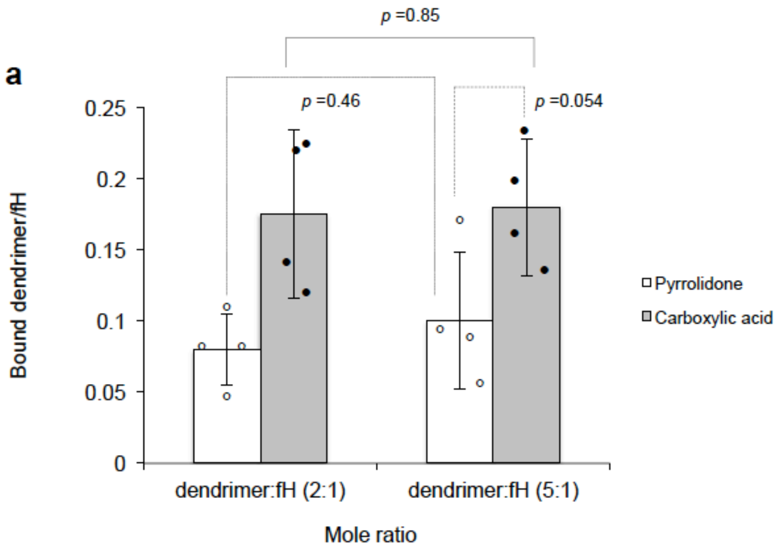

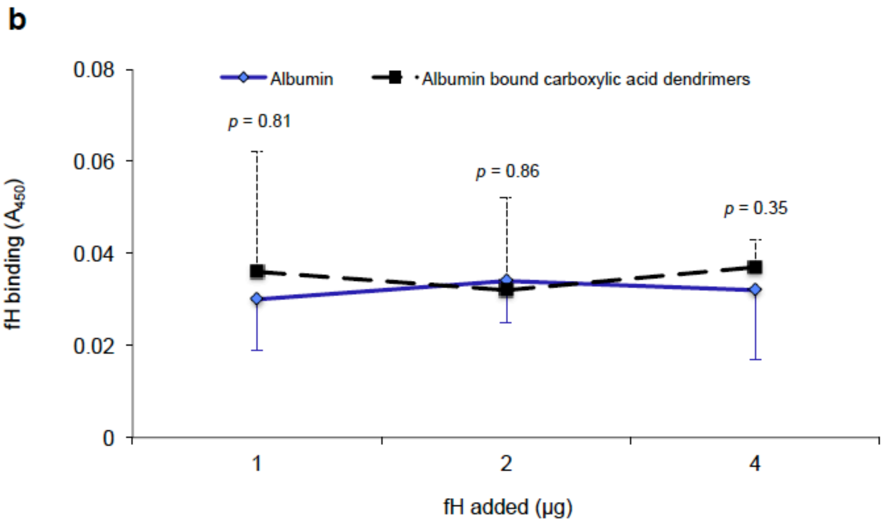

Supplementary Fig. 30. Dendrimer binding to human factor H (fH). **a** Estimation of pyrrolidone- and carboxylic acid-terminated G4 dendrimers binding to human fH. Dendrimers contained a precisely core positioned sulforhodamine B. The results represent mean values and s.d. of four technical replicates, and each in triplicate samples. **b** Evaluation of human fH binding to albumin and albumin bound to carboxylic acid-terminated G4 dendrimers. The results represent mean values and s.d. of three technical replicates, and each in triplicate samples. *p* values refer to unpaired, two-sided student *t*-test.

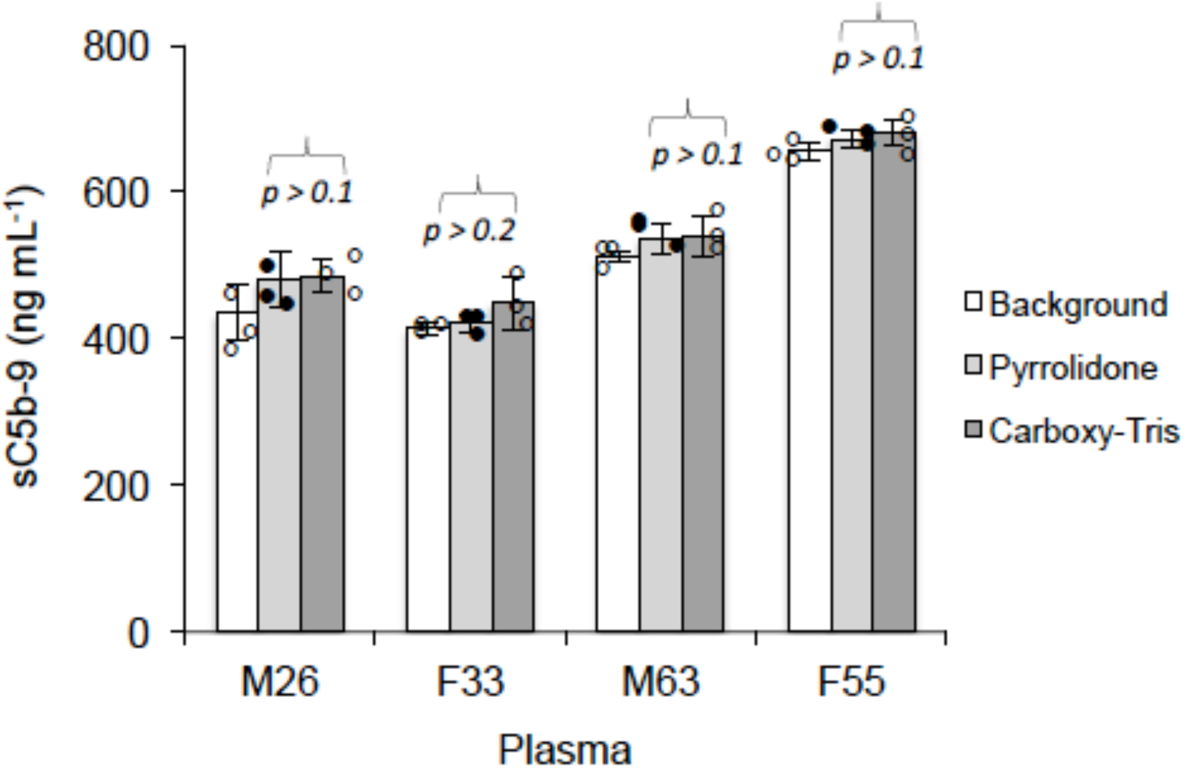

Supplementary Fig. 31. The effect of G5 pyrrolidone and carboxy$_{116}$-Tris$_{12}$ dendrimers on sC5b-9 generation in plasma of 4 healthy 'complement-competent' individuals. Dendrimer concentration = 0.132 mM. Bars show mean values and s.d. of three technical replicates. Each experiment was done in triplicate samples. Differences between groups were examined using ANOVA followed by multiple comparisons (and with Background) with two-tailed Student-Newmann-Keul test.

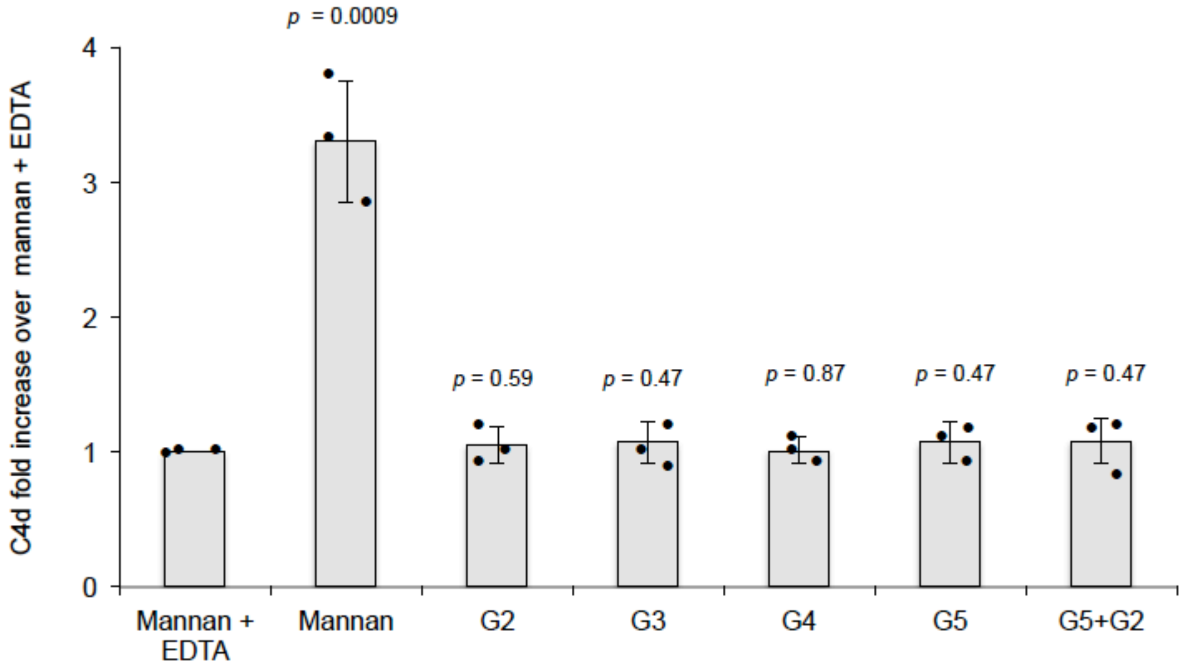

Supplementary Fig. 32. Dendrimers do not generate C4d-containing activation fragments of C4 (C4b, iC4b, and C4d) (collectively referred to as C4d) in a reconstituted upstream lectin pathway. Wells in a microtitre plate were coated either with mannan or different generation (G) of amine-terminated PAMAM dendrimers overnight as described in Methods. G5+G2 refer to wells coated with 80% G5 and 20% G2 dendrimers by weight. Incubations contained MBL/MASPs = 5 μgmL^{-1} MBL, C2 = 30 μgmL^{-1}, C4 = 650 μgmL^{-1}, C4 binding protein = 200 μgmL^{-1} and complement factor I = 35 μgmL^{-1}. After 30 min incubation at 37 °C, fluid phase C4d-conatining fragments was measured by ELISA. C4d folds increases are compared with mannan-coated plates in the presence of EDTA (10 mM). Incubations were in triplicate. The results represent mean values ± s.d. of three technical replicates, and *p* values (unpaired, two-sided) are compared with mannan + EDTA incubation.
