# Supplementary material for: Dendrimer end-terminal motif-dependent evasion of human complement and complement activation through IgM hitchhiking
Source: Nat Commun. 2021 Aug 11;12:4858. doi: 10.1038/s41467-021-24960-6 (PMC8357934; doi:10.1038/s41467-021-24960-6)
Supplement: Supplementary file 2 — Reporting Summary [file 41467_2021_24960_MOESM2_ESM.pdf]

nature research

Corresponding author(s): SM Moghimi

Last updated by author(s): Jul 2, 2021

Reporting Summary

Nature Research wishes to improve the reproducibility of the work that we publish. This form provides structure for consistency and transparency in reporting. For further information on Nature Research policies, see our Editorial Policies and the Editorial Policy Checklist.

Statistics

For all statistical analyses, confirm that the following items are present in the figure legend, table legend, main text, or Methods section.

n/a	Confirmed	
☐	☒	The exact sample size (n) for each experimental group/condition, given as a discrete number and unit of measurement
☐	☒	A statement on whether measurements were taken from distinct samples or whether the same sample was measured repeatedly
☐	☒	The statistical test(s) used AND whether they are one- or two-sided<br>*Only common tests should be described solely by name; describe more complex techniques in the Methods section.*
☒	☐	A description of all covariates tested
☐	☒	A description of any assumptions or corrections, such as tests of normality and adjustment for multiple comparisons
☐	☒	A full description of the statistical parameters including central tendency (e.g. means) or other basic estimates (e.g. regression coefficient) AND variation (e.g. standard deviation) or associated estimates of uncertainty (e.g. confidence intervals)
☐	☒	For null hypothesis testing, the test statistic (e.g. F, t, r) with confidence intervals, effect sizes, degrees of freedom and P value noted<br>*Give P values as exact values whenever suitable.*
☒	☐	For Bayesian analysis, information on the choice of priors and Markov chain Monte Carlo settings
☒	☐	For hierarchical and complex designs, identification of the appropriate level for tests and full reporting of outcomes
☒	☐	Estimates of effect sizes (e.g. Cohen's d, Pearson's r), indicating how they were calculated

Our web collection on statistics for biologists contains articles on many of the points above.

Software and code

Policy information about availability of computer code

Data collection	No software was used for data collection
Data analysis	ASTRA Software (Wyatt Technology); Mnova 10.0 software (Mestrelab); NanoSight NTA software (version 3.2); Microsoft Excel for Mac version 14.7.7; Matlab 2014b (Mathworks, MA, USA)

For manuscripts utilizing custom algorithms or software that are central to the research but not yet described in published literature, software must be made available to editors and reviewers. We strongly encourage code deposition in a community repository (e.g. GitHub). See the Nature Research guidelines for submitting code & software for further information.

Data

Policy information about availability of data

All manuscripts must include a data availability statement. This statement should provide the following information, where applicable:
- Accession codes, unique identifiers, or web links for publicly available datasets
- A list of figures that have associated raw data
- A description of any restrictions on data availability

All original 1H-NMR and 13C-NMR spectra (Supplementary Fig. 1–20; Supplementary Table 1), Fourier transform infrared spectra (Supplementary Fig. 21–23; Supplementary Table 1), UV/Vis and fluorescence spectra (Supplementary Fig. 24; Supplementary Table 1), size exclusion chromatography-multi angular light scattering chromatogram (Supplementary Fig. 25–27; Supplementary Table 1), and mass spectra (Supplementary Methods; Supplementary Table 1) are reported in Supplementary Information file.
Raw data for manuscript Figures 1d, 1e, 1f; 2; 3; 5c, 5d is provided as the Source Data file.
Figures 1a and 2a are schematic drawings. The raw data for Figure 1c appear in Supplementary Table 1 in Supplementary Information File.
Raw data for Supplementary Figures 28, 29, 30, 31, 32 are available from the corresponding author upon reasonable request.

Field-specific reporting

Please select the one below that is the best fit for your research. If you are not sure, read the appropriate sections before making your selection.

- [x] Life sciences
- [ ] Behavioural & social sciences
- [ ] Ecological, evolutionary & environmental sciences

For a reference copy of the document with all sections, see nature.com/documents/nr-reporting-summary-flat.pdf

Life sciences study design

All studies must disclose on these points even when the disclosure is negative.

Sample size	No sample size calculations were performed. The sample size was determined by material available. All analysis indicated either highly significant or clear lack of differences (no significance and no observable trends in the data). Statistical analysis confirmed significance or no significance.
Data exclusions	There are no data exclusions.
Replication	For all experiments, three technical replicates were performed. Biological replicates uses plasma source from nine individuals and presented data shows results from each individual. For most analyses assays were repeated across two or three batches. Statistical analyses used all replicates in a mixed model to appropriately account for all sources of variance. All replications were successful and with with similar trend/ outcome. Clearly defined error bars are presented and where appropriate sd and exact p values are shown.
Randomization	This was not a randomized study. In all experiments, we compared treatment conditions to untreated measurements within the same subject.
Blinding	Researchers were not blinded to experiments or analysis due to complexites in experimental design and large number of positive and negative controls.

Reporting for specific materials, systems and methods

We require information from authors about some types of materials, experimental systems and methods used in many studies. Here, indicate whether each material, system or method listed is relevant to your study. If you are not sure if a list item applies to your research, read the appropriate section before selecting a response.

Materials & experimental systems

n/a	Involved in the study	
☐	☒	Antibodies
☒	☐	Eukaryotic cell lines
☒	☐	Palaeontology and archaeology
☒	☐	Animals and other organisms
☐	☒	Human research participants
☒	☐	Clinical data
☒	☐	Dual use research of concern

Methods

n/a	Involved in the study	
☒	☐	ChIP-seq
☒	☐	Flow cytometry
☒	☐	MRI-based neuroimaging

Antibodies

Antibodies used	For all antibodies catalogue/batch numbers are shown in the Methodology.<br>Human IgM (18260, MFCD00163928) (0.05 M Tris-HCl, 0.2 M NaCl, 15 mM sodium azide, pH 8.0), human IgG (12511, MFCD00163923) (0.01 M sodium phosphate, 0.15 M NaCl, 15 mM sodium azide, pH 7.4), and goat anti-mouse IgG antibody [(H+L) HRP conjugate] (AP308P) were from Sigma-Aldrich (Merck KGaA, Damstadt, Germany).<br>Mouse (IgG1) anti-human C1s monoclonal antibody (HM2108-500 UG) clone m81 was from Hycult Biotech (Uden, The Netherlands).<br>Mouse monoclonal [C18/3] anti-factor H antibody (ab121055) was from Abcam (UK).
Validation	All antibodies were validated by their respective suppliers.<br>https://www.sigmaaldrich.com/GB/en/product/sigma/i8260?context=product#<br>https://www.sigmaaldrich.com/GB/en/product/mm/ap308p?context=product<br>https://www.hycultbiotech.com/hm2108-500ug<br>https://www.abcam.com/factor-h-antibody-c183-ab121055.html<br>Mouse monoclonal anti-factor H antibody was validated by coating plates with human factor H.

Human research participants

Policy information about studies involving human research participants

Population characteristics	Blood donors were five males (age range 26 to 63) and four females (age range 33 to 55). Individual sex and age is identified in data. Ethnicities/races were not included in the sample enrollment.
Recruitment	For blood donation, five males and four females were recruited. Three individuals (2 males and 1 female) had known deficiencies in mannose-binding lectin (MBL). MBL levels were verified by ELISA.
Ethics oversight	Blood samples were collected from volunteered human subjects with informed consent according to the Danish law of blood donation with general approval at Copenhagen University.

Note that full information on the approval of the study protocol must also be provided in the manuscript.
